# An integrative approach to infer systematic relationships and define species groups in the shrub frog genus *Raorchestes*, with description of five new species from the Western Ghats, India

**DOI:** 10.7717/peerj.10791

**Published:** 2021-03-03

**Authors:** Sonali Garg, Robin Suyesh, Sandeep Das, Mark A. Bee, S. D. Biju

**Affiliations:** 1 Systematics Lab, Department of Environmental Studies, University of Delhi, Delhi, India; 2 Department of Environmental Studies, Sri Venkateswara College, University of Delhi, Delhi, India; 3 Forest Ecology and Biodiversity Conservation Division, Kerala Forest Research Institute, Peechi, Kerala, India; 4 EDGE of Existence programme, Conservation and Policy, Zoological Society of London, London, UK; 5 Department of Ecology, Evolution, and Behavior, University of Minnesota - Twin Cities, St. Paul, MN, USA

**Keywords:** Anura, Bioacoustics, Biodiversity hotspot, Diagnostic characters, Eye colour, Integrative taxonomy, Molecular phylogeny, Morphology, Peninsular India, Rhacophoridae

## Abstract

The genus *Raorchestes* is a large radiation of Old World tree frogs for which the Western Ghats in Peninsular India is the major center for origin and diversification. Extensive studies on this group during the past two decades have resolved long-standing taxonomic confusions and uncovered several new species, resulting in a four-fold increase in the number of known *Raorchestes* frogs from this region. Our ongoing research has revealed another five new species in the genus, formally described as *Raorchestes drutaahu* sp. nov., *Raorchestes kakkayamensis* sp. nov., *Raorchestes keirasabinae* sp. nov., *Raorchestes sanjappai* sp. nov., and *Raorchestes vellikkannan* sp. nov., all from the State of Kerala in southern Western Ghats. Based on new collections, we also provide insights on the taxonomic identity of three previously known taxa. Furthermore, since attempts for an up-to-date comprehensive study of this taxonomically challenging genus using multiple integrative taxonomic approaches have been lacking, here we review the systematic affinities of all known *Raorchestes* species and define 16 species groups based on evidence from multi-gene (2,327 bp) phylogenetic analyses, several morphological characters (including eye colouration and pattern), and acoustic parameters (temporal and spectral properties, as well as calling height). The results of our study present novel insights to facilitate a better working taxonomy for this rather speciose and morphologically conserved radiation of shrub frogs. This will further enable proper field identification, provide momentum for multi-disciplinary studies, as well as assist conservation of one of the most colourful and acoustically diverse frog groups of the Western Ghats biodiversity hotspot.

## Introduction

The genus *Raorchestes* Biju, Shouche, Dubois, Dutta, and Bossuyt, 2010 was described to accommodate a large radiation of Asian Shrub frogs currently comprising 67 species with distributions right from the Western Ghats in Peninsular India, up to central, eastern and northeastern India, Nepal, Bangladesh, Myanmar, southern China, Thailand, Malaysia, Laos, Cambodia, and Vietnam (Biju et al., 2010; AmphibiaWeb, 2020; Frost, 2020). The genus is closely related to *Pseudophilautus* Laurent, 1943, another radiation of nearly 80 species chiefly restricted to Sri Lanka with only three recognised members from southern India (Biju et al., 2010; Meegaskumbura et al., 2019). Phylogenetically, the two genera have shown a sister-group relationship (e.g., Li et al., 2009; Yu et al., 2009; Biju et al., 2010; Pyron & Wiens, 2011; Vijayakumar et al., 2016) that has become debatable, especially with recent descriptions of new closely related taxa (e.g., Abraham et al., 2013; Li et al., 2013; Meegaskumbura et al., 2015, 2019; Chan, Grismer & Brown, 2018). Until a few decades ago, *Raorchestes* and *Pseudophilautus* members were included in a single genus *Philautus* Gistel, 1848, which has now mostly been restricted to the Sunda Shelf and Philippines (Biju et al., 2010; Li et al., 2013; Wostl et al., 2017; AmphibiaWeb, 2020; Frost, 2020). The presumed occurrence of genus *Philautus* in India based on literature prior to Biju et al. (2010) (such as, Dubois, 1987; Bossuyt & Dubois, 2001; Delorme et al., 2006), and the inclusion of at least six Indian taxa in *Philautus* thus far (*Philautus dubius* (Boulenger, 1882), *Philautus garo* (Boulenger, 1919), *Philautus kempiae* (Boulenger, 1919), *Philautus kempii* (Annandale, 1912), *Philautus microdiscus* (Annandale, 1912), and *Philautus namdaphaensis* Sarkar & Sanyal, 1985) (Frost, 2020) is erroneous and should be considered uncertain until confirmed by future evidence.

The Western Ghats mountain range in Peninsular India is a major center for the origin and diversification of *Raorchestes* frogs (Biju et al., 2010; Vijayakumar et al., 2016), and it is here that the genus reaches its highest diversity (~80%) (Jiang et al., 2020) with near absolute endemism. Until the end of twentieth century, the diversity of shrub frogs in the Western Ghats comprised only 10 of the presently recognised *Raorchestes* species, which were primarily described by colonial researchers (Jerdon, 1853; Günther, 1876; Boulenger, 1882, 1891; Annandale, 1919) followed by limited post-colonial descriptions (Rao, 1937). It was also common belief that the Western Ghats and Sri Lanka, which together form a single globally recognised biodiversity hotspot unit (Myers et al., 2000; Mittermeier et al., 2004), share several known shrub frogs (Kirtisinghe, 1957; Inger et al., 1984; Dutta & Manamendra-Arachchi, 1996). However, based on extensive field explorations in the Western Ghats, Biju (2001) not only doubted the occurrence of shared species between these regions, suggesting that several confused members likely represent undescribed taxa, but also showed the presence of an unprecedentedly high number of previously undiscovered and new tree frog taxa within the Western Ghats. At the same time, Bossuyt & Dubois (2001) taxonomically reviewed the genus *Philautus sensu lato* resulting in nomenclatural stability and the transfer of several species formerly attributed to various other genera such as *Ixalus*, *Phyllomedusa*, and *Rhacophorus* (Jerdon, 1853, 1870; Günther, 1876; Boulenger, 1882, 1891; Annandale, 1919; Ahl, 1931). Subsequent studies also provided evidence for the fact that the shrub frogs of the Western Ghats and Sri Lanka are endemic to the respective regions, with considerably high undescribed diversity in both regions (Pethiyagoda & Manamendra-Arachchi, 1998; Biju, 2001; Meegaskumbura et al., 2002; Bossuyt et al., 2004). Altogether, what transpired was a spate of new species descriptions from yet unexplored as well as previously explored regions across the Western Ghats, with an ever-increasing estimate of its known shrub frog diversity (Bossuyt, 2002; Kuramoto & Joshy, 2003; Biju & Bossuyt, 2005a, 2006, 2009; Gururaja et al., 2007; Biju et al., 2010). The recognition of *Raorchestes* (Biju et al., 2010) provided further stability to the generic allocations of Asian shrub frogs, with frequent new discoveries holding up the genus as one of the most actively researched anuran groups of the Western Ghats during the following decade (Zachariah et al., 2011, 2016; Seshadri, Gururaja & Aravind, 2012; Padhye et al., 2013; Vijayakumar et al., 2014, 2016; Priti et al., 2016; Gowande, Ganesh & Mirza, 2020). It is no surprise that since the turn of the century 43 new species have been formally described, resulting in a four-fold increase in the number of *Raorchestes* frogs known from this region within just two decades.

Despite active research and frequent descriptions of new species, there has been a lack of integrative understanding of species in this large and rather morphologically conserved group of frogs ever-since the formal description of the genus. Although integrative approaches have increasingly been employed to delimit and describe new species during the past decade (e.g., Vijayakumar et al., 2014; Priti et al., 2016; Zachariah et al., 2016), such studies largely rely on older works based on genus *Philautus* (e.g., Bossuyt & Dubois, 2001; Biju & Bossuyt, 2009) for comparisons with previously known taxa. Vijayakumar et al. (2014, 2016) provided comprehensive phylogenies of Western Ghats *Raorchestes* frogs with lineage-based grouping of species; however, the diagnosis of these phylogenetically identified species assemblages based on morphological, acoustic, or behavioral characters remains unattempted.

Vocalisation in anurans has long been a subject of interest to behavioral ecologists, evolutionary biologists, physiologists (Gerhardt & Huber, 2002; Wells, 2007), and more recently to taxonomists as discussed elaborately in a review by Köhler et al. (2017). Acoustic characters are known to be useful in identification and delimitation of species, and vocalisations all the more conspicuous since they serve as premating isolation mechanisms carrying useful evolutionary and systematic information (Ryan, 2001; Bee, Suyesh & Biju, 2013a; Köhler et al., 2017). As taxonomic studies are increasingly becoming integrative in nature, call characters have gained importance in Indian anuran systematics (e.g., Kanamadi, Kadadevaru & Schneider, 2001; Kuramoto et al., 2007; Grosjean & Dubois, 2011; Bee, Suyesh & Biju, 2013a, 2013b; Garg et al., 2017, 2019). Specifically in the case of genus *Raorchestes*, out of the 55 species known from Peninsular India (prior to the present study), the call structure was previously known only for eleven species, namely *Raorchestes* (as *Philautus*) *tuberohumerus*, *Raorchestes* (as *Philautus*) *luteolus*, *R. kakachi*, *R. graminirupes*, *R. flaviocularis*, *R. chalazodes*, *R. honnametti*, *R. kollimalai*, *R. sanctisilvaticus*, *R. silentvalley*, and *R. lechiya* (Kuramoto & Joshy, 2001; Seshadri, Gururaja & Aravind, 2012; Bee, Suyesh & Biju, 2013b; Vijayakumar et al., 2014; Priti et al., 2016; Zachariah et al., 2016; Mirza et al., 2019; Gowande, Ganesh & Mirza, 2020). Due to lack of available acoustic data for a majority of *Raorchestes* species, vocalisation has not been effectively utilized for integrative systematic studies on this taxonomically challenging genus, and has become imperative for strengthening our understanding of systematic relationships particularly among several morphologically cryptic species.

Several anuran studies have emphasized on the usefulness of eye colour and pattern as a character for species level identification (e.g., Duellman, 1970; Glaw & Vences, 1997; Amat, Wollenberg & Vences, 2013; Glaw et al., 2018) or study of ontogenetic colour changes (e.g., Hoffman & Blouin, 2000; Biju et al., 2013); however, the application of this trait for field identification of frogs is seldom attempted (Glaw & Vences, 1997; Stuebing & Wong, 2000). Among the ~230 known frog species of the Western Ghats, genus *Raorchestes* is the most remarkably diverse in terms of skin colouration as well as eye colours and patterns. This group is also notorious for lacking distinct morphological characters between closely related species and high intraspecific variability in body colour and patterns in some cases, together with the relatively small adult size of its members, which makes sole reliance on morphology-based identification and systematic studies rather challenging (Bossuyt & Dubois, 2001; Biju & Bossuyt, 2009; Vijayakumar et al., 2014). In this backdrop, eye colouration as a character for species-level identification as well as interspecific and group-level comparisons comprehensively across the genus remains overlooked, other than a few new species descriptions (e.g., Gururaja et al., 2007; Biju & Bossuyt, 2009; Vijayakumar et al., 2014; Zachariah et al., 2016).

In this study, we investigate the intrageneric systematic relationships among *Raorchestes* frogs and characterise the phylogenetically identified 16 major species groups (largely congruent with previous studies such as Biju & Bossuyt, 2009; Vijayakumar et al., 2014, 2016), based on morphological (including eye colouration and patterns), acoustic, and associated behavioural traits (such as calling height). Our ongoing research has also revealed another five new species in this genus, all from the State of Kerala in southern Western Ghats, which are formally described on the basis of integrative evidence. In addition, we provide remarks on the taxonomic status of certain poorly known taxa. New insights from this study aim to facilitate a better working taxonomy for this rather large and taxonomically challenging genus, as well as guide future research on ecology, biogeography, evolution, and conservation of its members.

## Materials and Methods

### Field sampling

Field surveys, sampling, and call recordings were carried out primarily during the breeding season of shrub frogs in the Western Ghats (May/June–September) between 2009 and 2019. Adults were found through opportunistic searches or by locating calling males. Sampled individuals were photographed in life followed by euthanisation in Tricaine methanesulphonate (MS-222). Tissue samples were extracted from the thigh muscle, preserved in absolute ethanol, and eventually stored at −20 °C for molecular studies. The specimens were fixed in 4% formalin and transferred to 70% ethanol for preservation. Type specimens are deposited in the Bombay Natural History Society (BNHS), Mumbai, and referred specimens are available at the Systematics Lab, University of Delhi (SDBDU), India. Geographical coordinates of the sampling localities were recorded using a Garmin 76CSx GPS with the WGS84 datum system. Distribution maps were prepared in QGIS version 2.6.1 (http://www.qgis.org).

Fieldwork, including collection of animals in the field, was conducted with permissions and following guidelines from the responsible authorities in the State Forest Departments (Field permits: Nos. WL12-1830/2009, WL10-2606/12, WL10-25421/2014, 67254/2001/WL5, D-22 (8)/Research/4543/2012-13; PCCF(WL)/E2/CR/13/2016–17 and WL10-43756/2015). Research received ethical approval from Department of Environmental Studies, University of Delhi (DES/1020 dated 9 February 2015), India.

### Phylogenetic study

Genomic DNA was extracted from populations sampled from the State of Kerala that were suspected to represent undescribed species, using the Qiagen DNeasy blood and tissue kit (Qiagen, Valencia, CA, USA). The following six gene fragments were PCR-amplified using previously published primers: four mitochondrial—16SrRNA (Simon et al., 1994), 12SrRNA + tRNA^VAL^ (Richards & Moore, 1996), and Cytochrome b (Che et al., 2012); two nuclear—Rhodopsin and Tyrosinase (Bossuyt & Milinkovitch, 2000). The fragments were sequenced on both strands using the BigDye Terminator v3.1 Cycle Sequencing kit (Applied Biosystems) on ABI 3730 automated DNA sequencer (Applied Biosystems). Newly generated sequences were checked and assembled in ChromasPro v1.34 (Technelysium Pty Ltd., St. South Brisbane, QLD, Australia), and deposited in the National Centre for Biotechnology Information (NCBI) GenBank under accession numbers MW020034–MW020035, MW020166–MW020171 and MW023233–MW023244 (Table S1).

Further taxon sampling for phylogenetic studies was carried out by retrieving previously published DNA sequences for vouchers with maximum availability of the analysed genes and as much as possible those representing typical exemplars of all the currently recognised *Raorchestes* species (except *R. thodai*) available in the NCBI GenBank. Additionally, one member (the type species, if available) from all known rhacophorid genera and an outgroup taxon were included in the dataset (Table S1). The datasets for different gene sequences were prepared and aligned for 94 taxa using the ClustalW tool in MEGA 7.0 (Kumar, Stecher & Tamura, 2016). Alignments for coding DNA were checked by comparison with amino acid sequences; non-coding fragments were manually optimised and ambiguous sites were excluded from phylogenetic analyses. The tRNA^VAL^ gene was also excluded from the 12S fragment, due to its non-availability for most of the Genbank sequences. The resultant character set of total 2,327 basepairs was partitioned by genes for the five studied gene fragments and the best-fit models of DNA evolution determined individually by implementing the Akaike Information Criterion in ModelTest 3.4 (Posada & Crandall, 1998) were used for analyses.

Phylogenetic inferences were made under the Maximum Likelihood (ML) criteria. ML searches were performed for 100 independent runs with GTRGAMMA model for each gene partition along with 1,000 thorough bootstrap replicates for assessing the clade support, using RAxML 7.3.0 (Stamatakis, 2006; Stamatakis et al., 2008) in raxmlGUI 1.1 (Silvestro & Michalak, 2012). Further, Bayesian analyses were performed in MrBayes 3.1.2 (Ronquist & Huelsenbeck, 2003) using the best determined model (GTR + I + G) for each gene partition, with two parallel runs of four Markov chain Monte Carlo (MCMC) chains executed for 10 million generations. Trees were sampled after every 1,000 generations and the Bayesian Posterior Probabilities (BPP) for clades were summarised after discarding the first 2.5 million generations as burn-in. Convergence of the parallel runs was confirmed by split frequency standard deviations of less than 0.01 as well as the nearing of potential scale reduction factors to 1.0 for all model parameters using Tracer v1.3 (Rambaut et al., 2014).

### Morphological study

Morphological studies were carried out to compare the populations suspected to represent new species with all previously known *Raorchestes* species and available names, based on examination of available types and other museum specimens, original descriptions, or new topotypic material. All the *Raorchestes* species known from Peninsular India were also comprehensively studied in order to identify shared morphological characters for grouping of species. Sex and maturity were determined by the presence of secondary sexual characters (such as nuptial pads and vocal sacs in males) or examination of gonads. Only adult specimens were used for morphometric studies.

Measurements and associated terminologies follow Biju & Bossuyt (2009). The following measurements were taken to the nearest 0.1 mm by using a digital slide-caliper or a binocular microscope with a micrometer ocular: snout–vent length (SVL), head width (HW, at the angle of the jaws), head length (HL, from rear of mandible to tip of snout), MN (distance from the rear of the mandible to the nostril), MFE (distance from the rear of the mandible to the anterior orbital border), MBE (distance from the rear of the mandible to the posterior orbital border), snout length (SL, from tip of snout to anterior orbital border), eye length (EL, horizontal distance between bony orbital borders), inter upper eyelid width (IUE, the shortest distance between the upper eyelids), maximum upper eyelid width (UEW), internarial distance (IN), internal front of the eyes (IFE, shortest distance between the anterior orbital borders), internal back of the eyes (IBE, shortest distance between the posterior orbital borders), NS (distance from the nostril to the tip of the snout), EN (distance from the front of the eye to the nostril), TYD (greatest tympanum diameter), TYE (distance from the tympanum to the back of the eye), forearm length (FAL, from flexed elbow to base of outer palmar tubercle), hand length (HAL, from base of outer palmar tubercle to tip of third finger), FL_I–IV_ (finger length), thigh length (TL, from vent to knee), shank length (SHL, from knee to heel), foot length (FOL, from base of inner metatarsal tubercle to tip of fourth toe), total foot length (TFOL, from heel to tip of fourth toe), FD (maximum disc width of finger), width of finger (FW, measured at the base of the disc), TD (maximum disc width of toe), width of toe (TW, measured at the base of the disc). Digit number is represented by roman numerals I–V in subscript. All measurements and photographs were taken for the right side of the specimen, except when a character was damaged, in which case the measurement was taken on the left side. All measurements provided in the taxonomy section are in millimetres.

For the convenience of discussion, *Raorchestes* species of the Western Ghats are grouped based on their body size as small (male SVL 17.0–25.0 mm), medium (male SVL 25.1–45.0 mm) and large (male SVL 45.1–65.0 mm). Terminologies for the snout shape follow Heyer et al. (1990). The webbing formulae follow Savage & Heyer (1967) as modified by Myers & Duellman (1982) and followed by Biju et al. (2014), and the degree of webbing relative to subarticular tubercles is described by numbering the tubercles 1–3, starting from the base. Further, the webbing is categorised as basal (slightly above or beyond the basal subarticular tubercles on all toes), small (webbing on toe IV beyond the third subarticular tubercle but below the second subarticular tubercle on either side), medium (webbing on toe IV beyond the second subarticular tubercle but below the first subarticular tubercle on either side), and large (webbing on toe IV extending beyond the first subarticular tubercle on either side), following Garg & Biju (2017). Finger and toe disc morphology types follow Biju et al. (2011).

Using the statistical software Statistica v7.1 (StatSoft Inc.), Principal Component Analysis (PCA) and Discriminant Function Analysis (DFA) were performed to specifically assess the degree of morphological differentiation among the six recognised members of the *Raorchestes bombayensis* group. PCA was performed using 20 morphometric parameters taken from adult males. Factor scores of the first two Principal Components (PC) were observed on a scatterplot. Furthermore, sets of 20 predictor variables were generated from the PCA and all the factor scores were used as input variables for performing a DFA, in order to also determine the classification success of the studied samples.

### Eye colouration and pattern

**Figure 1:**
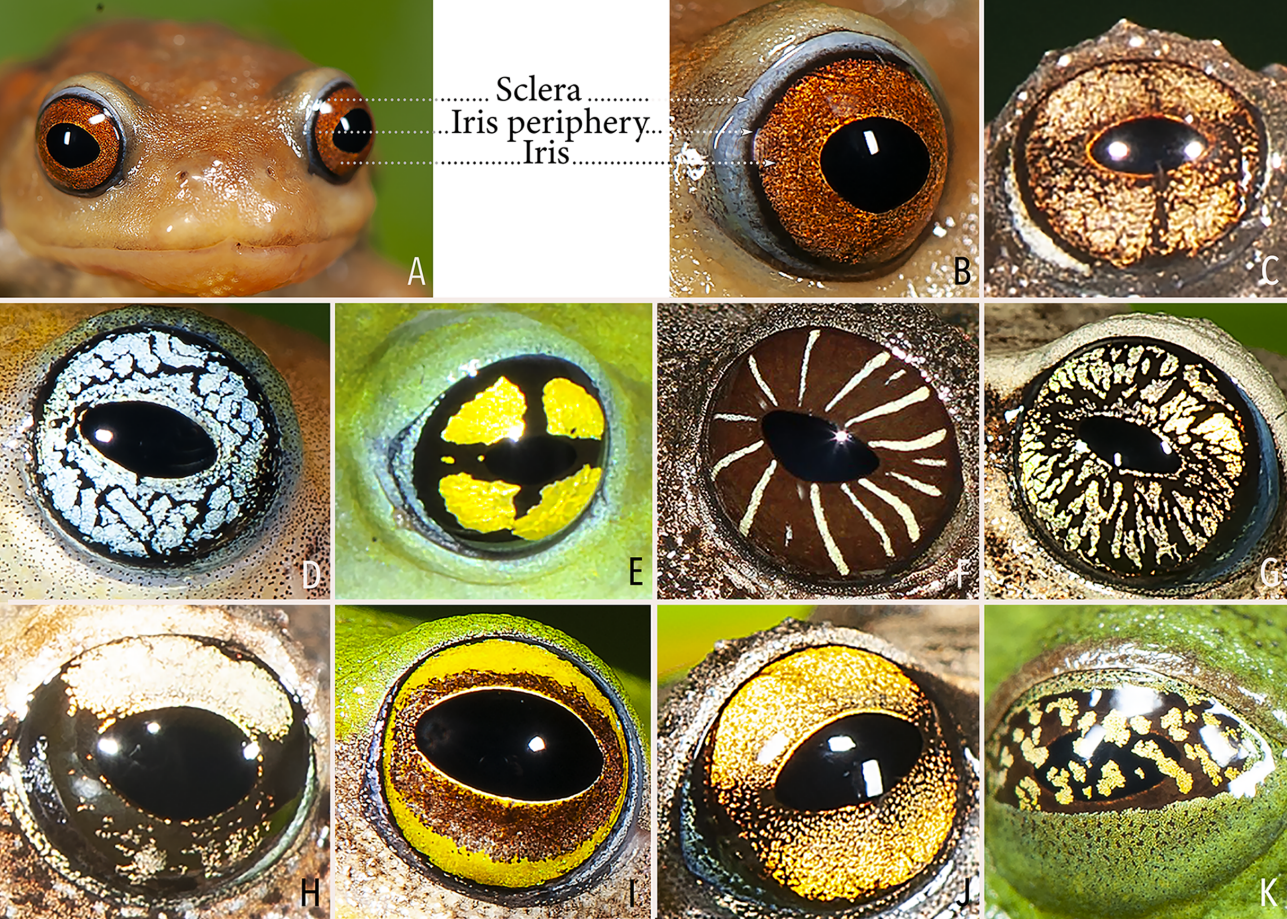
Terminologies for eye structure and the types of eye colours and patterns discussed in the text for *Raorchestes* members. Pupil is rounded and horizontal in all known *Raorchestes* species. (A and B) *R. resplendens* eye marked with sclera, iris periphery, and iris. (C–J) Major types of eye colours and patterns. (C) Iris with horizontal and vertical bands. (D) Black iris with dense metallic silver mosaic pattern. (E) Black iris with golden yellow patches. (F) Brown iris with silver white or golden radiating lines. (G) Brown iris with metallic greenish-yellow reticulations. (H) Iris horizontally divided into light upper and dark lower halves. (I) Yellow iris with an inner reddish-brown ring. (J) Brown iris with dense golden yellow speckling. (K) Reddish-brown iris with yellowish-green spots and blotches on the palpebral membrane.

We made a dedicated effort to photograph and document the colour and pattern of the eyes of all the *Raorchestes* species in the Western Ghats from various angles. All the photographs were taken with the aid of external flashlight, either during the day or night. The interpretations for eye colour and pattern in this study are solely based on photographs. The possibility of variations in eye colour were also observed under captivity before photography. However, 19 randomly tested species did not show significant variation in eye colour, unlike the changes usually observed in the case of dorsal skin colouration. The following parameters were described: (1) eye colour of individual species; (2) comparisons with morphologically and phylogenetically related species. Terminologies for the eye structures (Fig. 1) are adopted and modified from Glaw & Vences (1997).

### Call recordings and acoustic analyses

The sound recordings of 43 species (representing 14 species groups) were made at night when the animals were actively calling (18:00–04:00 h). Calls were recorded in the field using a Sennheiser microphone ME 66 connected or MKH 416 directional microphone connected to a digital solid-state recorder, such as Marantz PMD620, Marantz PMD670, Fostex FR2LE, Zoom H6 and Zoom H4n (44.1 kHz sampling rate, 16-bit resolution) and monitored in real time using Sony MDR V500 headphones. The gain settings of the recorder were adjusted prior to each recording to avoid clipping the amplitude envelopes of recorded calls and maintained throughout to ensure a constant signal to noise ratio within a recording.

Acoustic properties were measured using Raven Pro v1.4 (Charif, Waack & Strickman, 2010). Our use of terminology to describe species-specific vocal repertoires follows our earlier reports describing the vocalizations of *Pseudophilautus kani* and *Raorchestes graminirupes* (Bee, Suyesh & Biju, 2013a, 2013b), and readers are referred to those studies for additional details not described here. Briefly, the members of genus *Raorchestes* produced one to three types of call, which either had pulsatile or non-pulsatile temporal structures. Calls could be produced singly or organised into longer “call groups” (a series of calls delivered in quick succession separated by a short time interval from a subsequent call group). Call groups consisted of either repetitions of the same call type or a mixture of different call types. We labelled calls as Type 1 (“Type 1” calls were the most frequently delivered call type or if two different call types were delivered together as groups then the first delivered call in a call group was named as “Type 1”) and Type 2 and Type 3 for the species producing more than one call type. For species with pulsatile calls, we analysed five temporal properties (call duration, call rise time, call fall time, number of pulses per call, and pulse rate) and one spectral property (overall dominant frequency). Three temporal properties (call duration, call rise time, and call fall time) and one spectral property (overall dominant frequency) were used for analyses for species with non-pulsatile calls. We would note that in addition to among-species variation, the temporal and spectral properties of anuran vocalisations also vary among individuals within species and within individuals, for example, as a function of differences in temperature and social context (Gerhardt & Huber, 2002). The values reported below do not take into consideration these sources of call variation, which are to be expected to operate within each species.

For visual representations of calls, oscillograms showing the amplitude versus time waveform were prepared using a time frame of 1 s for species groups (*n* = 8) that produce calls/call groups longer than 0.1 s and a time frame of 0.1 s for species groups (*n* = 6) that produce calls shorter than 0.1 s. The overall dominant frequency information for the calls of each species was obtained using Raven’s spectrogram function after selecting the entire duration of the call (1,024-point fast Fourier transform, Hann window, 50% overlap, 43.1 Hz resolution). Spectrograms were prepared for graphical representation of the call spectrum at similar time frames as the oscillograms.

### New species names

The electronic version of this article in Portable Document Format (PDF) will represent a published work according to the International Commission on Zoological Nomenclature (ICZN), and hence the new names contained in the electronic version are effectively published under that Code from the electronic edition alone. This published work and the nomenclatural acts it contains have been registered in ZooBank, the online registration system for the ICZN. The ZooBank LSIDs (Life Science Identifiers) can be resolved and the associated information viewed through any standard web browser by appending the LSID to the prefix http://zoobank.org/. The LSID for this publication is: urn:lsid:zoobank.org:pub:7021B266-C54A-4E64-8645-AACCBFBB1A72. The online version of this work is archived and available from the following digital repositories: PeerJ, PubMed Central and CLOCKSS.

## Results

### Phylogenetic relationships

**Figure 2:**
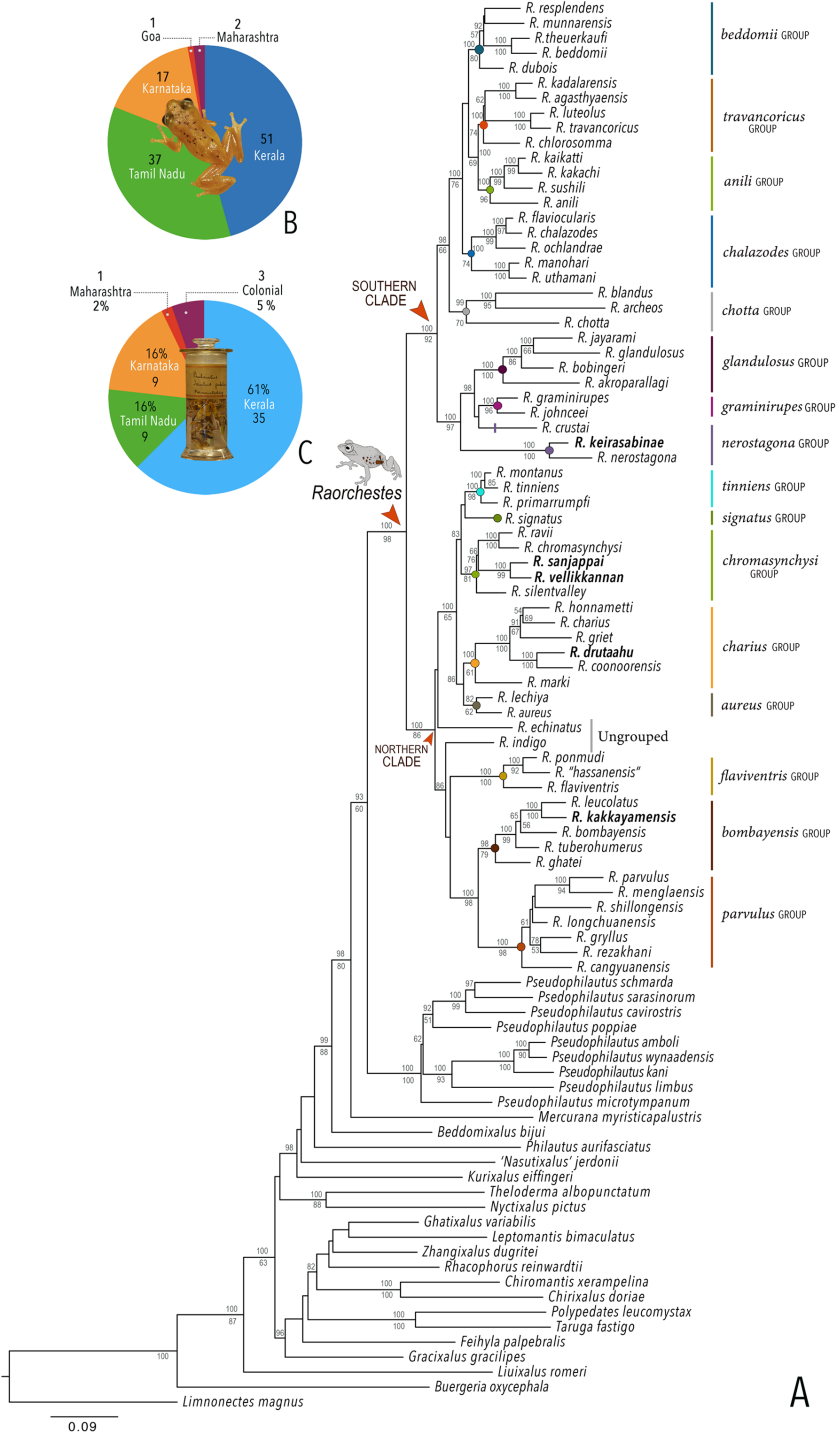
Phylogenetic relationships in the genus *Raorchestes* and State-wise figures for species in the Western Ghats. (A) Maximum Likelihood phylogram, based on 2,327 bp partitioned dataset for three mitochondrial and two nuclear gene fragments from 94 taxa, showing phylogenetic relationships among 60 previously recognised and five new *Raorchestes* species along with representatives of other known rhacophorid genera. The focal genus *Raorchestes* comprises 16 major species-groups discussed in the study. New species described in the study are indicated in bold letters. The values above and below the branches indicate Bayesian Posterior Probabilities (BPP) and RAxML Bootstrap support (BS), respectively. (B) Number of *Raorcheste*s species reported from the Indian States encompassing the Western Ghats. (C) Proportion of the currently recognised species originally described from each state. Descriptions from regions with colonial names that may include more than one State are categorised separately.

Our Maximum Likelihood (ML) and Bayesian phylogenetic analyses (Fig. 2) recovered genus *Raorchestes* as a well-supported monophyletic clade, showing a sister-group relationship with the genus *Pseudophilautus* (e.g., Li et al., 2009; Yu et al., 2009; Biju et al., 2010; Pyron & Wiens, 2011; Vijayakumar et al., 2016). The focal genus showed two major radiations, the northern clade and the southern clade (Vijayakumar et al., 2016), that were further divided into 16 major sub-clades largely congruent with Vijayakumar et al. (2014) and hereafter referred to as species groups, as indicated with modifications in Fig. 2. Most of the recognised species groups were recovered with high (BPP ≥ 95, BS ≥ 70) support, except for the *Raorchestes charius* group and *R. aureus* group that received moderate support probably due to the phylogenetic position of *R. marki* (included in *R. charius* species group in the present study; in *R. aureus* sub-clade as per Vijayakumar et al., 2016; unresolved in Vijayakumar et al., 2014). The relationships of three species (*R. crustai*, *R. echinatus*, and *R. indigo*) also remained unresolved, as shown previously by Vijayakumar et al. (2014, 2016). While *R. crustai* appreared to show a closer but unsupported phylogenetic affinity to members of the *R. graminirupes* group, we provisionally assign it to the *R. nerostagona* group based on additional morphological and acoustic evidence (see ‘Grouping of species using integrative approaches’), until further evidence proves otherwise. On the other hand *R. echinatus* and *R. indigo* are treated as ungrouped species (see ‘Grouping of species using integrative approaches’ for discussion on morphological affinities). Although the phylogenetic relationships at the species-level were often well-supported, several lineages were also either moderately to weakly support or remained unresolved (Fig. 2).

In addition to the previously known *Raorchestes* species, our study also included five populations representing potential candidate species (Fig. 2). Our analyses concordantly supported the distinct phylogenetic position of all these lineages in four recognised species groups (one in *R. bombayensis* group; one in *R. charius* group; two in *R. chromasynchysi* group; and one in *R. nerostagona* group) with well-supported sister-group relationships. Based on additional integrative evidence, we confirm that these putative lineages represent distinct new species and are formally described below.

### Description of new species

***Raorchestes drutaahu*** sp. nov.

http://zoobank.org/urn:lsid:zoobank.org:act:4B07924D-80B3-468B-8C35-C3A8C3001E23

**Figure 3:**
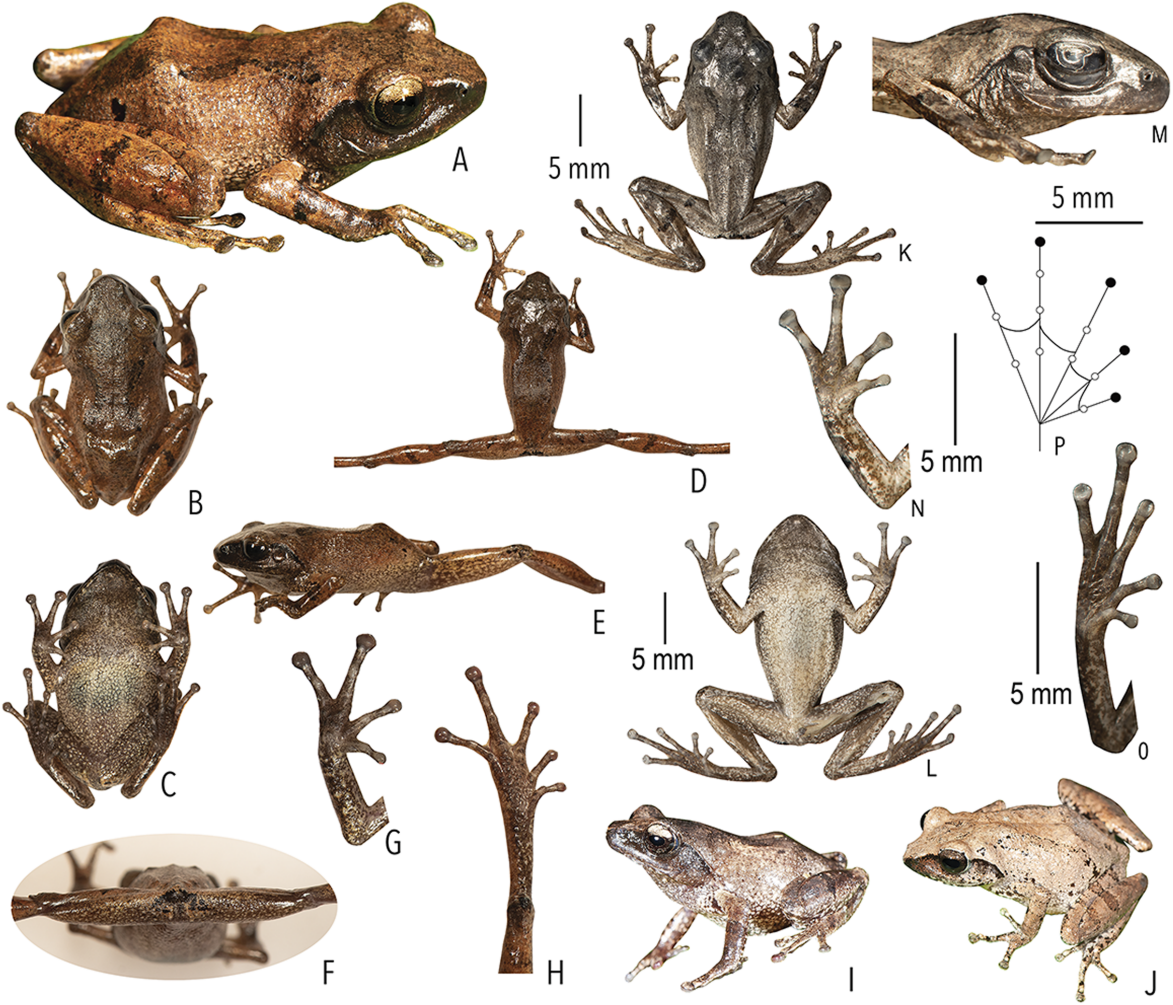
Type and referred specimens of *Raorchestes drutaahu* sp. nov. (A–H) Holotype, in life (BNHS 6088, adult male). (A) Dorsolateral view. (B) Dorsal view. (C) Ventral view. (D) Dorsal view of body and thighs. (E) Lateral view. (F) Posterior view of thighs. (G) Ventral view of hand. (H) Ventral view of foot. (I–J) Paratypes, in life. (I) Dorsolateral view (BNHS 6089, adult male). (J) Dorsolateral view (SDBDU 2015.3025, adult female). (K–P) Holotype, in preservation (BNHS 6088, adult male). (K) Dorsal view. (L) Ventral view. (M) Lateral view of head. (N) Ventral view of hand. (O) Ventral view of foot. (P) Schematic illustration of webbing on foot.

Fast-calling Shrub Frog

(Figs. 2–3; Tables 1–3; Tables S1 and S2)

**Table 1:** Dorsal colouration and eye characters in 59 *Raorchestes* species of Peninsular India.

Group/Species	Dorsum		Eye colouration and markings		
	Colour	Markings	Iris	Iris periphery	Sclera
***Raorchestes anili* group**					
*Raorchestes anili*	Light to dark brown	Dark brown inverted ‘V’-shaped mark	Light golden brown with reddish tinge	Dark brown	Light blue
*Raorchestes kaikatti*	Greyish or reddish-brown	With or without inconspicuous dark spots and markings	Brown or reddish-brown to orange	Dark brown	Light blue
*Raorchestes kakachi*	Light to dark brown or light grey	With or without dark irregular spots and markings	Dark brown or reddish-brown	Dark brown	Light blue
*Raorchestes sushili*	Brown, greyish or reddish-brown	Dark inverted ‘V’-shaped mark and irregular spots	Brown, copper, or reddish-brown	Dark brown	Light blue
***Raorchestes aureus* group**					
*Raorchestes aureus*	Light brown or pale yellow	Absence of prominent markings	Golden brown	Black	Light blue
*Raorchestes lechiya*	Light brown or greyish-brown	With or without dark bands, spots or markings	Golden brown	Black	Light blue
***Raorchestes beddomii* group**					
*Raorchestes beddomii*	Bright green or yellowish-green	Absence of prominent markings	Red, brick red, or orange	Black	Light blue
*Raorchestes dubois*	Highly variable from white, grey, green, brown, yellow to red	With or without contrasting and variable spots, streaks, bands or markings	Light golden brown with reddish tinge	Black	Scarlet blue
*Raorchestes munnarensis*	Brown to yellowish-grey	Dark inverted ‘V’ or ‘X’-shaped mark	Light brown to coffee brown	Black	Scarlet blue
*Raorchestes resplendens*	Reddish-orange interspersed with black	Multiple prominent bright orange macroglands	Red or brick red	Black	Scarlet blue
*Raorchestes theuerkaufi*	Brown or reddish brown	Irregular dark mottling and scattered patches	Light golden brown or copper	Black	Light blue
***Raorchestes bombayensis* group**					
*Raorchestes bombayensis*	Brown or greyish-brown	With or without inverted ‘V’ or ‘X’-shaped mark	Brown with dense golden speckling, and dark brown horizontal and vertical bands	Dark brown	Light silvery blue
*Raorchestes ghatei*	Brown or greyish-brown	With or without inconspicuous dark bands (‘V’ or ‘X’-shaped), spots or markings	Brown with dense golden speckling, and dark brown horizontal and vertical bands	Dark brown	Light silvery blue
*Raorchestes kakkayamensis* sp. nov.	Brown or reddish-brown	Dark discontinuous concave bands and scattered darks streaks or markings	Brown with dense golden speckling, and dark brown horizontal and vertical bands	Dark brown	Light silvery blue
*Raorchestes leucolatus*	Brown to reddish-brown	Scattered orange spots or patches and inconspicuous dark markings	Brown with dense golden speckling, and dark brown horizontal and vertical bands	Dark brown	Light silvery blue
*Raorchestes sanctisilvaticus*	Brown or greyish-brown	With or without inverted ‘V’ or ‘X’-shaped mark	Brown with dense golden speckling, and dark brown horizontal and vertical bands	Dark brown	Light silvery blue
*Raorchestes tuberohumerus*	Light to dark brown	Faint to prominent dark X-shaped mark or irregular markings	Brown with dense golden speckling, and dark brown horizontal and vertical bands	Dark brown	Light silvery blue
***Raorchestes chalazodes* group**					
*Raorchestes chalazodes*	Green, yellowish or bluish-green	Rarely with scattered spots	Black with a golden yellow ring that may or may not be divided by a black cross mark	Black	Scarlet blue
*Raorchestes flaviocularis*	Green or reddish green	Lichen pattern exposing reddish fleshy skin	Black with a golden yellow ring that may or may not be divided by a black cross mark	Black	Indistinct
*Raorchestes ochlandrae*	Brown or reddish-brown	Light yellow dorsolateral bands with or without elongate blotches or scattered spots	Black with light yellow patches in a radial pattern	Black	Indistinct
*Raorchestes manohari*	Bright yellow to greyish-yellow	Scattered dark brown spots	Black with dense metallic silver mosaic pattern	Black	Indistinct
*Raorchestes uthamani*	Yellow with grey or red tinge	With or without faint dark brown streaks or spots	Black with dense metallic silver mosaic pattern	Black	Indistinct
***Raorchestes charius* group**					
*Raorchestes charius*	Brown or reddish-brown	Contrasting concave bands and irregular patches	Light or dark brown with golden tinge, horizontally divided into light upper and dark lower halves	Blackish-brown	Light grey
*Raorchestes coonoorensis*	Light brown or reddish-brown	With or without, continuous or discontinuous dark concave bands	Brown with golden tinge, horizontally divided into light upper and dark lower halves	Blackish-brown	Light blue
*Raorchestes drutaahu* sp. nov.	Light to dark brown or straw	With or without, continuous or discontinuous grey bands or stripes	Brown with golden tinge, horizontally divided into light upper and dark lower halves	Blackish-brown	Light grey
*Raorchestes griet*	Brown with grey or reddish tinge	Irregular black patches or dark concave bands	Brown with golden tinge, horizontally divided into light upper and dark lower halves	Black	Light grey
*Raorchestes honnametti*	Brown or grey	Faint to prominent contrasting concave bands	Brown with golden tinge, horizontally divided into light upper and dark lower halves	Black	Light blue
*Raorchestes kollimalai*	Light or dark brown	Faint to prominent, continuous or discontinuous contrasting concave bands	Brown with golden tinge, horizontally divided into light upper and dark lower halves	Black	Light blue
*Raorchestes marki*	Grey, brown or reddish-brown	Dark X-shaped mark or a pair of concave bands	Brown with dense golden yellow speckling, and with brown horizontal and vertical bands	Black	Light bluish-grey
***Raorchestes chotta* group**					
*Raorchestes archeos*	Greyish, reddish or yellowish-brown	With or without dark broad median band	Golden brown, with dark vertical band	Brown	Greyish-white
*Raorchestes blandus*	Greyish to reddish-brown	Irregular dark brown and orange patches	Golden brown	Brown	Greyish-white
*Raorchestes chotta*	Brown with yellow or grey tinge	Irregular dark brown blotches or scattered dark spots	Golden brown	Brown	Greyish-white
***Raorchestes chromasynchysi* group**					
*Raorchestes chromasynchysi*	Brown or Green	With or without contrasting dark bands, streaks or markings	Golden brown with reddish tinge	Blackish-brown	Scarlet blue
*Raorchestes ravii*	Brown or orangish-brown	With or without dark median band, faint X-shaped mark or scattered spots	Golden brown	Dark brown	Scarlet blue
*Raorchestes sanjappai* sp. nov.	Green	Without prominent dark markings, occasionally with markings	Reddish-brown	Dark brown	Light blue
*Raorchestes silentvalley*	Green	With or without yellow or bluish-black spots	Brown or dark red	Blackish-brown	Light blue
*Raorchestes vellikkannan* sp. nov.	Brown or pale yellow	Dark brown ‘X’-shaped mark and scattered spots	Silver grey with minute brown speckling	Dark brown	Light blue
***Raorchestes flaviventris* group**					
*Raorchestes flaviventris*	Green or yellowish-green	With or without scattered pale yellow, golden yellow, or white spots	Creamy white with minute brown speckles	Dark brown	Bluish-grey
*Raorchestes ponmudi*	Brown, reddish-brown or light to dark grey	Dark brown concave bands or X-shaped mark, with or without few scattered white blotches and minute black spots	Light brown or golden brown with minute dark speckles	Dark brown	Bluish-grey
***Raorchestes glandulosus* group**					
*Raorchestes akroparallagi*	Shades of green, yellow, brown, reddish-brown, or light grey	With or without yellow dorsolateral streaks, scattered small grey or large blackish-brown spots	Light brown to reddish-brown with golden speckles	Blackish-brown	Light blue
*Raorchestes bobingeri*	Green	Without prominent markings	Light yellow or greyish-yellow with an inner brown ring or irregular spots	Blackish-brown	Light blue
*Raorchestes glandulosus*	Green, greenish-yellow, brown, reddish-brown, or purplish	With or without uniformly scattered contrasting spots and reticulations	Bright to dark red, or reddish-brown with golden speckles	Blackish-brown	Light blue
*Raorchestes jayarami*	Green, bluish-green or yellow	With or without uniformly scattered contrasting spots or irregular streaks	Bright yellow or greyish-yellow with an inner reddish-brown ring or spots	Blackish-brown	Light blue
***Raorchestes graminirupes* group**					
*Raorchestes graminirupes*	Brown or yellow with grey or reddish tinge	Dark irregular patches, streaks, or longitudinal bands	Greyish-brown with dense metallic silver or light brown speckles, with scarlet blue ring	Black	Light silvery blue
*Raorchestes johnceei*	Brown, grey, pale yellow, or reddish	Dark irregular patches, broad median band, pair of continuous or discontinuous concave bands or inverted V-shape mark	Light greyish-brown with dense metallic silver or light brown speckles, with scarlet blue ring	Black	Light silvery blue
***Raorchestes nerostagona* group**					
*Raorchestes crustai*	Brown, greyish-brown or green	Irregular dark brown or greenish-brown blotches, continuous or discontinuous concave bands, or inverted V-shape mark	Light greyish-brown	Dark brown	Scarlet blue
*Raorchestes keirasabinae* sp. nov.	Brown, greyish-brown or greenish-brown	Irregular brown, black and green patches	Reddish-grey with faint or prominent horizontal brown band	Dark brown	Scarlet blue
*Raorchestes nerostagona*	Brown or greyish-green	Irregular dark green, reddish-brown, or bluish-black patches of various size	Reddish-grey with faint or prominent horizontal brown band	Dark brown	Scarlet blue
***Raorchestes signatus* group**					
*Raorchestes signatus*	Brown, grey, or red	With or without, faint to prominent, dark ‘X’-shape or inverted V-shape mark, contrasting broad median band, or scattered dark spots and patches	Reddish-brown with or without silver white or golden radiating lines and golden speckling	Without prominent ring	Greyish-brown
***Raorchestes tinniens* group**					
*Raorchestes montanus*	Light brown to chocolate brown, pinkish or reddish-brown, with metallic tinge in all morphs	With or without dark streaks, concave bands, X-shape mark, or variable mosaic patterns	Dark brown or golden brown	Black	Ash grey
*Raorchestes primarrumpfi*	Brown, grey, reddish-brown, or greenish-brown, with metallic tinge in all morphs	With or without dark irregular patches or contrasting yellowish spots	Dark brown or greyish-brown	Black	Ash grey
*Raorchestes tinniens*	Brown, grey, reddish-brown, or greenish-brown, with metallic tinge in all morphs	With or without dark irregular patches or contrasting yellowish spots	Dark brown with golden brown speckling	Black	Ash grey
***Raorchestes travancoricus* group**					
*Raorchestes agasthyaensis*	Brown, greyish-brown, or reddish-brown	Dark inverted V-shape mark with scattered dark patches or dark brown broad median band	Dark brown with dense golden speckling, and horizontally divided into light upper and dark lower halves	Dark brown	Ash grey
*Raorchestes chlorosomma*	Brown or grey	Continuous or discontinuous dark concave bands or irregular streaks, or dark broad median band	Metallic greyish-green or greenish-yellow with dark brown reticulations	Dark brown	Scarlet blue
*Raorchestes kadalarensis*	Brown, greyish-brown, reddish-brown, or bluish-brown	Dark inverted V-shape mark	Dark brown with dense golden speckling, and horizontally divided into light upper and dark lower halves	Dark brown	Ash grey
*Raorchestes luteolus*	Yellow, reddish-yellow, or yellowish-brown	Faint, continuous or discontinuous, dark longitudinal lines or without prominent markings	Golden yellow or light grey with brown speckling with a cobalt blue outsider ring	Black or bluish-black	Indistinct
*Raorchestes travancoricus*	Red or reddish-brown	Prominent longitudinal dark lines	Golden yellow or light grey with brown speckling with a cobalt blue outsider ring	Black or bluish-black	Indistinct
**Ungrouped species**					
*Raorchestes indigo*	Green or greenish-yellow	With or without irregular and scattered black, yellow or bluish-black spots	Golden brown	Dark brown	Light blue
*Raorchestes echinatus*	Brown, greyish-brown, or reddish-brown	Thin middorsal line, irregular dark streaks and scattered minute dark spots	Golden brown with dark vertical band	Dark brown	Indistinct

**Note:**

Two species are not assigned to any species group. Values are calculated for a typical single call of each species. Broad calling height categories: Ground (ground and associated grass): 0–0.5 m; Low (low bushes and shrubs): 0.5–1.5 m; Medium 1 (high shrubs): 1.5–4 m; Medium 2 (lower canopy): 4–7 m; Canopy (high canopy): 7 m and above (up to 40 m).

**Table 2:** Acoustic properties and calling height in 59 *Raorchestes* species of Peninsular India summarised in 15 species groups.

Group/species	Call delivery pattern	Call type	Temporal properties						Spectral properties		Calling height (meters)
			Temporal structure	Call duration (ms)	Call rise time (ms)	Call fall time (ms)	Pulses/call	Pulse rate (Pulses/sec)	Broad frequency peaks	Dominant frequency (kHz)	
***Raorchestes anili* group**		**1–2 types**	**Pulsatile**	**51.2–980.4**	**Nil–960.2**	**Nil–48.8**	**5–22**	**6.3–250.8**	**Single**	**2.4–2.9**	**1.5–5**
*R. anili*	Delivered in groups	Type 1	Pulsatile, widely spaced pulses	980.4	960.2	20.2	7	6.3	Single	2.9	1.5–5
*R. kaikatti*	Delivered in groups, fixed call order (Type 1 followed by Type 2)	Type 1	Pulsatile, more closely packed pulses than Type 2	102.7	67.5	Not significant	22	250.8	Single	2.4	1.5–5
		Type 2	Pulsatile, more widely packed pulses than Type 1	68.9	Not significant	14.4	5	89.3	Single	2.4	
*R. sushili*	Uniform intervals, delivered in groups	Type 1	Pulsatile, closely spaced pulses	51.2	Not significant	48.8	11	242.8	Single	2.6	1.5–5
*R. kakachi*	not studied										1.5–3
***Raorchestes beddomii* group**		**1–3 types**	**Pulsatile or Non-pulsatile**	**13.2–498.2**	**1.0–475.3**	**Nil–49.2**	**1–15**	**Nil–406.5**	**1–3**	**2.2–2.7**	**0–20**
*R. beddomii*	Delivered in groups (only Type 2 call)	Type 1	Pulsatile	150.0	140.2	11.2	4	29.2	Single	2.6	0.5–2
		Type 2	Non-pulsatile	13.2	1.1	12.0	1	–	Single	2.6	
*R. dubois*	Generally uniform intervals, not delivered in groups	Type 1	Non-pulsatile	10.6	1.0	8.3	1	–	Single	2.7	0–2
*R. munnarensis*	Delivered in groups, lack any fixed call order	Type 1	Pulsatile, pulses more widely spaced than Type 2	498.2	475.3	Not significant	3	6.4	Single	2.2	1.5–20
		Type 2	Pulsatile, pulses more closely packed than Type 1	22.8	7.7	14.4	5	406.5	Single	2.2	
*R. resplendens*	Delivered in groups	Type 1	Non-pulsatile	51.3	16.7	49.2	1	–	Three	2.5	
		Type 2	Pulsatile	28.7	1.5	27.1	7	269.2	Two	2.7	0–1
		Type 3	Non-pulsatile	64.7	41.6	24.7	1	–	Four	2.7	
*R. theuerkaufi*	Not studied										0.5–3
***Raorchestes bombayensis* group**		**1 type**	**Non-pulsatile**	**11.6–29.4**	**1.2–9.2**	**9.5–25.4**	**1**	**–**	**Single**	**2.9–4.1**	**0.5–5**
*R. bombayensis*	Not delivered in groups	Type 1	Generally single pulse	11.6	1.2	9.5	1	–	Single	3.1	2–5
*R. ghatei*	Not delivered in groups	Type 1	Generally single pulse	17.7	1.5	16.0	1	–	Single	2.9	1–4
*R. kakkayamensis* sp. nov.	Not delivered in groups	Type 1	Generally single pulse	25.3	9.2	15.9	1	–	Single	3.8	1–3
*R. leucolatus*	Not delivered in groups	Type 1	Generally single pulse	29.4	2.7	25.4	1	–	Single	4.1	1–4
*R. sanctisilvaticus*	Not delivered in groups	Type 1	Generally single pulse	12.2	1.3	10.8	1	–	Single	3.1	1–3
*R. tuberohumerus*	Not delivered in groups	Type 1	Generally single pulse	14.6	1.3	13.3	1	–	Single	3.3	0.5–2
***Raorchestes chalazodes* group**		**1 type**	**Non-pulsatile**	**18.4–36.0**	**1.2–4.1**	**11.0–32.3**	**1**	**–**	**Single**	**2.7–3.6**	**1–7**
*R. chalazodes*	Rapidly delivered in long call groups	Type 1	Non-pulsatile	18.4	2.9	15.2	1	–	Single	2.7	1.5–7
*R. ochlandrae*	Rapidly delivered in long call groups	Type 1	Non-pulsatile	25.3	4.1	20.9	1	–	Single	2.7	1.5–7
*R. manohari*	Rapidly delivered in long call groups	Type 1	Non-pulsatile	12.3	1.2	11.0	1	–	Single	3.6	1–7
*R. uthamani*	Rapidly delivered in long call groups	Type 1	Non-pulsatile	36.0	3.2	32.3	1	–	Single	3.4	1–7
*R. flaviocularis*	Not studied										1.5–7
***Raorchestes charius* group**		**1 type**	**Pulsatile**	**24.6–92.6**	**1.2–59.4**	**16.2–49.1**	**3–17**	**89.3–266.6**	**Single**	**2.4–4.1**	**0–4**
*R. charius*	Uniform intervals, not delivered in groups	Type 1	Pulsatile, relatively short and closely packed pulses	92.6	49.2	37.2	17	226.6	Single	2.4	0–1.5
*R. griet*	Uniform intervals, not delivered in groups	Type 1	Pulsatile, relatively short and closely packed pulses	75.6	59.4	16.2	10	151.3	Single	3.5	0.5–1.5
*R. honnametti*	Uniform intervals, not delivered in groups	Type 1	Pulsatile, relatively short and closely packed pulses	68.6	13.3	44.2	6	89.3	Single	2.6	0.5–1.5
*R. coonoorensis*	Not delivered in groups	Type 1	Pulsatile, relatively short and closely packed pulses	24.6	1.2	22.2	3	200	Single	2.9	0.5–1.5
*R. drutaahu* sp. nov.	Not delivered in groups	Type 1	Pulsatile, relatively short and closely packed pulses	50.6	1.2	49.1	6	134.5	Single	3.6	0.5–1.5
*R. kollimalai*	Not studied										1–4
*R. marki*	Not delivered in groups	Type 1	Pulsatile, closely packed pulses	36.7	1.3	32.6	4	266.6	Single	4.1	1.5–3
***Raorchestes chotta* group**		**1–2 types**	**Pulsatile or Non-pulsatile**	**17.1–71.2**	**1.1–38.3**	**15.0–18.8**	**1–19**	**Nil–382.1**	**Single**	**3.1–3.6**	**0–4**
*R. archeos*	Uniform intervals, delivered in groups	Type 1	Pulsatile, relatively short and closely packed pulses	19.7	1.2	17.2	6	382.1	Single	3.1	0–2
*R. blandus*	Delivered in groups (only Type 1 call), lack any fixed call order	Type 1	Pulsatile	17.1	1.5	15.8	3	370.3	Single	3.5	0.5–4
		Type 2	Non-pulsatile	20.1	1.4	18.8	1	–	Single	3.5	
*R. chotta*	Delivered in groups, fixed call order (Type 1 followed by Type 2)	Type 1	Pulsatile	71.2	38.3	18.6	19	283.5	Single	3.6	0–2
		Type 2	Non-pulsatile	17.2	1.1	15.0	1	–	Single	3.6	
***Raorchestes chromasynchysi* group**		**1 type**	**Pulsatile**	**411.2–716.0**	**345.2–693.8**	**Nil–35.1**	**2–6**	**2.7–7.2**	**Single**	**2.2–2.5**	**0.5–6**
*R. chromasynchysi*	Not delivered in groups	Type 1	Pulsatile, widely spaced pulses	381.4	345.2	Not significant	3	5.8	Single	2.5	0.5–6
*R. ravii*	Not delivered in groups	Type 1	Pulsatile, widely spaced pulses	499.2	483.1	16.1	3	4.2	Single		0.5–5
*R. sanjappai* sp. nov.	Not delivered in groups	Type 1	Pulsatile, widely spaced pulses	411.2	376.2	35.1	2	2.7	Single	2.4	0.5–3
*R. silentvalley*	Uniform intervals, not delivered in groups	Type 1	Pulsatile, widely spaced pulses	716.0	693.8	Not significant	6	7.2	Single	2.2	1–4
*R. vellikkannan* sp. nov.	Not studied										1–4
***Raorchestes flaviventris* group**		**1 type**	**Pulsatile**	**480.0–721.7**	**458.7–712.4**	**–**	**12–15**	**21.1–26.4**	**Single**	**1.7–1.9**	**1–7**
*R. flaviventris*	Uniform intervals, not delivered in groups	Type 1	Pulsatile	721.7	712.4	Not significant	15	21.1	Single	1.9	1–6
*R. ponmudi*	Uniform intervals, not delivered in groups	Type 1	Pulsatile	480.0	458.7	Not significant	12	26.4	Single	1.7	1–7
***Raorchestes glandulosus* group**		**1 type**	**Pulsatile**	**445.6–813.3**	**429.4–711.2**	**Nil–99.1**	**6–11**	**10.9–13.7**	**Single**	**2.7–3.6**	**1–8**
*R. akroparallagi*	Uniform intervals, not delivered in groups	Type 1	Pulsatile	445.6	429.4	Not significant	6	13.7	Single	3.4	1–4
*R. bobingeri*	Uniform intervals, not delivered in groups	Type 1	Pulsatile	565.6	550.1	Not significant	6	10.9	Single	3.6	1.5–7
*R. glandulosus*	Uniform intervals, not delivered in groups	Type 1	Pulsatile	609.6	588.1	Not significant	8	11.7	Single	2.7	1.5–8
*R. jayarami*	Uniform intervals, not delivered in groups	Type 1	Pulsatile	813.3	711.2	99.1	11	12.5	Single	2.9	1.5–6
***Raorchestes graminirupes* group**		**1–2 types**	**Pulsatile**	**27.8–91.3**	**Nil–50.7**	**15.9–88.7**	**3–18**	**114.8–222.5**	**Single**	**2.1–2.8**	**0–7**
*R. graminirupes*	Delivered in groups, fixed call order (Type 1 followed by Type 2)	Type 1	Pulsatile	91.3	1.7	88.7	18	222.5	Single	2.7	0–3
		Type 2	Pulsatile	27.8	1.0	26.8	5	203.3	Single	2.8	
*R. johnceei*	Delivered in groups, fixed call order (Type 1 followed by Type 2)	Type 1	Pulsatile	66.3	50.7	15.9	8	114.8	Single	2.1	0.5–7
		Type 2	Pulsatile	28.8	not significant	28.0	3	171.4	Single	2.2	
***Raorchestes nerostagona* group**		**1 type**	**Non-pulsatile**	**13.3–23.3**	**1.2–2.2**	**12.0–20.2**	**Nil**	**Nil**	**Single**	**2.0–2.2**	**3–40**
*R. crustai*	Not delivered in groups	Type 1	Non-pulsatile	13.3	1.2	12.0	1	–	Single	2.2	3–20
*R. nerostagona*	Not delivered in groups	Type 1	Non-pulsatile	23.3	2.2	20.2	1	–	Single	2.0	5–40
*R. keirasabinae* sp. nov.	Not studied										5–30
***Raorchestes signatus* group**		**1 type**	**Non-pulsatile**	**20.2**	**2.0**	**18.2**	**Nil**	**Nil**	**Single**	**2.1**	**0.5–10**
*R. signatus*	Uniform intervals, delivered in groups	Type 1	Non-pulsatile	20.2	2.0	18.2	1	–	Single	2.1	0.5–10
***Raorchestes tinniens* group**		**1 type**	**Non-pulsatile**	**8.5**	**1.1**	**6.5**	**Nil**	**Nil**	**Single**	**2.6**	**0–2**
*R. tinniens*	Uniformly intervals, delivered in groups	Type 1	Non-pulsatile	8.5	1.1	6.5	1	–	Single	2.6	0–1.5
*R. montanus*	Not studied										0–2
*R. primarrumpfi*	Not studied										0–1.5
***Raorchestes travancoricus* group**		**2 types**	**Pulsatile or Non-pulsatile**	**15.6–474.5**	**1.6–355.7**	**13.0–116.8**	**3–47**	**31.9–238.1**	**Single**	**2.2–3.4**	**0–4**
*R. agasthyaensis*	Not studied										0–2
*R. chlorosomma*	Delivered in groups, lack any fixed call order	Type 1	Pulsatile, closely packed pulses	246.2	109.5	106.6	47	195.8	Single	2.2	1.5–4
		Type 2	Pulsatile, closely packed pulses	30.2	2.0	27.3	5	238.1	Single	2.2	
*R. kadalarensis*	Delivered in groups, fixed call order (Type 1 followed by Type 2)	Type 1	Non-pulsatile	15.6	1.6	13.0	1	–	Single	3.4	0–2
		Type 2	Pulsatile	52.6	33.7	18.9	3	66.9	Single	3.4	
*R. luteolus*	Delivered in groups, fixed call order (Type 1 followed by Type 2)	Type 1	Pulsatile	390.5	270.5	88.6	12	31.9	Single	2.7	0.5–1.5
		Type 2	Non-pulsatile	27.2	1.6	25.6	1	–	Single	2.5	
*R. travancoricus*	Delivered in groups, fixed call order (Type 1 followed by Type 2)	Type 1	Pulsatile	474.5	355.7	116.8	15	33.1	Single	3.3	0.5–1.5
		Type 2	Non-pulsatile	17.2	1.8	15.3	1	–	Single	3.3	
***Raorchestes aureus* group**		**1 type**	**Pulsatile or Non-pulsatile**	**180–320**	**NA**	**NA**	**1–8**	**NA**	**Single**	**2.6–3.0**	**0.5–2**
R. aureus*	Not studied	Type 1	Non-pulsatile	180	NA	NA	1	NA	Single	3.0	0.5–2
R. lechiya*	Not studied	Type 1	Pulsatile	320	NA	NA	8 (average)	NA	Single	2.6	0.5–2
*Call properties based on data available in Zachariah et al., 2016											
**Ungrouped species**											
*R. indigo*	Not studied										0.5–3
*R. echinatus*	Not studied										0–1

**Table 3:** Distribution localities of *Raorchestes* samples examined in the present study. Localities are arranged by State.

State/District	Locality	Species studied
**Tamil Nadu**		
Coimbatore	Grass Hills	*R. dubois*, *R. flaviventris*, *R. griet*, *R. resplendens*, *R. sushili*
	Sholayar	*R. akroparallagi*, *R. anili*, *R. blandus, R ochlandrae*, *R. sushili*
	Valparai	*R. akroparallagi*, *R. beddomii*, *R. flaviventris*, *R. griet*, *R. jayarami*, *R. ochlandrae*, *R. sushili*, *R uthamani*
Dindigal Anna	Kodaikanal	*R. dubois*
Kanyakumari	Kiriparai	*R. akroparallagi*
Namakkal	Kolli Hills	*R. kollimalai*
Nilgiris	Avalanche	*R. signatus*, *R. tinniens*
	Bangitapal	*R. primarrumpfi*, *R. signatus*, *R silentvalley*
	Coonoor	*R. charius, R.coonoorensis, R. signatus, R. tinniens*
	Kotagiri	*R. coonoorensis*, *R. signatus*, *R. tinniens*
	Mukurthi	*R. lechiya*, *R. primarrumpfi*, *R. signatus*, *R. silentvalley*, *R. tinniens*
	Naduvattam	*R. charius*, *R. coonoorensis*, *R. ravii*, *R. signatus*, *R. tinniens*
	Ooty	*R. signatus*, *R. tinniens*
	Parsons Valley	*R. signatus*, *R. tinniens*
	Pykara	*R. coonoorensis*, *R. signatus*, *R. tinniens*
Salem	Yercaud	*R. kollimalai*
Theni	Bodinayakkanur	*R. travancoricus*
	Meghamalai	*R. beddomii*, *R. chlorosomma*, *R. dubois*, *R. flaviocularis*, *R griet*, *R*. cf. *kaikatti*, *R. munnarensis*, *R. travancoricus*
Tirunelveli	Kakachi	*R. agasthyaensis*, *R. bobingeri*, *R. chalazodes*, *R. crustai*, *R. graminirupes*, *R. johnceei*, *R. kakachi*, *R. manohari*
	Kannikatti	*R. akroparallagi*, *R. archeos*
	Kodayar	*R. agasthyaensis*, *R. bobingeri*, *R. chalazodes*, *R. crustai*, *R. graminirupes*, *R. johnceei*, *R. kakachi, R. manohari*
	Sengaltheri	*R. bobingeri*, *R. johnceei*
	Singampatti	*R. beddomii*
**Kerala**		
Idukki	Chinnar	*R. chlorosomma*, *R dubois*, *R griet*, *R. jayarami*, *R. kadalarensis*, *R. munnarensis*, *R. resplendens*
	Devikulam	*R. beddomii*, *R chlorosomma*, *R. dubois*, *R. griet*, *R. jayarami*, *R. kadalarensis*, *R. munnarensis*
	Eravangalar	*R. dubois*, *R. flaviocularis*, *R uthamani*
	Eravikulam National Park	*R. beddomii*, *R. chlorosomma*, *R. dubois*, *R. flaviventris*, *R griet*, *R. kadalarensis*, *R. munnarensis*, *R. ochlandrae*, *R. resplendens*, *R. sushili*
	Kozhikana	*R. akroparallagi*, *R. anili*
	Kadalar	*R. chlorosomma*, *R. dubois*, *R. drutaahu* sp. nov., *R. flaviventris*, *R. jayarami*, *R. kadalarensis*, *R. keirasabinae* sp. nov., *R. munnarensis*, *R. ochlandrae*, *R. sushili*, *R. theuerkaufi*
	Mathikettan	*R. beddomii, R. chlorosomma*, *R*. *jayarami*, *R munnarensis*, *R*. *sushili*
	Mattupetti	*R. beddomii*, *R. chlorosomma*, *R. griet*, *R. jayarami*, *R. kadalarensis*, *R. munnarensis*
	Meesapulimala	*R* dubois, *R. resplendens*
	Munnar	*R. beddomii*, *R*. cf. *bobingeri*, *kadalarensis, R. munnarensis*, *R. chlorosomma, R. griet, R. resplendens*, *R. kadalarensis*
	Painav	*R. akroparallagi*
	Thekkady	*R. anili*, *R. keirasabinae* sp. nov.
	Upper Manalar	*R. beddomii*, *R. chlorosomma*, *R. dubois*, *R. flaviocularis*, *R*. cf. *kaikatti*, *R. munnarensis*, *R. travancoricus*, *R. uthamani*
	Vandiperiyar	*R. anili*, *R. griet*, *R. keirasabinae* sp. nov., *R. ponmudi*, *R. travancoricus*
	Vagamon	*R. akroparallagi*, *R. anili*, *R*. cf. *bobingeri*, *R. griet*, *R. keirasabinae* sp. nov., *R. ponmudi*, *R. travancoricus*
	Vaguvarai	*R. beddomii*, *R chlorosomma*, *R. dubois*, *R griet*, *R munnarensis*
	Vattavada	*R. beddomii*, *R dubois*, *R. griet*
Kannur	Paithal Mala	*R. charius*, *R luteolus*, *R. tuberohumerus*
	Aralam	*R. akroparallagi*
Kasargod	Anakallu	*R. akroparallagi*
	Ranipuram	*R. anili*, *R. charius*, *R. luteolus*, *R. ponmudi*, *R. tuberohumerus*
Kollam	Shendurney	*R. agasthyaensis*, *R. akroparallagi*, *R. anili*, *R. archeos*, *R. bobingeri*, *R. beddomii*, *R. chalazodes*, *R. chotta*, *R. crustai*, *R. johnceei*, *R. kakachi*, *R. keirasabinae* sp. nov., *R. manohari*, *R. ponmudi*
	Thenmala	*R akroparallagi*
Kozhikode	Kakkayam	*R. akroparallagi*, *R. anili*, *R glandulosus*, *R. kakkayamensis* sp. nov., *R. ochlandrae*, *R. ponmudi*
Palakkad	Nelliyampathi	*R. jayarami*, *R. kaikatti*, *R. marki*, *R ochlandrae*
	Parambikulam	*R. akroparallagi*, *R. anili*, *R. blandus*, *R. ochlandrae*, *R. sushili*
	Silent Valley National Park	*R. anili*, *R charius*, *R. glandulosus*, *R. lechiya*, *R. signatus*, *R. silentvalley*, *R. tinniens, R. vellikannan* sp. nov.
	Siruvani	*R. anili*, *R. aureus*, *R. drutaahu* sp. nov., *R. leucolatus*, *R. vellikannan* sp. nov.
Pathanamthitta	Gavi	*R. akroparallagi, R. anili*, *R. keirasabinae* sp. nov, *R. ponmudi*, *R. uthamani*
	Pamba	*R. akroparallagi*
Thrissur	Chimmini	*R. kaikatti*, *R. keirasabinae* sp. nov., *R ochlandrae*
	Malakkappara–Sholayar	*R. akroparallagi*, *R. anili*, *R. blandus*, *R. ochlandrae*, *R. sushili*
	Vazhachal	*R. akroparallagi*, *R. anili*, *R. blandus*, *R. ochlandrae*
Thiruvananthapuram	Athirimala	*R. agasthyaensis*, *R. archeos*, *R. beddomii*, *R. chalazodes*, *R. crustai*, *R. graminirupes*, *R. johnceei*, *R. kakachi*, *R. manohari*
	Bonacaud	*R. akroparallagi*, *R. anili*, *R. chotta*, *R. keirasabinae* sp. nov., *R. ponmudi*
	Chathankod–Makki	*R. akroparallagi*, *R. keirasabinae* sp. nov.
	Pandipath	*R. agasthyaensis*, *R. archeos*, *R. bobingeri*, *R. beddomii*, *R. chalazodes*, *R. crustai*, *R. graminirupes*, *R. johnceei*, *R. manohari*
	Ponkalapara	*R. archeos*, *R. bobingeri*, *R. beddomii*, *R. graminirupes*
	Ponmudi	*R. akroparallagi*, *R. anili*, *R. archeos*, *R. bobingeri*, *R. chotta*, *R. graminirupes*, *R. keirasabinae* sp. nov., *R. ponmudi*
Wayanad	Banasura	*R chromasynchysi*, *R. glandulosus*, *R. ochlandrae*, *R tuberohumerus*
	Kalpetta	*R. akroparallagi*, *R. anili*, *R. nerostagona*, *R. ponmudi*, *R. tuberohumerus*
	Kurichiyarmala	*R. anili*, *R charius*, *R. chromasynchysi*, *R. glandulosus*, *R. ponmudi*
	Mananthavady	*R. akroparallagi*, *R. anili*, *R. glandulosus*, *R. nerostagona*, *R. ponmudi*, *R. tuberohumerus*
	Muthanga	*R. anili*, *R. tuberohumerus*
	Periya	*R. akroparallagi*, *R. anili*, *R. ponmudi*, *R. sanjappai* sp. nov.
	Pozhuthana	*R. akroparallagi*, *R. anili*, *R. ochlandrae*, *R. ponmudi*
	Sultan Bathery	*R. akroparallagi*, *R. anili*, *R. glandulosus*, *R. nerostagona*, *R. ponmudi*
	Thirunelly	*R. anili*, *R. charius*, *R. chromasynchysi*, *R. ochlandrae*, *R. tuberohumerus*
	Vellarimala	*R. charius*, *R. glandulosus*, *R. signatus*
	Vythiri	*R akroparallagi*, *R. anili*, *R. nerostagona*, *R. ochlandrae*, *R. ponmudi*, *R*. *tuberohumerus*
**Karnataka**		
Belgaum	Londa	*R. bombayensis*
Chamrajnagar	BR Hills	*R. honnametti*
Chikmagalur	Baba Budangiri	*R. charius*, *R. chromasynchysi*, *R. echinatus*
	Balehoonoor	*R. luteolus*, *R. tuberohumerus*
	Bygoor	*R. luteolus*, *R. tuberohumerus*
	Chikmagalur	*R. charius*, *R. tuberohumerus*, *R. luteolus*
	Kemmangundi	*R. charius*, *R. chromasynchysi*, *R. ochlandrae*
	Kudremukh NP	*R. charius*, *R. chromasynchysi*, *R. indigo*, *R. luteolus*, *R. montanus*, *R. ochlandrae*, *R. tuberohumerus*
	Mudigere	*R. luteolus*, *R. tuberohumerus*
	Muthodi	*R. charius*, *R. chromasynchysi, R. luteolus*, *R. ochlandrae*, *R. tuberohumerus*
Dakshin Kannada	Beluvai	*R. akroparallagi*
	Mangalore	*R. akroparallagi*
	Punacha	*R. akroparallagi*
Hassan	Kempholay	*R. luteolus*, *R. tuberohumerus*
	Kottigehara	*R. charius*, *R. tuberohumerus*
	Sakleshpur	*R. luteolus*, *R. tuberohumerus*
Kodagu	Bhagamandala	*R. anili*, *R. ponmudi*
	Madikeri	*R. anili*, *R. charius*, *R. glandulosus*, *R. luteolus, R. nerostagona*, *R. ponmudi*, *R. tuberohumerus*
	Nishanimotta	*R. chromasynchysi*
	Thalakaveri	*R. chromasynchysi*, *R. tuberohumerus*
	Yevakapadi	*R. anili*, *R. charius*, *R. chromasynchysi*, *R. glandulosus*, *R. luteolus*, *R. ponmudi*, *R. tuberohumerus*
Shimoga	Agumbe	*R. tuberohumerus*, *R. nerostagona*
	Jog falls	*R. luteolus, R. tuberohumerus*
Uttara Kannada	Castle Rock	*R. bombayensis*
	Mavingundi	*R. luteolus*
**Goa**		
South Goa	Netravali	*R. bombayensis*
**Maharashtra**		
Pune	Bhimashankar	*R. ghatei*
	Lavasa	*R. ghatei*
Raigad	Matheran	*R. ghatei*
	Phansad	*R. bombayensis*
Satara	Kaas	*R. ghatei*
	Mahabaleshwar	*R. ghatei*
Sawantadi	Amboli	*R. bombayensis*
**Andhra Pradesh**		
East Godavari	Maredumilli	*R. sanctisilvaticus*
Vishakhapatnam	Peddavalasa	*R. sanctisilvaticus*
	Chintapalli	*R. sanctisilvaticus*
	Borra Caves	*R. sanctisilvaticus*
	Araku Valley	*R. sanctisilvaticus*
**Odisha**		
Khurda	Barbara	*R. sanctisilvaticus*
Mayurbhanj	Similipal	*R. sanctisilvaticus*
**Madhya Pradesh**		
Anuppur	Amarkantak	*R. sanctisilvaticus*

**Etymology.** The species name is derived from Sanskrit ‘druta’ (meaning fast) and ‘ahu’ (meaning call), referring to the fast-pulsatile calls of the new species. The species epithet *drutaahu* is treated as an invariable noun in apposition to the generic name.

**Holotype.** BNHS 6088, an adult male, from Kadalar (10.1311° N, 77.0005° E, 1,430 m asl), Munnar, Idukki district, Kerala State, India, collected by SDB and SG on 17 August 2014. **Paratypes.** BNHS 6089, an adult male, collected by SDB and SG, along with the holotype; BNHS 6090, an adult male, collected by RS and SDB, from the holotype locality on 10 August 2012; and BNHS 6091, an adult male, from Siruvani (10.9587° N, 76.6667° E, 1,048 m asl), Palakkad district, Kerala State, India, collected by SDB, SG, and RS on 07 July 2015. **Referred specimen.** SDBDU 2015.3025, an adult female, from Siruvani, Palakkad district, Kerala State, India, collected by SDB, SG, and RS on 07 July 2015.

**Phylogenetic relationship.** *Raorchestes drutaahu* sp. nov. is a member of the *Raorchestes charius* group and shows a well-supported sister-group relationship with *R. coonoorensis* (Fig. 2). For the mitochondrial 16S rRNA, *Raorchestes drutaahu* is divergent from other members of the group as: 4.5–7.9% from *R. charius*; 4.3–5.2% from *R. coonoorensis*; 4.3–6.6% from *R. griet*; and 4.6–5.6% from *R. honnametti*.

**Morphological diagnosis and comparison.** *Raorchestes drutaahu* sp. nov. can be distinguished from other known congeners, except members of the *Raorchestes charius* group, by the combination of following morphological characters: a small-sized species (male SVL 20–23 mm); outline of the snout rounded to sub-ovoid in ventral view; tympanum distinct, nearly half of the eye diameter; dorsum light to dark brown or straw coloured with horny spinules and ridges; dorsum with two faint to prominent shaped concave bands, extending from behind the eye to nearly the vent; lateral surfaces of the head dark brown from tip of the snout and along the margins of the eye and supratympanic fold; a pair of black irregular shaped spots near the groin on either side of posterior dorsum; flank and groin grey or light brown without contrasting colour blotches or markings; posterior surface of thighs dark to light brown without prominent markings; iris brown with a golden tinge, horizontally divided into light upper and dark lower halves; foot webbing small, below the second subarticular tubercle on either side of toe IV (Fig. 3).

Within the *Raorchestes charius* group, *R. drutaahu* sp. nov. is more closely related to *R. charius*, *R. coonoorensis*, and *R. honnametti*. However, *R. drutaahu* sp. nov. can be differentiated from these three species by its groin with faint white blotches (vs. groin light brown with pale yellow or greyish blotches in *R. coonoorensis*; groin deep brown with yellow blotches in *R. charius*; groin light brown with minute white marbling in *R. honnametti*); a pair of black irregular shaped spots on either side of the posterior dorsum near the groin (vs. absent); and relatively reduced webbing on foot, third toe webbing just above the first subarticular tubercle on the outside (vs. more extensive, nearly up to the second subarticular tubercle). Specifically, it also differs from *R. charius* and *R. honnametti* by its relatively smaller adult size, male SVL 20–23 mm, female SVL 24.5 mm (vs. larger, male SVL 26–35 mm in *R. charius*; and male SVL 23–28 mm in *R. honnametti*). Further, it differs from *R. griet* by relatively larger adult size, male SVL 20–23 mm, female SVL 24.5 mm (vs. smaller, male SVL 18–22 mm, female SVL 22 mm); and more extensive webbing on foot, well beyond the first subarticular tubercle on either side of toe IV (vs. reduced, up to or slightly above the first subarticular tubercle on either side). *Raorchestes drutaahu* sp. nov. also differs from the most recently described species *R. kollimalai* by relatively smaller adult size, male SVL 20–23 mm, female SVL 24.5 mm (vs. larger, male SVL 25.8–29.7 mm); head nearly as wide as long (vs. wider than long); shank nearly equal or shorter than thigh (vs. longer) (Gowande, Ganesh & Mirza, 2020); and webbing between toes III and IV rudimentary, well below the first subarticular tubercle on either side (vs. nearly up to the first subarticular tubercle).

We specifically also compare *Raorchestes drutaahu* sp. nov. with the type series of *R. ravii* (see taxonomic remarks for that species), and show that it differs due to the presence of black irregular shaped spots on either side of the posterior dorsum near the groin (vs. absent); head nearly as wide as long, HW/HL ratio 0.99–1.01 (vs. wider than long, HW/HL ratio 1.35–1.36); snout rounded to sub-ovoid (vs. pointed); tympanum distinct (vs. indistinct); shank nearly equal or shorter than thigh, SHL/TL ratio 0.95–1.0 (vs. shank longer than thigh, SHL/TL ratio 1.06–1.07); relatively reduced foot webbing, fourth toe webbing below the second subarticular tubercle on either side, I1–2^+^II2^+^–3III3^–^–3^1^/_2_IV3^+^ –2^+^V (vs. more extensive, up to or above the second subarticular tubercle on either side of toe IV, I1^+^–2^+^II2^+^–3^+^III2–3^+^IV3^–^–2V; iris brown with golden tinge, horizontally divided into light upper and dark lower halves (vs. uniformly golden brown or reddish-brown); sclera light grey (vs. scarlet blue, based on the holotype photograph); and short pulsatile male advertisement calls with closely packed pulses and relatively faster pulse rate, characteristic for members of the *R. charius* group (vs. widely spaced pulses, characteristic for members of the *R. chromasynchysi* group).

**Description of holotype (*measurements in mm*).** Small-sized adult male (SVL 21.9) with a slender body; head nearly as long as wide (HL 7.8; HW 7.7; MN 6.8; MFE 5.1; MBE 2.8); outline of the snout rounded to sub-ovoid in dorsal and ventral view, rounded in lateral view; snout length (SL 3.4) longer than horizontal diameter of eye (EL 2.6); loreal region acutely flat with rounded canthus rostralis; distance between posterior margins of eyes (IBE 6.7) 1.7 times the distance between anterior margins of eyes (IFE 4.0); tympanum rather distinct (TYD 1.2), 46.2% of eye diameter (EL 2.6); supratympanic fold rather distinct; tongue with a lingual papilla. Forearm (FAL 4.9) shorter than hand (HAL 5.7); fingers without lateral dermal fringe; webbing absent; subarticular tubercles rather prominent, rounded, single, III2 and IV2 weakly-developed; prepollex rather indistinct; palmar tubercle small, rounded; supernumerary tubercles present; nuptial pad present, smooth. Hindlimbs moderately long, thigh (TL 10.5) nearly equal to shank (SHL 10.4) and longer than foot (FOL 7.9); distance from heel to tip of toe IV (TFOL 14.0); foot webbing small: I1–2^+^II2^+^–3III3^–^–3^1^/_2_IV3^+^–2^+^V, below the second subarticular tubercle on either side of toe IV; dermal fringe along toe V absent; subarticular tubercles rather prominent, rounded, simple, IV2 and V2 weakly-developed; supernumerary tubercles present (Fig. 3).

Skin of snout and between eyes shagreened with fine scattered and various sized granular projections; a faint horny ridge extending from the tip of the snout to the vent; a weakly-developed horny ridge between the eyes, arranged in a triangle directed posteriorly; lateral surfaces of head shagreened; dorsal surface of limbs shagreened to sparsely granular. Ventral skin on throat shagreened to granular; chest, belly, and posterior surface of thighs granular.

**Colour of holotype.** *In life*. Dorsum brown; a faint dark grey stripe between the eyes; dorsum with two dark brown )( shaped concave bands, extending from behind the eyes to the level of the groin; a pair of black irregular shaped spots on either side of the posterior dorsum near the groin; lateral surfaces of head dark brown; lateral abdominal surfaces lighter than dorsum; groin light greyish-brown without blotches; anterior and posterior surface of thighs brown with dark greyish-brown mottling; dark blackish-brown markings around the cloacal opening; fore and hind limbs (including fingers and toes) brown or light brown with a few scattered dark brown cross-bands; iris brown with golden tinge, upper half lighter than lower half. Ventral surfaces light brown with minute dark brown speckling; hand and foot greyish-brown (Fig. 3). *In preservation*. Dorsum dark grey with blackish-brown )( shaped concave bands; lateral surfaces of head blackish-brown; lateral abdominal surfaces greyish with small off-white spots; groin grey with light grey blotches; posterior surface of thighs light brown with scattered creamish mottling; limbs with dark cross-bands. Ventral surface of throat creamish-white with dense minute dark brown speckles; chest, belly, fore and hind limbs creamish-white with scattered dark brown speckles; limbs greyish-brown with creamish-white mottling (Fig. 3).

**Variations.** Morphometric data from five specimens, including the holotype, is given in Table S2. The dorsal colouration and markings are variable in life: BNHS 6089 and BNHS 6090: dark blackish-brown markings around the cloacal opening, surrounded with white patches; BNHS 6091: dorsum greyish-brown with prominent dark brown markings; stripe between eyes dark brown. BNHS 6089 and SDBDU 2015.3025: dorsum light greyish-brown to straw coloured with faint and irregular dark brown dorsal markings.

**Vocalisation.** *Raorchestes drutaahu* sp. nov. males produce a single type of call. Calls are not delivered in groups and have a pulsatile temporal structure, with relatively short and closely packed pulses. A typical call shows a duration of 50.6 ms; the amplitude envelope being characterised by a rise time of 1.2 ms and fall time of 49.1 ms; with six pulses delivered at a rate of 134.5 pulses/second; and the spectrum showing a single broad peak with mean dominant frequency of 3.6 kHz. For comparison see Table 2 and the group definition, including the oscillogram and spectrogram figures cited therein.

**Distribution and natural history.** *Raorchestes drutaahu* sp. nov. is endemic to the Western Ghats and currently known only from elevations ranging between 1,000 to 1,450 m asl at two localities: Kadalar in Idukki district (south of Palghat gap) and Siruvani in Palakkad district (north of Palghat gap). The species has been observed in forest areas, either on grassland-shola fringes or fragmented forest patches near plantations. Individuals were located on leaves of short shrubs at heights of 0.5–1.5 m.

***Raorchestes kakkayamensis*** sp. nov.

http://zoobank.org/urn:lsid:zoobank.org:act:BD7A17C6-A1E2-421A-B0E8-2336931CD63F

**Figure 4:**
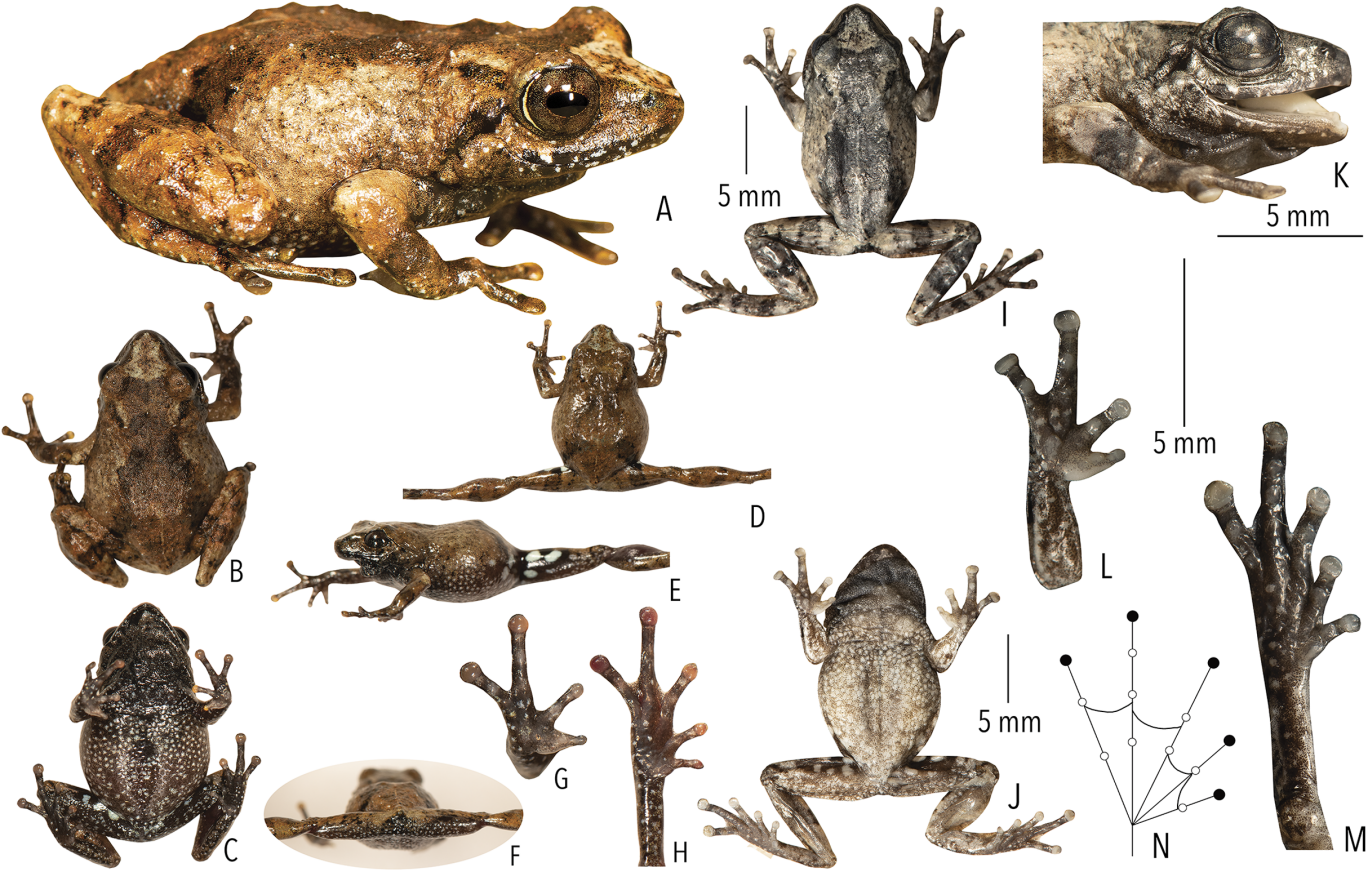
Holotype (BNHS 6092, adult male) of *Raorchestes kakkayamensis* sp. nov. (A–H) In life. (A) Dorsolateral view. (B) Dorsal view. (C) Ventral view. (D) Dorsal view of body and thighs. (E) Lateral view. (F) Posterior view of thighs. (G) Ventral view of hand. (H) Ventral view of foot. (I–N) In preservation. (I) Dorsal view. (J) Ventral view. (K) Lateral view of head. (L) Ventral view of hand. (M) Ventral view of foot. (N) Schematic illustration of webbing on foot.

**Figure 5:**
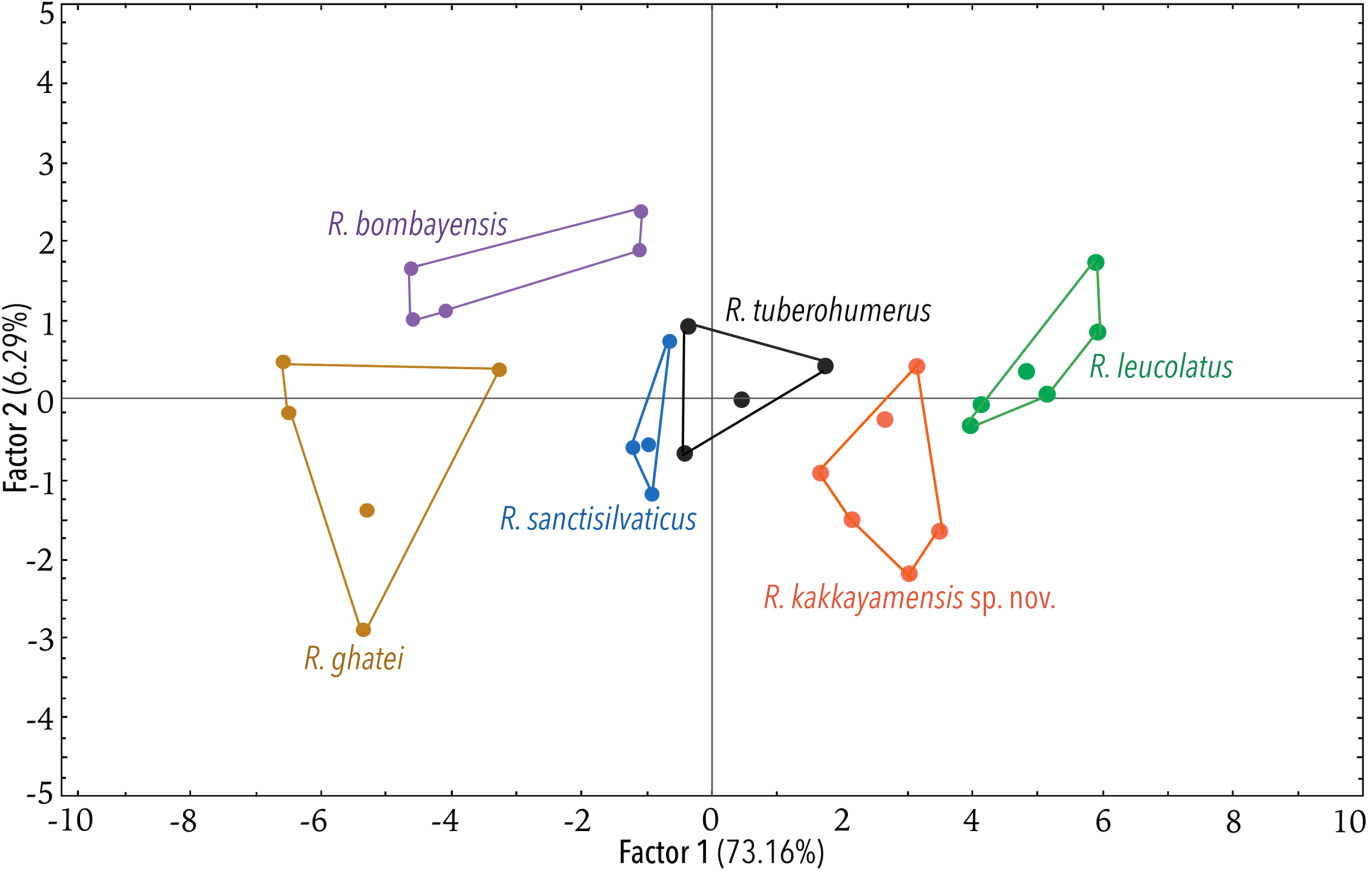
Projection of the first two Principal Component (PC) factor planes explaining 79.45% of the total variation among six species of the *Raorchestes bombayensis* group and showing morphometric distinctness of the new species, *R. kakkayamensis*.

Kakkayam Shrub Frog

(Figs. 2, 4 and 5; Tables 1–3; Tables S1–S5)

**Etymology.** The species is named after the place Kakkayam, where the type series was collected.

**Holotype.** BNHS 6092, an adult male, from Kakkayam (11.5542° N, 75.9196° E, 750 m asl), Kozhikode district, Kerala State, India, collected by SDB and RS in June 2018. **Paratypes.** BNHS 6093–6096, four adult males, collected along with holotype. **Referred specimen.** SDBDU 2019.3423, an adult male, from the holotype locality, collected by RS and SD on 28 July 2018.

**Phylogenetic relationship.** *Raorchestes kakkayamensis* sp. nov. is a member of the *Raorchestes bombayensis* group and shows a well-supported sister-group relationship with *R. leucolatus* (Fig. 2). For the mitochondrial 16S rRNA, *Raorchestes kakkayamensis* is divergent from other members of the group as: 3.2–4.6% from *R. bombayensis*; 5.3–5.9% from *R. ghatei*; 2.3–2.4% from *R. leucolatus*; 4.0–4.5% from *R. sanctisilvaticus*; and 3.1–3.6% from *R. tuberohumerus*.

**Morphological diagnosis and comparison.** *Raorchestes kakkayamensis* sp. nov. can be distinguished from other known congeners, except members of the *Raorchestes bombayensis* group, by the combination of the following morphological characters: small-sized species (male SVL 17–19 mm, female SVL 24 mm); a knobbed bony projection on humerus in males (visible externally in preserved specimens); lateral surfaces of abdomen and groin with contrasting white blotches on grey to dark grey background; presence of horny spinules and horny ridges on dorsal skin; and finger and toe discs yellowish-brown (Fig. 4).

Within the *Raorchestes bombayensis* group, *Raorchestes kakkayamensis* sp. nov. differs from *R. bombayensis* and *R. ghatei* by its smaller adult size, male SVL 17–19 mm (vs. male SVL > 19 mm); differs from *R. tuberohumerus* by its relatively smaller adult size, male SVL 17–19 mm (vs. larger, male SVL 18–22 mm); flank and groin with white blotches on grey to dark grey background (vs. yellow blotches); and finger and toe discs yellowish-brown (vs. grey to brown); and differs from *R. leucolatus* by its head longer than wide, HW/HL ratio 0.86–0.95 (vs. wider than long, HW/HL ratio 1.20–1.33); thigh relatively longer than shank, TL/SHL ratio 1.03–1.12 (vs. nearly equal, TL/SHL ratio 0.99–1.04); supernumerary tubercles prominent on toes II–V (vs. weakly-developed); and finger and toe discs yellowish-brown (vs. orange or orangish-red).

Morphometrically, the new species *Raorchestes kakkayamensis* was also well differentiated from the other recognised members of the *Raorchestes bombayensis* group, that is *R. bombayensis*, *R. ghatei*, *R. leucolatus*, *R. sanctisilvaticus*, and *R. tuberohumerus*. All the six species formed distinct clusters when projected on the first two PCA factor planes that had eigenvalues >1.0 and explained 79.44% of variation among the species (Fig. 5). The PCA factor loadings representing the composition of PCA factors, and the parameters correlated with each PCA factor are shown in Table S3. PCA Factor 1 loaded heavily on all the morphometric parameters (total 20) except FW_III_, while PCA Factor 2 loaded heavily on two morphometric parameters, FW_III_ and TW_IV_. Furthermore, our DFA resulted in 100% classification success with all the individual samples being classified into their respective species (Table S5). The coefficients of canonical discriminant function representing the composition of standardized canonical discriminant scores are shown in Table S4. All the five discriminant function roots showed eigenvalues >1.0 and explained 100% of the variations among these species. The PCA and DFA results provide additional evidence for morphometric differentiation of *Raorchestes kakkayamensis* sp. nov. from its closest congeners.

**Description of holotype (*measurements in mm*).** Small-sized adult male (SVL 18.8) with a slender body; head longer than wide (HL 7.0; HW 6.5; MN 5.0; MFE 5.1; MBE 1.9); outline of the snout nearly sub-ovoid in dorsal and ventral view, acute in lateral view; snout length (SL 2.8) longer than horizontal diameter of eye (EL 2.5); loreal region acute with indistinct canthus rostralis; distance between posterior margins of eyes (IBE 6.1) twice the distance between anterior margins of eyes (IFE 3.1); tympanum (TYD 1.0) 40% of eye diameter (EL 2.5); supratympanic fold rather indistinct; tongue with a lingual papilla. Forearm (FAL 3.9) shorter than hand (HAL 4.9); fingers without lateral dermal fringe; webbing absent; subarticular tubercles rather prominent, rounded, single, III2 and IV2 weakly-developed; prepollex rather indistinct; palmar tubercle small, rounded; supernumerary tubercles absent; nuptial pad present, smooth. Hindlimbs moderately long, thigh (TL 9.2) longer than shank (SHL 8.9) and foot (FOL 7.3); distance from heel to tip of toe IV (TFOL 11.8); foot webbing small: I2–2II2–3III2^1^/_4_–3^1^/_2_IV3^+^–2V, below the second subarticular tubercle on either side of toe IV; dermal fringe along toe V absent; subarticular tubercles rather prominent, rounded, simple, IV2 and V2 weakly-developed; supernumerary tubercles absent (Fig. 4).

Skin of snout and between eyes shagreened; upper eyelids and lateral surfaces of head shagreened to sparsely glandular; anterior and posterior part of back shagreened with sparsely scattered spinular projections; lateral surfaces of abdomen with scattered granulations. Ventral surface of throat and chest shagreened to granular; belly and posterior surface of thighs granular (Fig. 4).

**Colour of holotype.** *In life*. Dorsum brown to reddish-brown with a pair of dark discontinuous concave bands extending from behind the eye to the level of the groin, with a thin mid-dorsal line; a dark brown coloured horizontal band between the upper eyelids; snout lighter in colour than the dorsum; lateral surfaces of head darker than dorsal colouration; lateral abdominal surfaces light brown with small off-white spots; groin and anterior surface of thighs dark brown with distinct white blotches; posterior surface of thighs light and dark brown with scattered white spots; limbs with dark brown cross-bands; finger and toe discs yellowish-brown; iris brown with dense golden yellow speckling and faint dark brown horizontal and vertical bands. Ventral surface of throat blackish-brown with scattered white speckles; chest, and belly, light brown with dense dark brown speckling and irregular white spots; fore and hind limbs dark brown with white to ash blue spots and minute light brown speckling (Fig. 4). *In preservation*. Dorsum greyish-brown with blackish-brown discontinuous concave bands extending from behind the eye to the level of the groin, with a thin light grey mid-dorsal line; snout light grey; lateral surfaces of head darker than dorsal colouration; lateral abdominal surfaces greyish-brown with small off-white spots; groin and anterior surface of thighs dark brown with distinct white blotches; posterior surface of thighs light and greyish-brown with scattered white spots; limbs with faint or prominent dark greyish-brown cross-bands. Ventral surface of throat dark brown with scattered white speckles, especially along the margins of lower jaw; chest, belly, fore and hind limbs light brown with white spots and minute dark brown speckling (Fig. 4).

**Variations.** Morphometric data from six specimens, including the holotype, is given in Table S2. The dorsal colouration and markings are slightly variable from light to dark brown or reddish-brown, but there is a uniform triangular light brown colouration from the snout tip to the anterior margins of the eyes. BNHS 6096: less prominent dorsal markings and granulations.

**Vocalisation.** *Raorchestes kakkayamensis* sp. nov. males produce a single type of call. The calls generally have a single pulse and are not delivered in groups. A typical call has duration of 25.3 ms, the amplitude envelope being characterised by a rise time of 9.2 ms and fall time of 15.9 ms, with a dominant frequency of 3.8 kHz. For comparison see Table 2 and the group definition, including the oscillogram and spectrogram figures cited therein.

**Distribution and natural history.** *Raorchestes kakkayamensis* sp. nov. is endemic to the Western Ghats and currently known only from its type locality (Kakkayam) in Kozhikode district, north of Palghat gap in Kerala State. The species was observed inside a primary forest patch and adjoining secondary forest areas at an elevation of 750 m asl. Individuals were located on the ground leaf litter or found perching on vegetation 1–3 m high.

***Raorchestes keirasabinae*** sp. nov.

http://zoobank.org/urn:lsid:zoobank.org:act:39BCB0CE-166E-4C90-AE1E-564BD626ABA9

**Figure 6:**
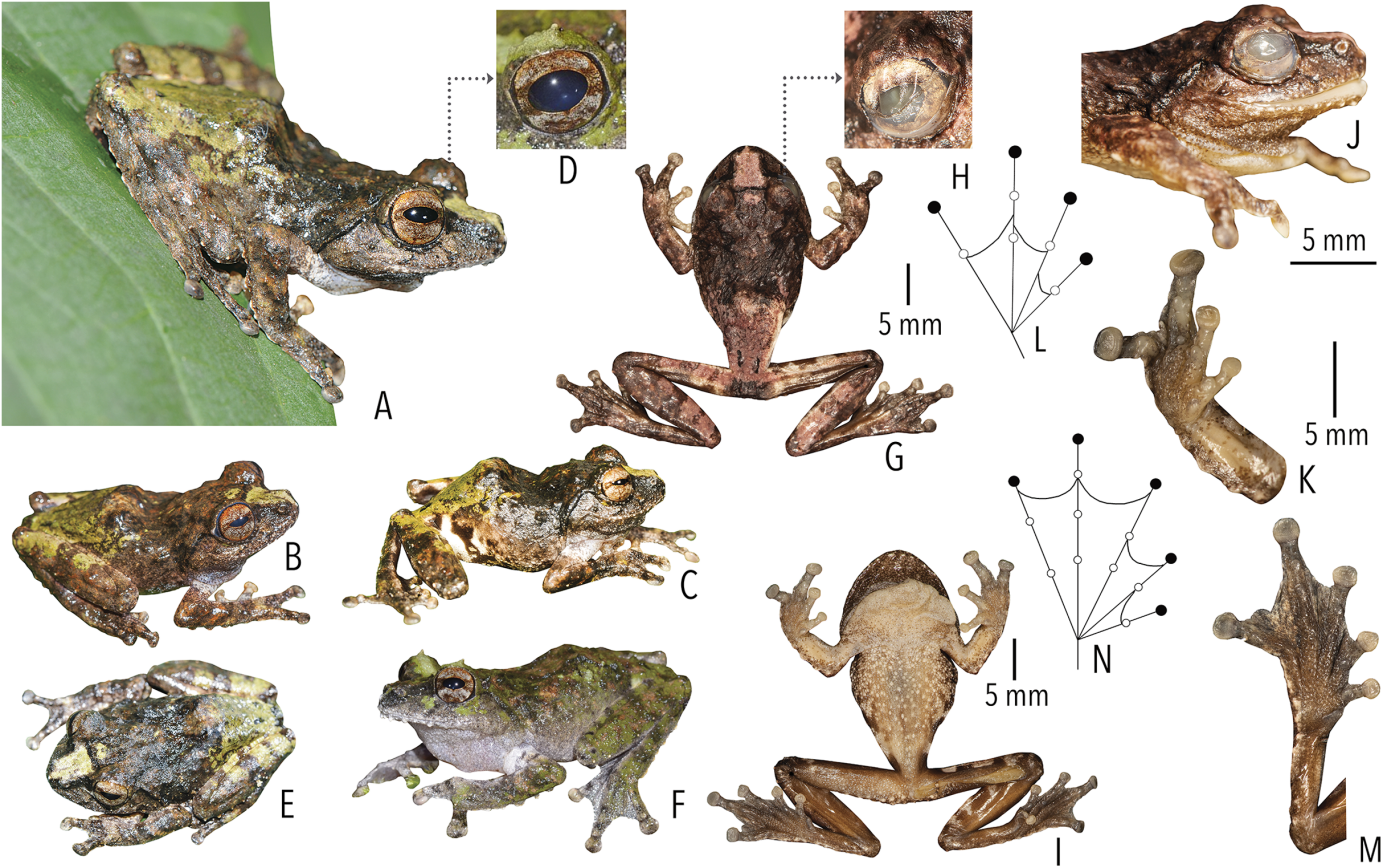
*Raorchestes keirasabinae* sp. nov. (A–F) In life. (A–C) Holotype (BNHS 6097, adult male), dorsolateral view. (D) Enlarged view of spinular projections on the upper eyelid of the holotype. (E) Dorsal view (not collected). (F) Frontolateral view (not collected). (G–N) Holotype (BNHS 6097, adult male), in preservation. (G) Dorsal view. (H) Enlarged view of spinular projections on the upper eyelid. (I) Ventral view. (J) Lateral view of head. (K) Ventral view of hand. (L) Schematic illustration of webbing on hand. (M) Ventral view of foot. (N) Schematic illustration of webbing on foot.

Keira’s Shrub Frog

(Figs. 2 and 6; Tables 1–3; Tables S1 and S2)

**Etymology.** The species is named after a young nature lover Keira Sabin, in appreciation of the long-time support and commitment of the Andrew Sabin Family Foundation towards amphibian research and conservation around the world. The species epithet *keirasabinae* is treated as a noun in the genitive case.

**Holotype.** BNHS 6097, an adult male, from Chathankod-Makki (8.6723° N, 77.1301° E, 230 m asl), Thiruvananthapuram district, Kerala State, India, collected by SDB and SG on 29 April 2015. **Paratype.** BNHS 6098, an adult male, from Ponmudi (8.75° N, 77.13° E, 980 m asl), Thiruvananthapuram district, Kerala State, India, collected by SDB in May 2006. **Referred specimen.** SDBDU 2019.3450, an adult male, from Vallakadavu (9.5281° N, 77.1144° E, 834 m asl), Periyar Tiger Reserve, Idukki district, Kerala State, India, collected by SD on 27 July 2016.

**Phylogenetic relationship.** *Raorchestes keirasabinae* sp. nov. is a member of the *Raorchestes nerostagona* group and shows a well-supported sister-group relationship with *R. nerostagona* (Fig. 2). For the mitochondrial 16S rRNA, *Raorchestes keirasabinae* sp. nov. is divergent from other members of the group as: 6.1–6.6% from *R. crustai* and 3.1–3.7% from *R. nerostagona*.

**Morphological diagnosis and comparison.** *Raorchestes keirasabinae* sp. nov. can be distinguished from other known congeners, except members of the *Raorchestes nerostagona* group, by the combination of the following morphological characters: medium-sized (male SVL 29–31 mm) predominantly canopy dwelling species; snout vertical in lateral view; webbed fingers; nearly fully webbed toes; and a distinct dermal fringe along the outer margins of the fore and hind limbs (Fig. 6). Further, its distribution is reportedly restricted to regions south of Palghat gap in the Western Ghats, whereas its sister species *R. nerostagona* is found north of Palghat gap.

Within the *Raorchestes nerostagona* group, *Raorchestes keirasabinae* sp. nov. differs from the closely related *R. nerostagona* by relatively reduced webbing between fingers, I1–2^+^II2–3^−^III2^1^/_2_–2IV (vs. more extensive, I1– 2^+^II2–2^1^/_2_III2–2^−^IV) as well as toes, I1–2II1^+^–2III1^+^–2IV2–1^+^V (vs. more extensive, I1–2II1–2^−^III1–2^−^IV2–1V); and its snout length nearly equal to the eye diameter, SL/EL ratio 1.0–1.02 (vs. snout relatively longer than eye, SL/EL ratio 1.32–1.47). Further, *Raorchestes keirasabinae* sp. nov. differs from *R. crustai* by the outline of its snout nearly rounded in dorsal and ventral view (vs. nearly pointed), vertical in lateral view (vs. obtuse); well-developed dermal fringe along the outer margins of the fore and hind limbs (vs. weakly-developed); nearly fully webbed toes, I1–2II1^+^–2III1^+^–2IV2–1^+^V (vs. relatively reduced webbing between toes, I1–2^+^II2–3^−^III2–3IV2–1^2^/_3_V); and dorsal skin granular with prominent spinular projections on lateral surfaces of head, between eyes, on upper eyelids, and on dorsum and flanks (vs. dorsal skin granular with a few spinular projections on upper eyelids).

**Description of holotype (*measurements in mm*).** Medium-sized adult male (SVL 29.6) with a slender body; head wider than long (HL 11.3; HW 12.1; MN 9.7; MFE 7.8; MBE 4.1); outline of the snout rounded in dorsal and ventral view, vertical in lateral view; snout length (SL 4.5) nearly equal to the horizontal diameter of eye (EL 4.4); loreal region obtusely concave, canthus rostralis sharp; tympanum (TYD 1.4) 31.8% of eye diameter (EL 4.4); supratympanic fold rather indistinct; tongue emarginate with a lingual papilla. Forearm (FAL 5.8) shorter than hand (HAL 8.9); fingers without prominent lateral dermal fringe; webbing present, I1–2^+^II2–3^–^III2^1^/_2_–2IV; subarticular tubercles rather prominent, rounded, III1 and IV1 double, III2 and IV2 weakly-developed; prepollex rather distinct and oval; supernumerary tubercles present. Hindlimbs moderately long, thigh (TL 15.7) longer than shank (SHL 14.5) and foot (FOL 12.9); distance from heel to tip of toe IV (TFOL 20.3); foot webbing: I1–2II1^+^–2III1^+^–2IV2–1^+^V; dermal fringe along toe V present with serrated margins, ending with a well-developed spinular projection on the heel; subarticular tubercles rather prominent, rounded, simple, IV1 and V1 weakly-developed; supernumerary tubercles absent, foot ventral side granular (Fig. 6).

Dorsal skin on snout shagreened to granular; lateral surfaces of head, between eyes, and upper eyelids, glandular with short spinular projections; anterior and posterior parts of dorsum prominently glandular with spinular projections; dorsal surfaces of fore and hind limbs shagreened with some scattered granules; a distinct dermal fringe along the outer margin of the fore and hind limbs, ending with a well-developed spinular projection on the heel and elbow. Ventral surfaces of throat, chest, belly, and posterior part of thighs glandular (Fig. 6).

**Colour of holotype.** *In life*. Anterior part of dorsum brown with black and dark brown patches, posterior half green with grey patches; snout light green dorsally; lateral surfaces of head dark greyish-brown; dorsal surface of fore and hind limbs light greyish-brown with irregular dark grey and light green cross-bands; lateral surfaces of belly with brown and grey mottling; groin and anterior parts of thigh brown with white patches; posterior margins of thigh and shank chocolate dark brown; hand and foot brownish-grey with green tinge; iris reddish-grey with faint horizontal brown band. Ventral surfaces off-white, with variable amounts of brown or grey spots forming a vermiculated pattern; throat darker compared to the belly, with dark grey margins and bands along the lips (Fig. 6). *In preservation*. Anterior part of dorsum dark greyish-brown; posterior half light brown with light pink tinge; snout light pinkish-grey; lateral surfaces of head light brown; dorsal surface of fore and hind limbs light pinkish-brown with dark brown cross-bands; lateral surfaces of belly light brown vermiculated with cream white; groin brown with white patches; posterior margins of thigh and shank brown. Ventral surfaces light greyish-brown with white and brown spots; throat darker brown compared to the belly, with dark brown margins and bands along the lips (Fig. 6).

**Variations.** Morphometric data from three specimens, including the holotype, is given in Table S2. This species can have variable dorsal colour and markings that can be adapted according to its surroundings, possibly helping in camouflage. Spinular projections on the dorsal surfaces and flanks are more prominent in life.

**Distribution and natural history.** *Raorchestes keirasabinae* sp. nov. is endemic to the Western Ghats and currently known from elevations of 100–1,000 m asl south of Palghat gap. It has been observed at Agasthyamalai Biosphere Reserve (Chathankod-Makki, Ponmudi, Peppara Wildlife Sanctuary, and Shendurney Wildlife Sanctuary) in Thiruvananthapuram and Kollam districts, and Periyar Tiger Reserve in Idukki district of Kerala State. Since the species inhabits the highest canopy layers and cannot be located easily, it could have a wider geographical range in the Western Ghats regions south of Palghat gap, both in Kerala and the adjoining State of Tamil Nadu. The vocalisations of this species have not been recorded and analysed.

***Raorchestes sanjappai*** sp. nov.

http://zoobank.org/urn:lsid:zoobank.org:act:924C6AC3-6407-4CB5-B00C-0619C6645D97

**Figure 7:**
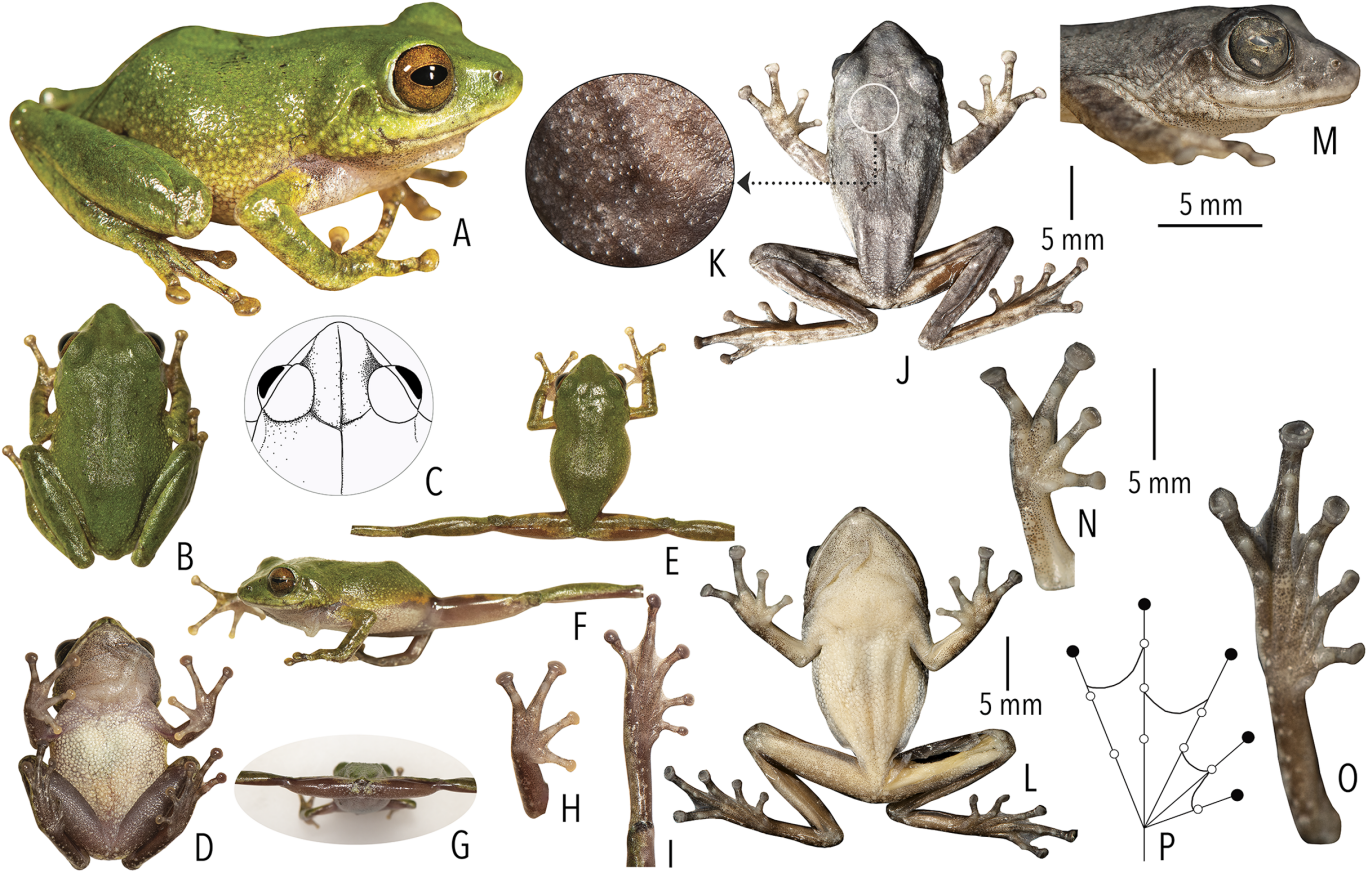
Holotype (BNHS 6099, adult male) of *Raorchestes sanjappai* sp. nov. (A–I) In life. (A) Dorsolateral view. (B) Dorsal view. (C) Illustration of the mid-dorsal horny ridge extending from the snout tip to the vent. (D) Ventral view. (E) Dorsal view of body and thighs. (F) Lateral view. (G) Posterior view of thighs. (H) Ventral view of hand. (I) Ventral view of foot. (J–P) In preservation. (J) Dorsal view. (K) Enlarged view of horny spinules on the dorsal skin. (L) Ventral view. (M) Lateral view of head. (N) Ventral view of hand. (O) Ventral view of foot. (P) Schematic illustration of webbing on foot.

Sanjappa’s Shrub Frog

(Figs. 2 and 7; Tables 1–3; Tables S1 and S2)

**Etymology.** The species is named after Dr. M. Sanjappa, a renowned Indian Botanist and former Director of the Botanical Survey of India. The species name is in appreciation of his taxonomic contributions as well as generous support to SDB during the initial phases of his research career. The species epithet *sanjappai* is treated as a noun in the genitive case.

**Holotype.** BNHS 6099, an adult male, from Periya (11.8342° N, 75.8574° E, 750 m asl), Wayanad district, Kerala State, India, collected by RS and SDB on 10 June 2015. **Paratype.** BNHS 6100, an adult male, from the holotype locality, collected by RS and SDB on 29 July 2013. **Referred specimen.** SDBDU 2019.3440, an adult male, collected by RS and SD, from the holotype locality on 30 July 2018.

**Phylogenetic relationship.** *Raorchestes sanjappai* sp. nov. is a member of the *Raorchestes chromasynchysi* group and shows a sister-group relationship with *R. vellikkannan* sp. nov. (Fig. 2). For the mitochondrial 16S rRNA, *Raorchestes sanjappai* is divergent from other members of the group as: 4.6–5.5% from *R. chromasynchysi*; 5.9% from *R. ravii*; 5.1–5.7% from *R. silentvalley*; and 3.1–3.3% from *R. vellikkannan* sp. nov.

**Morphological diagnosis and comparison.** *Raorchestes sanjappai* sp. nov. can be distinguished from other known congeners, except members of the *Raorchestes chromasynchysi* group, by the combination of the following morphological characters: small-sized species (male SVL 22–24 mm); uniform green dorsal colouration; dorsal skin shagreened to sparsely granular with horny spinular projections and a horny ridge extending from the snout tip to the vent; tongue with papillae; flanks and groin light brown to yellowish; and foot webbing moderate, below the third subarticular tubercle on either side of toe IV (Fig. 7).

Within the *Raorchestes chromasynchysi* group, *Raorchestes sanjappai* sp. nov. differs from *R. chromasynchysi* in having a rather indistinct tympanum (vs. distinct); head wider than long, HW/HL ratio 1.10–1.12 (vs. nearly equal, HW/HL ratio 0.97–1.06); snout longer than eye diameter, SL/EL ratio 1.17–1.42 (vs. nearly equal, SL/EL ratio 0.94–1.03); and relatively more extensive webbing on foot, extending up to the third subarticular tubercle on the outside of toe IV, I2–2II2–3III2^–^–3IV2–2^–^V (vs. reduced, just above the second subarticular tubercle, I1^+^–2^+^II2–3III2^–^–3^–^IV3^–^–2^–^V). It differs from *R. vellikkannan* sp. nov. by head wider than long, HW/HL ratio 1.10–1.12 (vs. nearly equal, HW/HL ratio 0.99–1.0); foot webbing moderate, extending up to the third subarticular tubercle on the outer side of toe IV, I2–2II2–3III2^–^–3IV2–2^–^V (vs. relatively reduced, below the second subarticular tubercle on either side of toe IV, I2–2^+^II2–3III2–3^1^/_3_IV3^1^/_2_–2^+^V); and dorsal skin shagreened to sparsely granular with relatively less prominent spinules (vs. prominently granular with more sharply pointed spinules). It differs from *R. silentvalley* by relatively reduced webbing on foot, not extending beyond the third subarticular tubercle on either side of toe IV, I2–2II2–3III2^–^–3IV2–2^–^V (vs. more extensive, above the third subarticular tubercle on either side of toe IV, I1^+^–2^+^II2–3III1^+^–2^–^IV2^–^–1^+^V); lateral surfaces of abdomen and groin light brown to yellowish (vs. lateral surfaces of abdomen bright yellow and groin dark purplish-blue; and ventral surfaces of palm and feet, including the digit tips, light grey (vs. purplish-black). It differs from *R. ravii* by snout sub-ovoid in in ventral view (vs. pointed); head wider than long, HW/HL ratio 1.10–1.12 mm (vs. nearly equal, HW/HL ratio 0.99–1.01); thigh nearly equal to shank, TL/SHL 0.98 (vs. relatively shorter, TL/SHL 0.93–0.95); and relatively more extensive webbing on foot, outer side of IV toe webbing reach up to third subarticular tubercle, I2–2II2–3III2^–^–3IV2–2^–^V (vs. reduced, just above the second subarticular tubercle, I2–2II2–3III2^–^–3^1^/_2_IV3^–^–2^–^V).

**Description of holotype (*measurements in mm*).** Small-sized adult male (SVL 23.9) with a slender body; head wider than long (HW 9.1; HL 8.6; MN 7.5; MFE 6.0; MBE 3.2); the outline of snout sub-ovoid in dorsal and ventral views, rounded in lateral view; snout length (SL 3.7) longer than horizontal diameter of eye (EL 2.6); loreal region acute with rounded canthus rostralis; tympanum rather indistinct; supratympanic fold rather distinct; tongue emarginate with a lingual papilla. Forearm (FAL 5.0) shorter than hand (HAL 6.8); fingers without lateral dermal fringe; webbing absent; subarticular tubercles rather prominent, rounded, III1 and IV1 weakly-developed; prepollex rather distinct and oval; supernumerary tubercles absent. Hindlimbs moderately long, thigh (TL 12.0) shorter than shank (SHL 12.2) and longer than foot (FOL 9.0); distance from heel to tip of toe IV (TFOL 15.1); foot webbing moderate: I2–2II2–3III2^–^–3IV2–2^–^V; dermal fringe absent; subarticular tubercles rather prominent, rounded, simple, IV1 and V1 weakly-developed; supernumerary tubercles absent (Fig. 7).

Dorsal skin shagreened to sparsely granular with horny spinules; a mid-dorsal horny ridge extending from the snout tip to the vent; lateral surfaces of head relatively more granular in comparison to the dorsum; lateral abdominal surfaces relatively less granular compared to the dorsum; limbs shagreened to sparsely granular. Ventral skin on throat shagreened to sparsely granular; chest, belly and posterior surface of thighs granular; limbs shagreened (Fig. 7).

**Colour of holotype.** *In life*. Dorsum uniformly green, without prominent dorsal markings; dorsal colouration extends onto the dorsal surface of fore and hind limbs, and loreal and tympanic regions; lateral abdomen surfaces light brown to yellowish; groin light brown with yellow markings (but not blotches); posterior part of thighs light to dark brown, with light greenish spots on the anal region; iris reddish-brown. Ventral surface of throat light flesh grey with minute dark spots; chest and belly greyish-white; limbs greyish-brown; hand and foot dark grey or blackish (Fig. 7). *In preservation*. Dorsum bluish-grey without prominent dorsal markings; lateral abdomen surfaces light grey; groin light brown; posterior part of thighs dark brown. Ventral surfaces off-white without prominent markings, other than minute black spots on throat; limbs off-white to greyish-brown with darker spots towards the margins (Fig. 7).

**Variations.** Morphometric data from three specimens, including the holotype, are given in Table S2.

**Vocalisation.** *Raorchestes sanjappai* sp. nov. males produce a single type of call. Calls are not delivered in groups and have a pulsatile temporal structure with widely spaced pulses. A typical call has a duration of 411.2 ms, the call envelope being characterised by a rise time of 376.2 ms and fall time of 35.1 ms, with two pulses delivered at a rate of 2.7 pulses/second, and an overall dominant frequency of 2.4 kHz. For comparison see Table 2 and the group definition, including the oscillogram and spectrogram figures cited therein.

**Distribution and natural history.** *Raorchestes sanjappai* sp. nov. is endemic to the Western Ghats and currently known only from an altitude of about 750 m asl at its type locality (Periya) that lies north of Palghat gap in the Wayanad district of Kerala State. The species was observed inside secondary forests and individuals were located on vegetation up to 3 m high during the breeding season.

***Raorchestes vellikkannan*** sp. nov.

http://zoobank.org/urn:lsid:zoobank.org:act:3E3E9E2A-E489-4AAD-8975-275BF0008CCB

**Figure 8:**
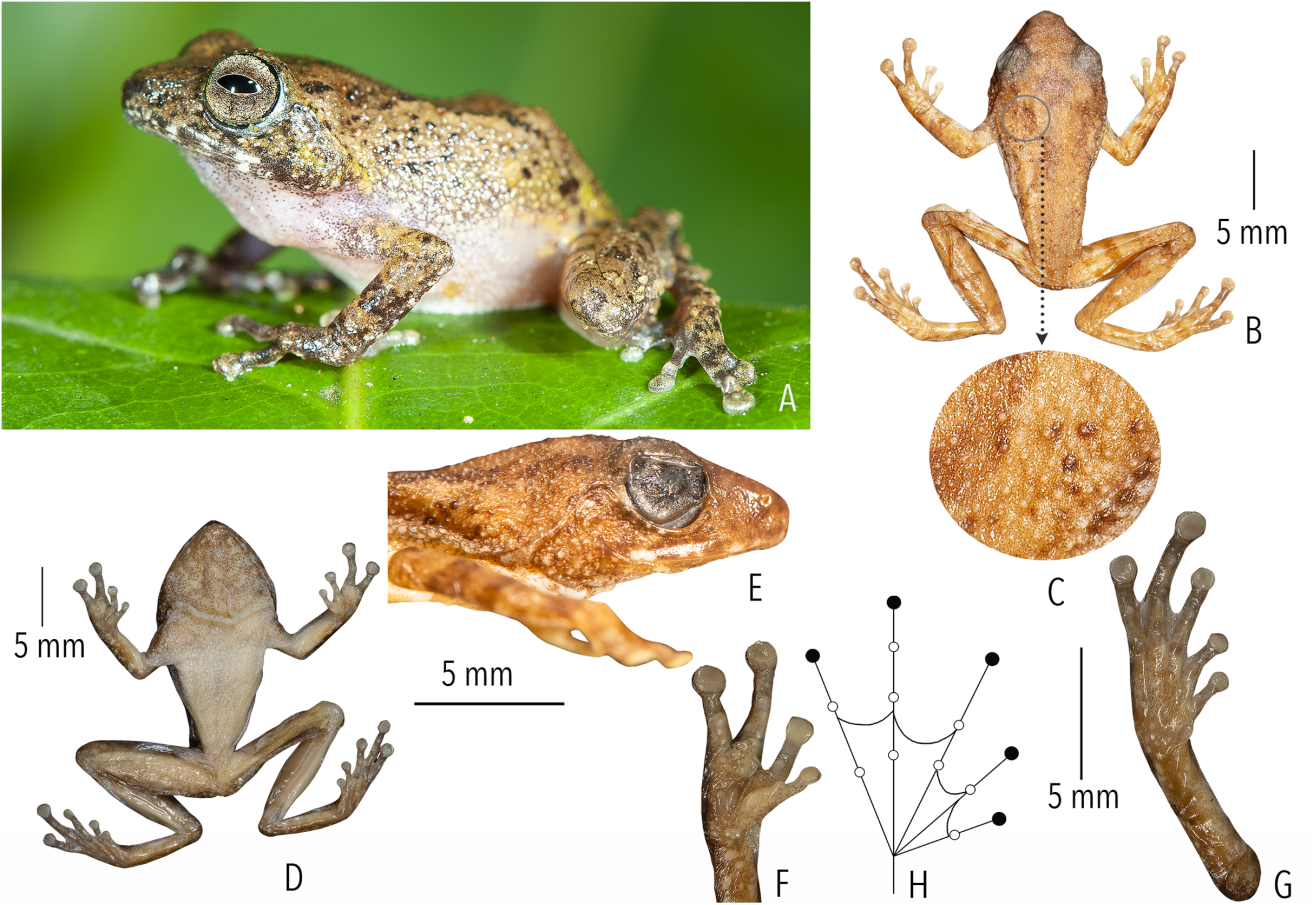
Holotype (BNHS 6101, adult male) of *Raorchestes vellikkannan* sp. nov. (A) Dorsolateral view in life. (B–H) In preservation. (B) Dorsal view. (C) Enlarged view of the dorsal skin texture. (D) Ventral view. (E) Lateral view of head. (F) Ventral view of hand. (G) Ventral view of foot. (H) Schematic illustration of webbing on foot.

Silver-eyed Shrub Frog

(Figs. 2 and 8; Tables 1–3; Tables S1 and S2)

**Etymology.** The species name is derived from Malayalam (the language of Kerala State where the type series were collected) ‘velli’ (meaning silver) and ‘kannu’ (meaning eye) referring to the silver colour of the iris in this species. The species epithet *vellikkannan* is treated as an invariable noun in apposition to the generic name.

**Holotype.** BNHS 6101, an adult male, from Singappara (10.9794° N, 76.615° E, 856 m asl), Siruvani, Palakkad district, Kerala State, India, collected by SDB, SG, and RS on 06 July 2015. **Referred specimen.** SDBDU 2015.3019, an adult male, collected along with the holotype.

**Phylogenetic relationship.** *Raorchestes vellikkannan* sp. nov. is a member of the *Raorchestes chromasynchysi* group and shows a sister-group relationship with *Raorchestes sanjappai* sp. nov. (Fig. 2). For the mitochondrial 16S rRNA, *Raorchestes vellikkannan* sp. nov. is divergent from other members of the group as: 4.6–6.2% from *R. chromasynchysi*; 5.7–6.8% from *R. ravii*; 3.1–3.3% from *R. sanjappai* sp. nov.; and 4.7–5.3% from *R. silentvalley*.

**Morphological diagnosis and comparison.** *Raorchestes vellikkannan* sp. nov. can be distinguished from other known congeners, except members of the *Raorchestes chromasynchysi* group, by the combination of the following morphological characters: small-sized species (male SVL 22.2–22.9 mm); dorsum yellowish-brown; dorsal skin having prominent granular projections with sharply pointed spinules; dorsum with discontinuous or weakly-developed longitudinal mid-dorsal horny ridge; tongue with papillae; foot webbing small, below the second subarticular tubercle on either side of toe IV, I2–2^+^II2–3III2–3^1^/_3_IV3^1^/_2_–2^+^V (Fig. 8).

Within the *Raorchestes chromasynchysi* group, *Raorchestes vellikkannan* sp. nov. differs from *R. chromasynchysi*, *R. ravii*, *R. sanjappai* sp. nov., and *R. silentvalley* by relatively reduced webbing on foot, below the second subarticular tubercle on either side of toe IV, I2–2^+^II2–3III2–3^1^/_3_IV3^1^/_2_–2^+^V (vs. above the second subarticular tubercle on either side of toe IV, I2–2II2–3III2^–^–3^–^IV3^–^–2^–^V in *R. chromasynchysi*; above the second subarticular tubercle on the outer side of toe IV, I2–2II2–3III2^–^–3^1^/_2_IV3^–^–2^–^V in *R. ravii*; up to the third subarticular tubercle on the outer side of toe IV, I2–2II2–3III2^–^–3IV2–2^–^V in *R. sanjappai* sp. nov.; and above the third subarticular tubercle on either side of toe IV, I1^+^–2^+^II2– 3III1^+^–2^–^IV2^–^–1^+^V in *R. silentvalley*). Specifically, it also differs from *R. chromasynchysi* by the outline of its snout rounded to sub-ovoid in dorsal and ventral views (vs. nearly pointed); groin and anterior part of thighs light brown (vs. dark brown with yellow blotches); thigh nearly equal to shank, TL/SHL ratio 0.99–1.0 (vs. thigh relatively shorter than shank TL/SHL ratio 0.89–0.98). Further, it differs from *R. silentvalley* by dorsum predominantly greyish-brown (vs. light or dark green); lateral surfaces of abdomen and groin brown (vs. lateral surfaces of abdomen bright yellow and groin dark blue); ventral surfaces off-white or light grey (vs. yellow); and ventral surfaces of palm and feet light grey (vs. purplish-black). It differs from *R. ravii* by snout rounded to sub-ovoid in ventral view (vs. pointed); snout length nearly equal to the eye diameter, SL/EL ratio 1.0–1.03 (vs. snout longer than eye diameter, SL/EL ratio 1.20–1.42); and thigh nearly equal to shank, TL/SHL ratio 0.99–1.0 (vs. thigh shorter than shank, TL/SHL ratio 0.93–0.95).

For more differences with *R. sanjappai* sp. nov., see the comparison section of that species.

**Description of holotype (*measurements in mm*).** Small-sized adult male (SVL 22.9) with a slender body; head width equal to its length (HW 8.6; HL 8.6; MN 7.7; MFE 5.5; MBE 3.2); outline of the snout rounded to sub-ovoid in dorsal and ventral view, acute in lateral view; snout length (SL 3.2) nearly equal to the horizontal diameter of eye (EL 3.1); loreal region acute with rounded canthus rostralis; tympanum rather indistinct; supratympanic fold rather distinct; tongue emarginate with a lingual papilla. Forearm (FAL 5.2) shorter than hand (HAL 6.6); fingers without lateral dermal fringe; webbing absent; subarticular tubercles rather prominent, rounded, III1 and IV1 weakly-developed; prepollex rather distinct and oval; supernumerary tubercles absent. Hindlimbs moderately long, thigh (TL 12.1) nearly equal to shank (SHL 12.2) and longer than foot (FOL 8.4); distance from heel to tip of toe IV (TFOL 15.1); foot webbing small: I2–2^+^II2–3III2–3^1^/_3_IV3^1^/_2_–2^+^V; dermal fringe absent; subarticular tubercles rather prominent, rounded, simple, IV1 and V1 weakly-developed; supernumerary tubercles absent (Fig. 8).

Dorsal skin shagreened to prominently granular with sharply pointed horny spinules; a weakly-developed and discontinuous mid-dorsal horny ridge; lateral surfaces of the head relatively more granular in comparison to the dorsum; lateral abdominal surfaces shagreened with scattered granular projections; limbs shagreened to sparsely granular. Ventral skin on throat shagreened; chest, belly, and posterior surface of thighs granular; limbs shagreened (Fig. 8).

**Colour of holotype.** *In life*. Dorsum light brown coloured with a yellowish tinge; limbs slightly lighter than dorsal colouration; dorsum with a dark brown ‘X’ shaped marking and scattered brown spots; loreal and tympanic regions darker than dorsum with more brownish spots; lateral abdomen surfaces light yellowish-brown; groin yellowish-brown without prominent markings, with a few irregular dark spots; posterior part of thighs light to dark chocolate brown; limbs light yellowish-brown with dark brown cross-bands; iris silver grey with minute brown speckling. Ventral surface of throat greyish-white with minute brown spots; chest and belly off-white; limbs light greyish-brown; hand and foot dark grey (Fig. 8). *In preservation*. Dorsum light brown with a faint dark ‘X’ mark; upper eyelids light greyish-brown; an inverted light brown triangle placed just below the level of eye; lateral abdominal surfaces light grey; groin greyish-brown; posterior part of thighs light brown; limbs light greyish-brown with dark grey cross-bands. Ventral surfaces off-white without prominent markings, other than minute blackish-brown speckles on throat and limbs (Fig. 8).

**Distribution and natural history.** *Raorchestes vellikkannan* sp. nov. is endemic to the Western Ghats and currently known only from its type locality (Singappara, Siruvani) and surrounding regions of the Silent Valley National Park in Palakkad district of Kerala State, north of Palghat gap. This species was observed inside primary forests and individuals were found on vegetation up to 4 m high during the breeding season. The vocalisations of this species have not been recorded and analysed.

### Taxonomic remarks on three known taxa

***Raorchestes ravii* Zachariah, Dinesh, Kunhikrishnan, Das, Raju, Radhakrishnan, Palot, and Kalesh, 2011**

This taxon was described based on two specimens from “Naduvattam (11° 23′ N 76°34′E; 1,890 m.a.s.l), Nilgiri district, Tamil Nadu, India” (Zachariah et al., 2011). However, subsequent studies could not unambiguously report new collections of this species from its type locality. Vijayakumar et al. (2014) assigned some of their collections from an unknown locality in the Western Ghats (CESF 1154 and CESF 1672) as “*Raorchestes* aff. *ravii*”, which were shown to have a sister-group relationship with *R. chromasynchysi*. However, Zachariah et al. (2016) indicated another population from Vijayakumar et al. (2014)’s study, CESF 469 from an unknown locality in the Western Ghats originally identified as “*R*. aff. *coonoorensis*”, to be “*R. ravii*” in their phylogenetic analyses without any discussion. Here we report new collections (SDBDU 2013.2410–2411) from the type locality Naduvattam, which match with the description and type of *R. ravii*. These are found to be genetically close to “*Raorchestes* aff. *ravii*” (Vijayakumar et al., 2014) (1–1.5% divergence for 16S) and *R. chromasynchysi* (1–2.4% divergence for 16S).

Our morphological examination of the type specimens of *R. ravii* and comparison with *R. chromasynchysi*, as also indicated by Vijayakumar et al. (2014), suggests that *R. ravii* is a member of the *Raorchestes chromasynchysi* group (see ‘Grouping of species using integrative approaches’) due to the combination of characters such as small adult size (male SVL 20–23 mm); pointed snout; dorsal skin with horny spinules; presence of horny ridge between the eyes and another extending from the snout tip to the vent (mentioned as absent by Zachariah et al., 2011; but observed to be present on the holotype); tongue with papillae (mentioned as absent by Zachariah et al., 2011; but observed to be present on the holotype); iris golden brown, horizontally not divided into lighter upper and darker lower halves; and foot webbing extending up to or above the second subarticular tubercle on either side of toe IV. Furthermore, *R. chromasynchysi* that has been reported from Naduvattam is a confusing frog with highly variable colouration and markings, including brown morphs that show the presence of dark X or H-shaped marks on the dorsum, brown band connecting the eyes, lateral surfaces of head and supratympanic region darker brown, flanks and groin without contrasting blotches, and limbs irregularly barred. The original description of *R. ravii* also mentions that its advertisement call is “a distinct bell like musical note similar to water drops falling into water”, which can be acoustically interpreted as a pulsatile call delivered with widely spaced pulses, a characteristic feature of members of the *R. chromasynchysi* group. Hence, based on phylogenetic, morphological, and acoustic evidence, we consider *R. ravii* to be a member of the *R. chromasynchysi* group.

***Raorchestes sanctisilvaticus*** (Das & Chanda, 1997)

Recently three taxa, *Philautus sanctisilvaticus* Das & Chanda, 1997 (=*Raorchestes sanctisilvaticus*), *Philautus similipalensis* Dutta, 2003 (=*Raorchestes similipalensis*) and *Philautus terebrans* Das & Chanda, 1998 (=*Raorchestes terebrans*), were shown to be conspecific due to overlapping morphological characters and shallow genetic divergence (Mirza et al., 2019). Consequently, the first available name, *Philautus sanctisilvaticus* Das & Chanda, 1997 (=*Raorchestes sanctisilvaticus*) was recognised as the valid name, with which the latter two taxa (*Philautus similipalensis* and *P. terebrans*) were synonymised.

A further comparative study of *Raorchestes sanctisilvaticus* (from its type locality, along with typical and additional populations referring to its two synonyms) and *R. bombayensis* was herein carried out. Based on available and additional new morphological, phylogenetic, and acoustic evidence, we find that *R. sanctisilvaticus* is closely related to, and could possibly be considered conspecific with, *R. bombayensis*. Morphologically, the foot webbing of *R. similipalensis* and *R. terebrans* differs from that in *R. sanctisilvaticus*, whereas some populations of *R. sanctisilvaticus* have a relatively less mottled ventral skin (vs. prominently mottled in some populations of *R. bombayensis*). Both these morphological characters differ feebly and are also observed to be variable among the various different populations referring to these four species.

Acoustically, the non-pulsatile call of *R. sanctisilvaticus* is very similar to that of *R. bombayensis* and *R. similipalensis*, while the call of *R. terebrans* is slightly longer in duration, 12.2 ms (vs. 11.6 ms, 12.1 ms and 16.1 ms, respectively). The dominant frequency of calls of all the four species is similar (approximately 3 kHz).

Furthermore, the interspecific divergence for the mitochondrial 16S between *R. bombayensis* (from northern Karnataka, Goa, and Maharashtra) and *R. sanctisilvaticus* (from Madhya Pradesh, Odisha, Chhattisgarh, and Andhra Pradesh) is 0.8–1.5%, which overlaps with the intraspecific divergence among different populations of *R. bombayensis* (up to 0.8%) and *R. sanctisilvaticus* (up to 0.8%).

Hence, since all these populations (for which various different names are currently available) neither have considerable genetic divergences nor can they be reliably distinguished from each other based on morphology or calls, and further in the light of recent taxonomic actions by Mirza et al. (2019), the most parsimonious taxonomic resolution would be to consider them as a single species, for which the earliest available name *Ixalus bombayensis* Annandale, 1919 (=*Raorchestes bombayensis*) should be applied, with *Philautus sanctisilvaticus* Das & Chanda, 1997, *Philautus similipalensis* Dutta, 2003 (=*Raorchestes sanctisilvaticus*) and *Philautus terebrans* Das & Chanda, 1998 (=*Raorchestes sanctisilvaticus*) as its junior subjective synonyms. However, we currently refrain from any taxonomic action in this regard, pending further population level studies with comprehensive sampling across the entire known range of *R. bombayensis* and this taxon.

***Raorchestes thodai* Zachariah, Dinesh, Kunhikrishnan, Das, Raju, Radhakrishnan, Palot, and Kalesh, 2011**

This species was described based on two specimens from “Ooty (Udhagamandalam) town (11° 24′ N; 76° 40′ E; 1980 m.a.s.l), Nilgiris district, Tamil Nadu, India” primarily based on iris, flanks, and groin colouration. However, subsequent studies could not gather new collections conspecific to this species from its type locality (Vijayakumar et al., 2014). During our herpetological surveys in the Nilgiris over the past decade, we have not encountered any new samples referable to this species. Furthermore, the level of morphological differentiation between *R. thodai* and the closely allied *R. signatus* remains unclear due to several overlapping characters, such as dorsal colouration and marking as well as presence or absence of radiating lines on iris, which are observed to be highly variable even among individuals of the same population of *R. signatus*, thereby not permitting a distinction between the two taxa. Hence, *R. thodai* Zachariah, Dinesh, Kunhikrishnan, Das, Raju, Radhakrishnan, Palot, and Kalesh, 2011 is likely to be a junior subjective synonym of *R. signatus* (Boulenger, 1882).

### Grouping of species using integrative approaches

**Genus:** *Raorchestes* Biju, Shouche, Dubois, Dutta, and Bossuyt, 2010

**Proposed common name:** Rao’s Oriental Shrub Frogs

**Figure 9:**
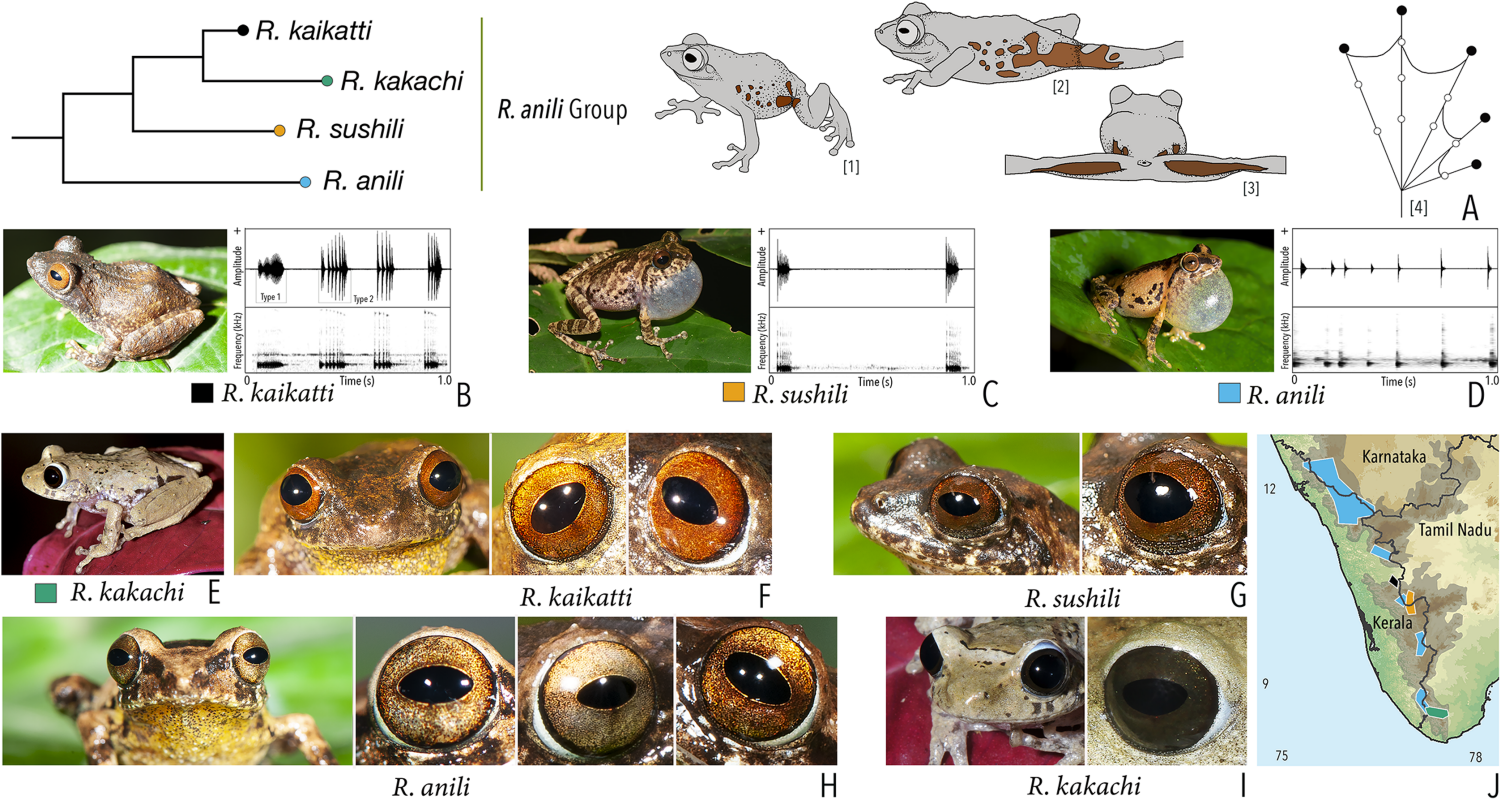
*Raorchestes anili* species group. (A) Phylogenetic relationships and major morphological characters for members of the group: (1) adult size range (male SVL 22–27 mm, female SVL 25–36 mm); (2) flank and groin with dark brown blotches; (3) anterior and posterior surfaces of thigh and inner side of shank dark brown; (4) foot webbing moderate, up to the third subarticular tubercle on either side of toe IV. (B–D) Male calls in three studied species of the group: (B) Adult male, followed by a call oscillogram (above) and spectrogram (below) for *R. kaikatti* (1 s section showing a single Type 1 and three Type 2 calls, delivered in groups). (C and D) Calling individuals, followed by call oscillograms (above) and spectrograms (below) for *R. sushili* (1 s section showing two calls, delivered in groups) and *R. anili* (1 s section showing a single call). (E) Adult male of *R. kakachi* (call not studied). (F–I) Eye colour and pattern in four members of the group. (J) Geographical distribution of members of the group; species range colours on the map correspond to the square colours indicated alongside each species label in (B)–(E). Photo of calling male in (9D) by Karthik A. K.

***Raorchestes anili*** group

(Figs. 2 and 9; Tables 1–3)

**Members included.** *Raorchestes anili, R. kaikatti*, *R. kakachi*, and *R. sushili*.

**Group definition. *Phylogenetic*.** The *Raorchestes anili* group can be characterised as the most inclusive clade and a Western Ghats radiation that contains *R. anili, R. kaikatti*, *R. kakachi*, and *R. sushili*, but none of the other currently recognised *Raorchestes* species (Fig. 2). This group is analogous to the Anili clade of Vijayakumar et al. (2014). ***Morphological*.** Members of this group can be diagnosed based on the combination of the following characters: small to medium-sized adults (male SVL 22–27 mm, female SVL 25–36 mm); dorsum with shades of grey to brown, predominantly with dark irregular markings; canthus rostralis sharp or rounded; flank and groin light to dark brown, with white blotches; anterior and posterior surfaces of thigh and inner side of shank dark brown, with dark contrasting blotches or bands; lateral surfaces granular with white or light brown spots; foot webbing moderate, not extending beyond the third subarticular tubercle on either side of toe IV (Fig. 9A). ***Eye colouration and pattern***. Iris colour varies from light to dark brown or reddish-brown, occasionally with golden tinge (as in *R. anili*); iris periphery dark brown; sclera light silvery-blue (Figs. 9F–9I; Table 1). ***Acoustic*.** Three members of the group for which calls are studied (*R. anili, R. kaikatti*, and *R. sushili*) produce one (*R. anili* and *R. sushili*) or two (*R. kaikatti*) types of calls. Calls of all the three species have a pulsatile temporal structure. The overall dominant frequency of the calls ranges from 2.4 to 2.9 kHz (Figs. 9B–9D). ***Calling height*.** Members of this group usually call from heights of 1.5–5 m on higher shrubs or in the lower tree canopy (Table 2). ***Geographical*.** This group is currently restricted to Western Ghats regions in Karnataka, Kerala, and Tamil Nadu States (Fig. 9J; Table 3).

**Species level acoustic comparison.** Within the *R. anili* group, the calls of *R. anili*, *R. sushili*, and the Type 1 call of *R. kaikatti* have distinct call structures and can be differentiated by their call duration and pulse rate. Calls of *R. anili* differ from those of *R. sushili* and *R. kaikatti* due to their longer duration, 980.4 ms (vs. 51.2 ms and 102.7 ms, respectively), and a relatively slow pulse rate with delivery at 6.3 pulses/s (vs. relatively similar in the other two species, 242.8 pulses/s and 280.8 pulses/s, respectively). The dominant frequencies of the calls of *R. sushili* and *R. kaikatti* are relatively similar (2.6 kHz and 2.4 kHz, respectively) and somewhat lower compared to *R. anili* (2.9 kHz) (Figs. 9B–9D; Table 2).

**Figure 10:**
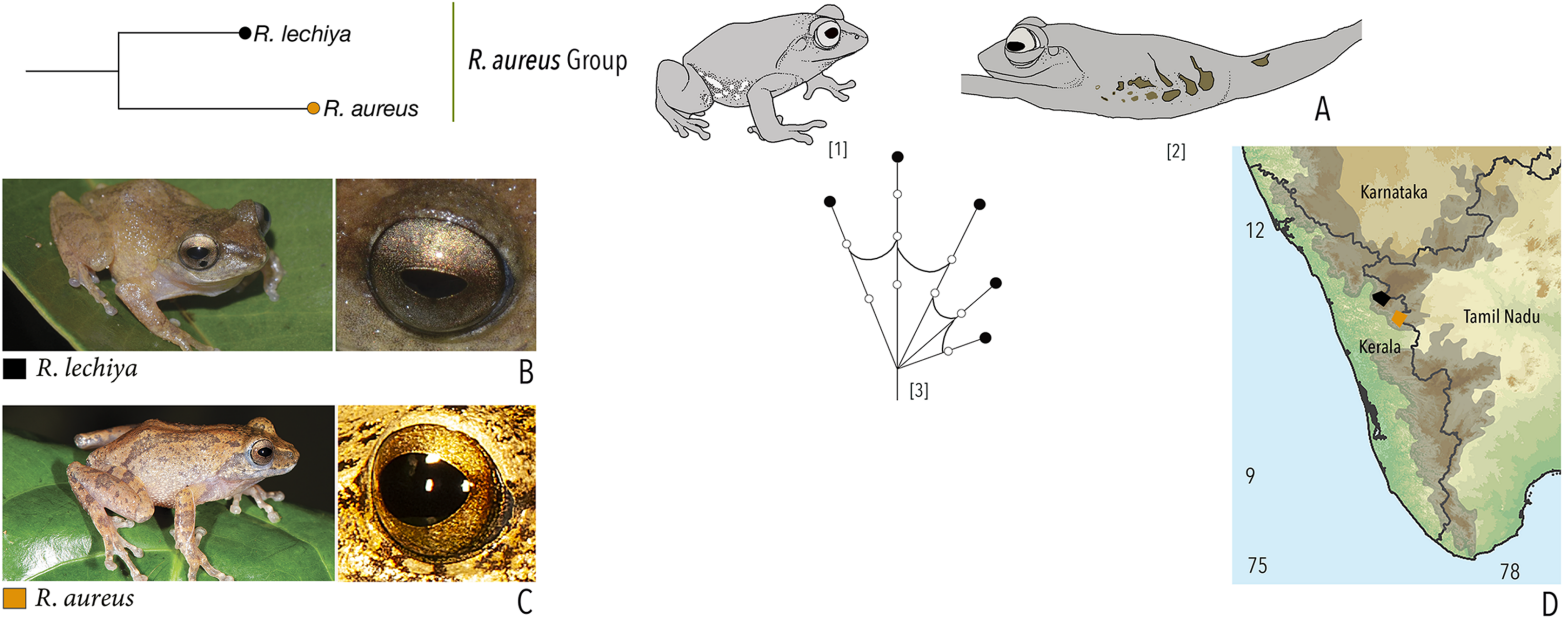
*Raorchestes aureus* species group. (A) Phylogenetic relationships and major morphological characters for members of the group: (1) adult size range (male SVL 24–25 mm, female SVL 29–30 mm); (2) lateral abdominal surfaces and groin with irregular mottling, spots, or patches; (3) foot webbing small, not extending beyond the second subarticular tubercle on either side of toe IV. (B and C) Adult individuals and eye colour and pattern in two members of the group. (D) Geographical distribution of members of the group; species range colours on the map correspond to the square colours indicated alongside each species label in (B) and (C).

***Raorchestes aureus*** group

(Figs. 2 and 10; Tables 1–3)

**Members included.** *Raorchestes aureus* and *R. lechiya*.

**Group definition. *Phylogenetic*.** The *Raorchestes aureus* group can be characterised as the most inclusive clade and a Western Ghats radiation that contains *R. aureus and R. lechiya*, but none of the other currently recognised *Raorchestes* species (Fig. 2). ***Morphological*.** Members of this group can be diagnosed based on the combination of the following characters: small to medium-sized adults (male SVL 24–25 mm, female SVL 29 mm); dorsum with shades of grey or brown to light yellow, with or without dark irregular markings; dorsal skin shagreened to granular bearing spinular projections; snout sub-elliptical in dorsal and ventral view, rounded in lateral view; lateral abdominal surfaces and groin with irregular mottling, spots, or patches; ventral surfaces with faint to prominent mottling or vermiculation; foot webbing small, not extending beyond the second subarticular tubercle on either side of toe IV (Fig. 10A). ***Eye colouration and pattern*.** Iris colour golden brown; iris periphery black; sclera light silvery-blue (Figs. 10B and 10C; Table 1). ***Acoustic*.** Members of this group (*R. aureus* and *R. lechiya*) produce a single type of call (Zachariah et al., 2016). The calls are non-pulsatile in *R. aureus* while they have a pulsatile temporal structure in *R. lechiya*. The overall dominant frequency of the calls in these species range from 2.6–3.0 kHz (Zachariah et al., 2016) (Table 2). ***Calling height*.** Members of this group usually call from heights of 0.5–2 m on low bushes or higher shrubs (Table 2). ***Geographical*.** This group is currently restricted to Western Ghats regions north of Palghat gap in Kerala and Tamil Nadu States (Fig. 10D; Table 3).

**Figure 11:**
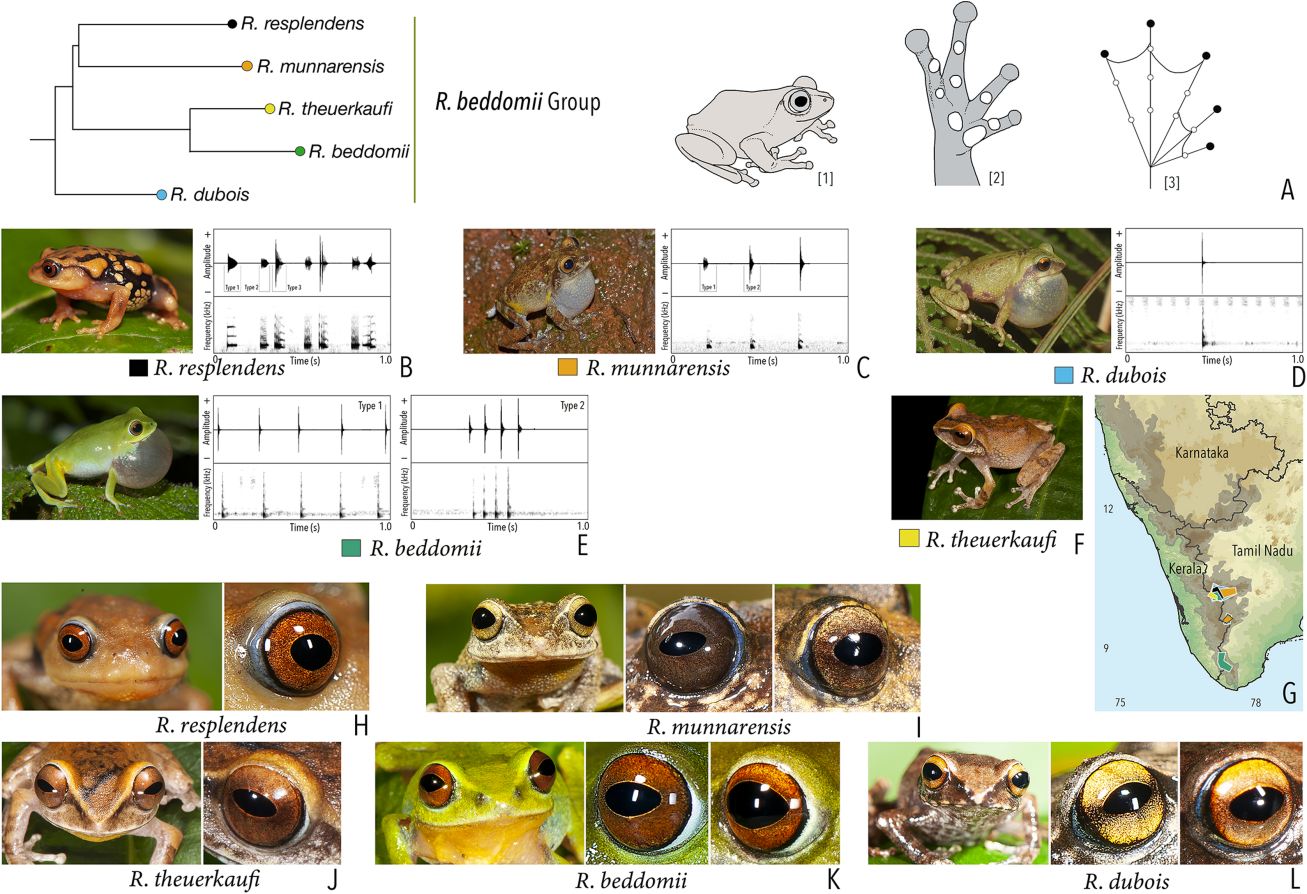
*Raorchestes beddomii* species group. (A) Phylogenetic relationships and major morphological characters for members of the group: (1) adult size range (male SVL 17–35 mm, female SVL 21–39 mm); (2) prominent subarticular tubercles on hand and foot; (3) foot webbing moderate, up to the third subarticular tubercle on either side of toe IV. (B–E) Male calls in members of the group: (B) Adult male, followed by a call oscillogram (above) and spectrogram (below) for *R. resplendens* (1 s section showing a single Type 1 call, four Type 2 calls, and two Type 3 calls, delivered in groups). (C) Calling individual, followed by a call oscillogram (above) and spectrogram (below) for *R. munnarensis* (1 s section showing a single Type 1 call and two Type 2 calls, delivered in groups). (D) Calling individual, followed by a call oscillogram (above) and spectrogram (below) for *R. dubois* (1 s section showing a single call). (E) Calling individual, followed by call oscillograms (above) and spectrograms (below) for the two call types in *R. beddomii*, Type 1 (1 s section showing a single call) and Type 2 (1 s section of a call group showing five calls). (F) Adult male of *R. theuerkaufi* (call not studied). (G) Geographical distribution of members of the group; species range colours on the map correspond to the square colours indicated alongside each species label in (B)–(F). (H–L) Eye colour and pattern in five members of the group.

***Raorchestes beddomii*** group

(Figs. 2 and 11; Tables 1–3)

**Members included.** *Raorchestes beddomii, R. dubois*, *R. resplendens, R. munnarensis*, and *R. theuerkaufi*.

**Group definition. *Phylogenetic*.** The *Raorchestes beddomii* group can be characterised as the most inclusive clade and a Western Ghats radiation that contains *R. beddomii, R. dubois*, *R. resplendens, R. munnarensis*, and *R. theuerkaufi*, but none of the other currently recognised *Raorchestes* species (Fig. 2). This group is analogous to the Beddomii clade of Vijayakumar et al. (2014). ***Morphological*.** Members of this group can be diagnosed based on the combination of the following characters: small to large-sized adults (male SVL 17–35 mm, female SVL 21–39 mm); subarticular tubercles rather prominent both on hands and feet; foot webbing small to moderate, not extending beyond the third subarticular tubercle on either side of toe IV (Fig. 11A). ***Eye colouration and pattern***. Iris light to dark red, except light golden brown to dark brown with or without reddish tinge in *R. dubois* and *R. munnarensis*; iris periphery black, sclera light blue to scarlet blue (Figs. 11H–11L; Table 1). ***Acoustic*.** Four members of the group for which calls are studied (*R. beddomii, R. dubois*, *R. resplendens*, and *R. munnarensis*) produce one (*R. dubois*), two (*R. beddomii* and *R. munnarensis*), or three (*R. resplendens*) types of call. The most common call type (Type 1) are non-pulsatile in *R. resplendens* and *R. dubois*, while they have a pulsatile temporal structure in *R. beddomii* and *R. munnarensis*. The overall dominant frequency of calls in these species range from 2.2 to 2.7 kHz (Figs. 11B–11E; Table 2). ***Calling height*.** Members of this group usually call from ground level, low bushes and shrubs, to tree canopy layers of up to 20 m high (Table 2). ***Geographical*.** This group is currently restricted to the Western Ghats regions south of Palghat gap in the States of Kerala and Tamil Nadu (Fig. 11G; Table 3).

**Species level acoustic comparison.** Within the *R. beddomii* group, the four species have considerably different call structures. The Type 1 non-pulsatile call in *R. resplendens* (51.3 ms) is relatively longer than that in *R. dubois* (10.6 ms). Whereas the Type 1 pulsatile in *R. munnarensis* is longer (498.2 ms) and delivered at a slower pulse rate (6.4 pulses/s) compared to *R. beddomii*, which produces a call that is 150 ms in duration and delivered at 29.2 pulses/s. The dominant frequency of Type 1 calls of three species, *R. beddomii, R. resplendens* and *R. dubois*, are similar (2.5–2.7 kHz), while that of *R. munnarensis* is lower (2.2 kHz) (Figs. 11B–11E; Table 2).

**Figure 12:**
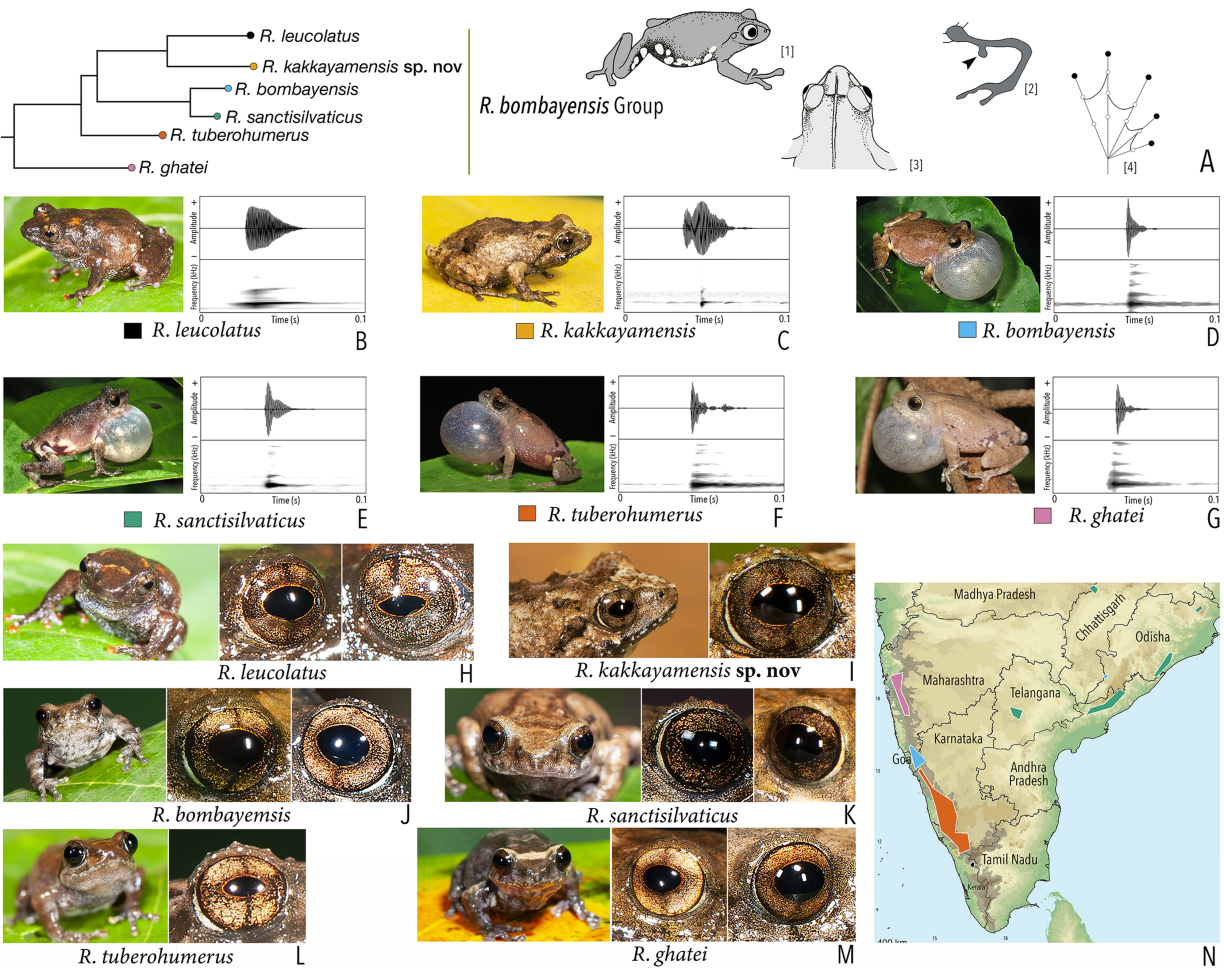
*Raorchestes bombayensis* species group. (A) Phylogenetic relationships and major morphological characters for members of the group: (1) adult size range (male SVL 17–28 mm, female SVL 18–32 mm); lateral surfaces of abdomen, groin, and posterior part of thighs marbled with contrasting white or yellow blotches on dark brown or black background; (2) a knobbed bony projection on humerus in males; (3) presence of horny spinules and/or horny ridges on dorsal skin; (4) foot webbing small to moderate, not extending beyond the third subarticular tubercle on either side of toe IV. (B–G) Male calls in members of the group: (B and C) Adult males, followed by call oscillograms (above) and spectrograms (below) for *R. leucolatus* and *R. kakkayamensis* sp. nov. (0.1 s sections showing a single call for each species). (D, E, F and G) Calling individuals, followed by call oscillograms (above) and spectrograms (below) for *R. bombayensis*, *R. tuberohumerus*, *R. ghatei*, and *R. sanctisilvaticus* (0.1 s sections showing a single call for each species). (H–M) Eye colour and pattern in six members of the group. (N) Geographical distribution of members of the group; species range colours on the map correspond to the square colours indicated alongside each species label (B)–(G). Photo of calling male in (G) by Amit Sayyed.

***Raorchestes bombayensis*** group

(Figs. 2 and 12; Tables 1–3)

**Members included.** *Raorchestes bombayensis, R. ghatei, R. kakkayamensis* sp. nov., *R. leucolatus, R. sanctisilvaticus*, and *R. tuberohumerus*.

**Group definition. *Phylogenetic*.** The *Raorchestes bombayensis* group can be characterised as the most inclusive clade and a Western Ghats radiation that contains *R. bombayensis, R. ghatei, R. kakkayamensis* sp. nov., *R. leucolatus*, *R. sanctisilvaticus*, and *R. tuberohumerus*, but none of the other currently recognised *Raorchestes* species (Fig. 2). This group is analogous to the Bombayensis clade of Vijayakumar et al. (2014). ***Morphological*.** Members of this group can be diagnosed based on the combination of the following characters: small to medium-sized adults (male SVL 17–28 mm, female SVL 18–32 mm); dorsum predominantly brown; a knobbed bony projection on humerus in males (visible externally in preserved specimens); lateral surfaces of abdomen marbled with contrasting white or yellow blotches on brown or black background; presence of horny spinules and/or horny ridges on dorsal skin; foot webbing small to moderate, not extending beyond the first subarticular tubercle on either side of toe IV (Fig. 12A). ***Eye colouration and pattern***. All members of the group have a brown iris with dense golden speckling and dark brown horizontal and vertical bands; iris periphery dark brown; sclera light silvery blue (Figs. 12H–12M; Table 1). ***Acoustic*.** Six members of the group for which calls are studied (*R. bombayensis, R. ghatei, R. kakkayamensis* sp. nov., *R. leucolatus*, *R. sanctisilvaticus*, and *R. tuberohumerus*) produce a single type of call with non-pulsatile temporal structure. The overall dominant frequency of the calls for these species ranges from 2.9 to 4.1 kHz (Figs. 12B–12F; Table 2). ***Calling height*.** Members of this group usually call from heights of 0.5–5 m on low bushes or higher shrubs (Table 2). ***Geographical*.** Members of this group are widely distributed right from the Western Ghats regions north of Palghat gap in the States of Kerala, Karnataka, Maharashtra, and Goa, up to the Eastern Ghats (Andhra Pradesh, Telangana, and Odisha) and Central India (Madhya Pradesh and Chhattisgarh) (Fig. 12N; Table 3).

**Species level acoustic comparison.** All members of the *R. bombayensis* group have similar call structure but *R. kakkayamensis* sp. nov. and *R. leucolatus* produce relatively longer calls, 25.3 ms and 29.4 ms, respectively (vs. 11.6 ms in *R. bombayensis*, 12.2 ms in *R. sanctisilvaticus*, 17.7 ms in *R. tuberohumerus*, and *R. ghatei* in 14.6 ms). The call of *R. kakkayamensis* sp. nov. has a longer rise time (9.2 ms) compared to *R. leucolatus* (2.7 ms), whereas the other members show very short rise time ≤1.5 ms. The call of *R. leucolatus* also has a relatively higher dominant frequency (4.1 kHz) compared to *R. kakkayamensis* sp. nov. (3.8 kHz), while other members, *R. bombayensis* (3.1 kHz), *R. sanctisilvaticus* (3.1 kHz), *R. tuberohumerus* (3.3 kHz), and *R. ghatei* (2.9 kHz) have a lower dominant frequency in comparison to *R. kakkayamensis* sp. nov. (Figs. 12B–12G; Table 2).

**Figure 13:**
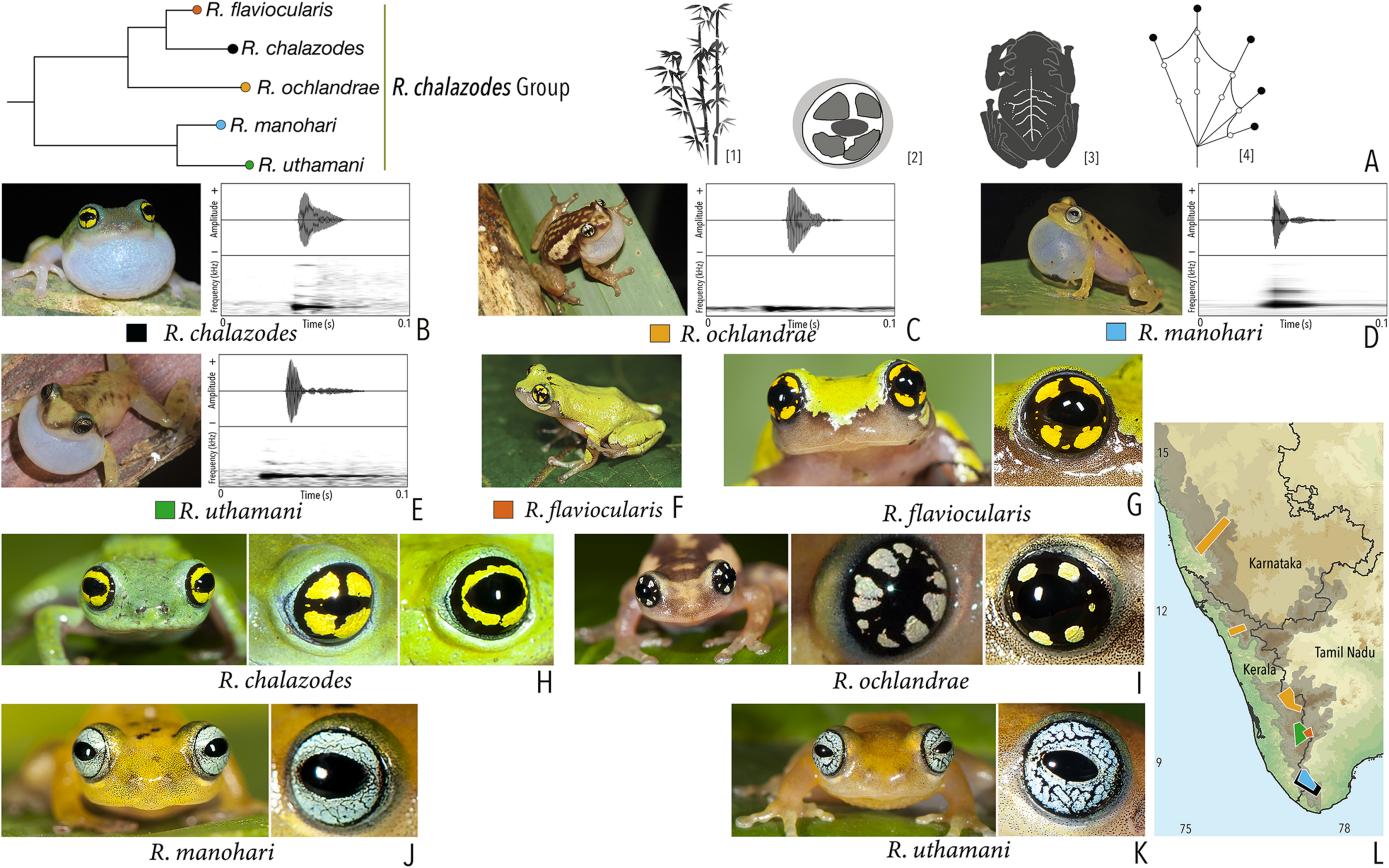
*Raorchestes chalazodes* species group. (A) Phylogenetic relationships and major diagnostic characters for members of the group: (1) largely associated with bamboo reeds in forested areas, especially for breeding; (2) iris black with a golden yellow ring that may or may not be divided into patches by a black cross mark or radiating pattern, or dense metallic silver with fine black reticulations forming a mosaic pattern; (3) central part of belly translucent with venations; (4) foot webbing moderate, extending up to the third subarticular tubercle on either side of toe IV. (B–E) Male calls in members of the group: (B, C, D and E) Calling individuals, followed by call oscillograms (above) and spectrograms (below) for *R*. *chalazodes*, *R. ochlandrae*, *R. manohari*, and *R. uthamani* (0.1 s sections showing a single call for each species). (F) Adult male of *R. flaviocularis* (call not studied). (G–K) Eye colour and pattern in five members of the group. (L) Geographical distribution of members of the group; species range colours on the map correspond to the square colours indicated alongside each species label in (B)–(F). Photo of calling male in (C) by Gururaja K. V.

***Raorchestes chalazodes*** group

(Figs. 2 and 13; Tables 1–3)

**Members included.** *Raorchestes chalazodes, R. flaviocularis*, *R. ochlandrae, R. manohari*, and *R. uthamani*.

**Group definition. *Phylogenetic*.** The *Raorchestes chalazodes* group can be characterised as the most inclusive clade and a Western Ghats radiation that contains *R. chalazodes, R. flaviocularis*, *R. ochlandrae, R. manohari*, and *R. uthamani*, but none of the other currently recognised *Raorchestes* species (Fig. 2). This group is analogous to the Chalazodes clade of Vijayakumar et al. (2014). ***Morphological*.** Members of this group can be diagnosed based on the combination of the following characters: small to medium-sized adults (male SVL 17–29 mm, female SVL 28–37 mm); snout rounded to semi-circular in ventral view; dorsum uniformly green, yellow, or brown with scattered spots, longitudinal markings or dorsolateral bands; distinctly patterned iris, black or dark brown with golden yellow patches, or silvery white with thin black reticulations; presence of lingual papillae; subarticular tubercles rather prominent on both hands and feet; moderate webbing on feet; and central part of belly translucent with venations (Fig. 13A). ***Eye colouration and pattern***. Members of this group have a distinct eye colour and pattern, with blackish-brown iris having a golden yellow ring that may or may not be divided by a black cross mark or a radial pattern (*R. chalazodes, R. flaviocularis*, and *R. ochlandrae*) or iris brown with dense metallic silver mosaic pattern (*R. manohari* and *R. uthamani*); iris periphery brown; sclera scarlet blue or indistinct (Figs. 13H–13K; Table 1). ***Acoustic*.** Four members of the group for which calls are studied (*R. chalazodes, R. ochlandrae, R. manohari*, and *R. uthamani*) produce a single type of call with very similar call structure. The calls have a non-pulsatile temporal structure with duration typically ranging between 12.3 and 36 ms. The calls are generally delivered rapidly in long call groups. The overall dominant frequency of the calls ranges from 2.7 to 3.6 kHz (Figs. 13B–13H; Table 2). ***Calling height*.** Members of this group usually call from heights of 1–7 m on low to medium-sized reeds and high bamboo tree canopy (Table 2). ***Geographical*.** This group is currently restricted to the Western Ghats regions in southern Karnataka, Kerala, and Tamil Nadu States (Fig. 13L; Table 3). ***Habitat preference*.** Another unique characteristic of this group is the habitat association of its members largely with bamboo reeds in forested areas.

**Species level acoustic comparison.** Within the *R. chalazodes* group, the four species have very similar call structures consisting of non-pulsatile calls, each having a short rise time and relatively longer fall time, that are organised into long call groups of rapidly produced calls. The call duration of these species varies, with *R. uthamani* showing a longer call (36 ms) compared to *R. chalazodes* (18.4 ms), *R. ochlandrae* (25.3 ms), and *R. manohari* (12.3 ms). The dominant frequency of the calls of two species, *R. chalazodes* and *R. ochlandrae*, is similar (2.7 kHz and 2.7 kHz, respectively), while the calls of *R. manohari* and *R. uthamani* are both produced at a higher dominant frequency (3.6 kHz and 3.4 kHz, respectively) (Figs. 13B–13H; Table 2).

**Figure 14:**
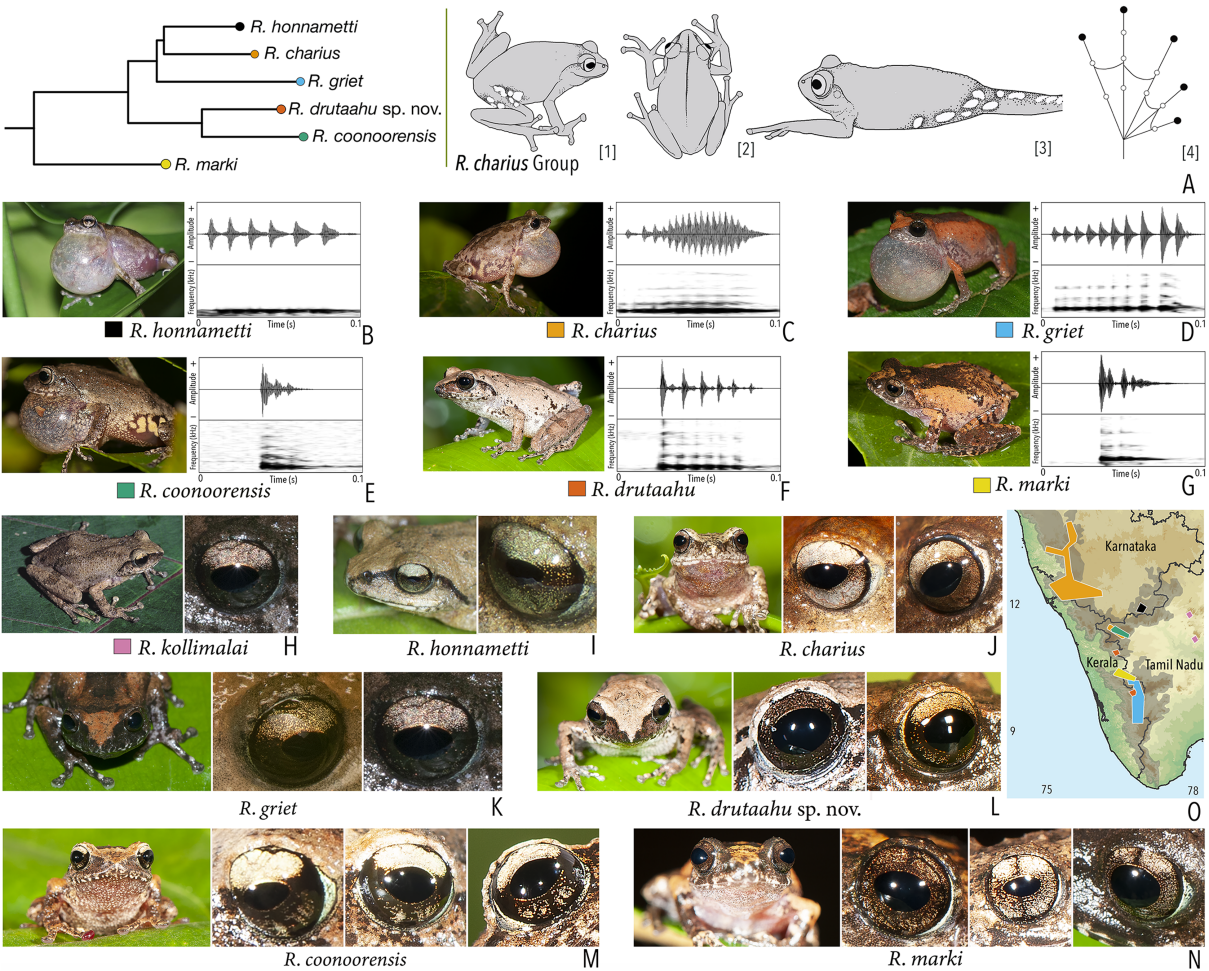
*Raorchestes charius* species group. (A) Phylogenetic relationships and major morphological characters for members of the group: (1) adult size range (male SVL 25–35 mm; female SVL 25–39 mm); (2) horny ridges between the eyes, arranged in a triangle directed posteriorly; (3) groin light or dark brown predominantly with white or yellow blotches; (4) foot webbing small, below the second subarticular tubercle on either side of toe IV. (B–G) Male calls in members of the group: (B, C, D and E) Calling individuals, followed by call oscillograms (above) and spectrograms (below) for *R*. *honnametti*, *R. charius*, *R. griet*, and *R. coonoorensis* (0.1 s sections showing a single call for each species). (F and G) Adult male, followed by a call oscillogram (above) and spectrogram (below) for *R. drutaahu* sp. nov. and *R. marki* (0.1 s section showing a single call for each species). (H) Adult male of *R. kollimalai* (call not studied). (H–N) Eye colour and pattern in seven members of the group. (O) Geographical distribution of members of the group; species range colours on the map correspond to the square colours indicated alongside each species label in (B)–(H). Photo of calling male in (B) by Yatin Kalki.

***Raorchestes charius*** group

(Figs. 2 and 14; Tables 1–3)

**Members included.** *Raorchestes charius, R. coonoorensis, Raorchestes drutaahu* sp. nov., *R. griet, R. honnametti*, *R. kollimalai*, and *R. marki*.

**Group definition. *Phylogenetic*.** The *Raorchestes charius* group can be characterised as the most inclusive clade and a Western Ghats radiation that contains *R. charius, R. coonoorensis, R. drutaahu* sp. nov., *R. griet, R. honnametti*, *R. marki* (Fig. 2), and *R. kollimalai* (Gowande, Ganesh & Mirza, 2020), but none of the other currently recognised *Raorchestes* species. This group is analogous to the Charius and Coonoorensis clades of Vijayakumar et al. (2014). ***Morphological*.** Members of this group can be diagnosed based on the combination of the following characters: small to medium-sized adults (male SVL 25–35 mm, female SVL 25–39 mm); dorsum light to dark brown usually with a prominent thin mid-dorsal line; a prominent horny ridge extending from the tip of snout to the vent, and a horny ridge between the eyes, arranged in a triangle directed posteriorly; dorsal skin with spinules and/or glandular projections; groin light or dark brown with white or yellow blotches, except *R. drutaahu* sp. nov.; foot webbing small, below the second subarticular tubercle on either side of toe IV (Fig. 14A). ***Eye colouration and pattern***. This group has a distinct eye colour and pattern and all the members have a light to dark brown iris with golden tinge or dense golden speckling, and iris horizontally divided into light upper and dark lower halves; iris periphery blackish-brown or black; sclera light blue to light bluish-grey (Figs. 14H–14M; Table 1). ***Acoustic*.** Six known members of the group for which calls are studied (*R. charius, R. coonoorensis, R. drutaahu* sp. nov., *R. griet, R. honnametti*, and *R. marki*) produce a single type of call that is short and has a pulsatile temporal structure. The calls also have closely packed pulses and a relatively fast pulse rate. The overall dominant frequency of the calls ranges from 2.4 to 3.6 kHz (Figs. 14B–14G; Table 2). ***Calling height*.** Members of this group usually call from ground level, low bushes and shrubs, and higher shrubs of up to 4 m high (Table 2). ***Geographical*.** Members of this group are currently restricted to the States of Karnataka, Kerala, and Tamil Nadu, both in the Western Ghats and Eastern Ghats regions (Fig. 14N; Table 3).

**Species level acoustic comparison.** Within the *R. charius* group, all members produce calls with an overall similar structure. *R. charius, R. griet* and *R. honnametti* produce relatively longer calls (92.6 ms, 75.6 ms and 68.6 ms, respectively) compared to *R. coonoorensis, R. marki* and *R. drutaahu* sp. nov. (24.6 ms, 36.7 ms and 50.6 ms, respectively). The calls of former three species also have a longer call rise time (49.2 ms, 59.4 ms and 13.3 ms, respectively) compared to the latter three (1.2 ms, 1.3 ms and 1.2 ms, respectively). The pulse rate of all five species also varies considerably. *Raorchestes drutaahu* sp. nov., *R. honnametti* and *R. griet* produce calls with relatively slower pulse rate (134.5 pulses/s, 89.3 pulses/s and 151.3 pulses/s, respectively) compared to the calls of *R. marki*, *R. charius* and *R. coonorensis* (266.6 pulses/s, 226.6 pulses/s and 200 pulses/s, respectively). *Raorchestes drutaahu* sp. nov., *R. griet* and *R. marki* have a relatively higher dominant frequency (3.6 kHz, 3.5 kHz and 4.1 kHz, respectively) compared to *R. charius, R. honnametti*, and *R. coonoorensis* (2.4 kHz, 2.6 kHz and 2.9 kHz, respectively) (Figs. 14B–14G; Table 2).

**Figure 15:**
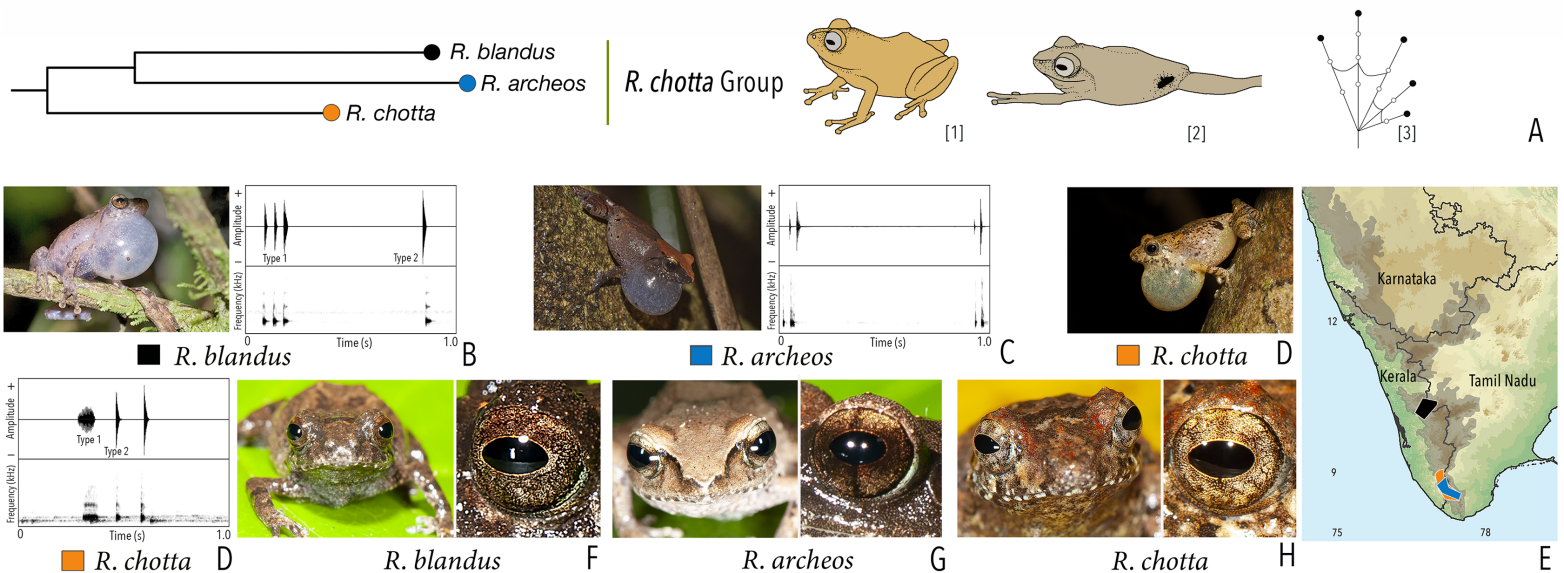
*Raorchestes chotta* species group. (A) Phylogenetic relationships and major morphological characters for members of the group: (1) adult size range (male SVL 16–22 mm, female SVL 19–28 mm); (2) two black or dark brown spots on either side of the lumbar region; (3) foot webbing small, not extending beyond the second subarticular tubercle on either side of toe IV. (B–D) Male calls in members of the group: (B, C and D) Calling individuals, followed by call oscillograms (above) and spectrograms (below) for *R*. *blandus* (1 s section showing three Type 1 calls delivered in group and a single Type 2 call), *R. archeos* (1 s section showing four calls, delivered in groups), and *R. chotta* (1 s section showing a single Type 1 calls and two Type 2 calls, delivered in group). (E) Geographical distribution of members of the group; species range colours on the map correspond to the square colours indicated alongside each species label in (B)–(D). (F–H) Eye colour and pattern in three members of the group.

***Raorchestes chotta*** group

(Figs. 2 and 15; Tables 1–3)

**Members included.** *Raorchestes archeos*, *R. blandus*, and *R. chotta*.

**Group definition. *Phylogenetic*.** The *Raorchestes chotta* group can be characterised as the most inclusive clade and a Western Ghats radiation that contains *R. archeos, R. chotta*, and *R. blandus*, but none of the other currently recognised *Raorchestes* species. (Fig. 2). This group is analogous to the Chotta clade of Vijayakumar et al. (2014). ***Morphological*.** Members of this group can be diagnosed based on the combination of the following characters: small-sized adults (male SVL 16–22 mm, female SVL 19–28 mm); dorsum predominantly light to dark brown; spinular projections on upper eyelids; two black or dark brown spots on either side of the lumbar region; and foot webbing small, not extending beyond the second subarticular tubercle on either side of toe IV (Fig. 15A). ***Eye colouration and pattern***. Iris golden brown in all three members of the group, with vertical band (in *R. archeos*); iris periphery brown, thin and discontinuous; sclera greyish-white (Figs. 15F–15H; Table 1). ***Acoustic*.** Members of this group produce one (*R. archeos*) or two (*R. chotta* and *R. blandus*) types of call. The most common call type (Type 1) is pulsatile for all the three members, whereas the Type 2 call having a single pulse is found only in *R. chotta* and *R. blandus*. The overall dominant frequency of Type 1 calls range from 3.1 to 3.6 kHz (Figs. 15B–15D; Table 2). ***Calling height*.** Members of this group usually call from ground level, low bushes and shrubs, and higher shrubs of up to 4 m high (Table 2). ***Geographical*.** This group is currently restricted to the Western Ghats regions in the States of Karnataka, Kerala, and Tamil Nadu (Fig. 15E; Table 3).

**Species level acoustic comparison.** Within the *R. chotta* group, the Type 1 calls of *R. chotta, R. archeos*, and *R. blandus* have a similar structure but can be differentiated by their duration and pulse rate. The Type 1 calls of *R. chotta* are longer in duration (71.2 ms) and delivered at a rate of 283.5 pulses/s compared to the calls of *R. archeos* and *R. blandus* (19.7 ms and 17.1 ms, respectively), which are delivered at a faster rate (382.1 pulses/s and 370.3 pulses/s, respectively). The dominant frequencies of the calls of *R. chotta* and *R. blandus* are similar and of higher frequency (3.6 kHz and 3.5 kHz, respectively) compared to *R. archeos* (3.1 kHz) (Figs. 15B–15D; Table 2).

**Figure 16:**
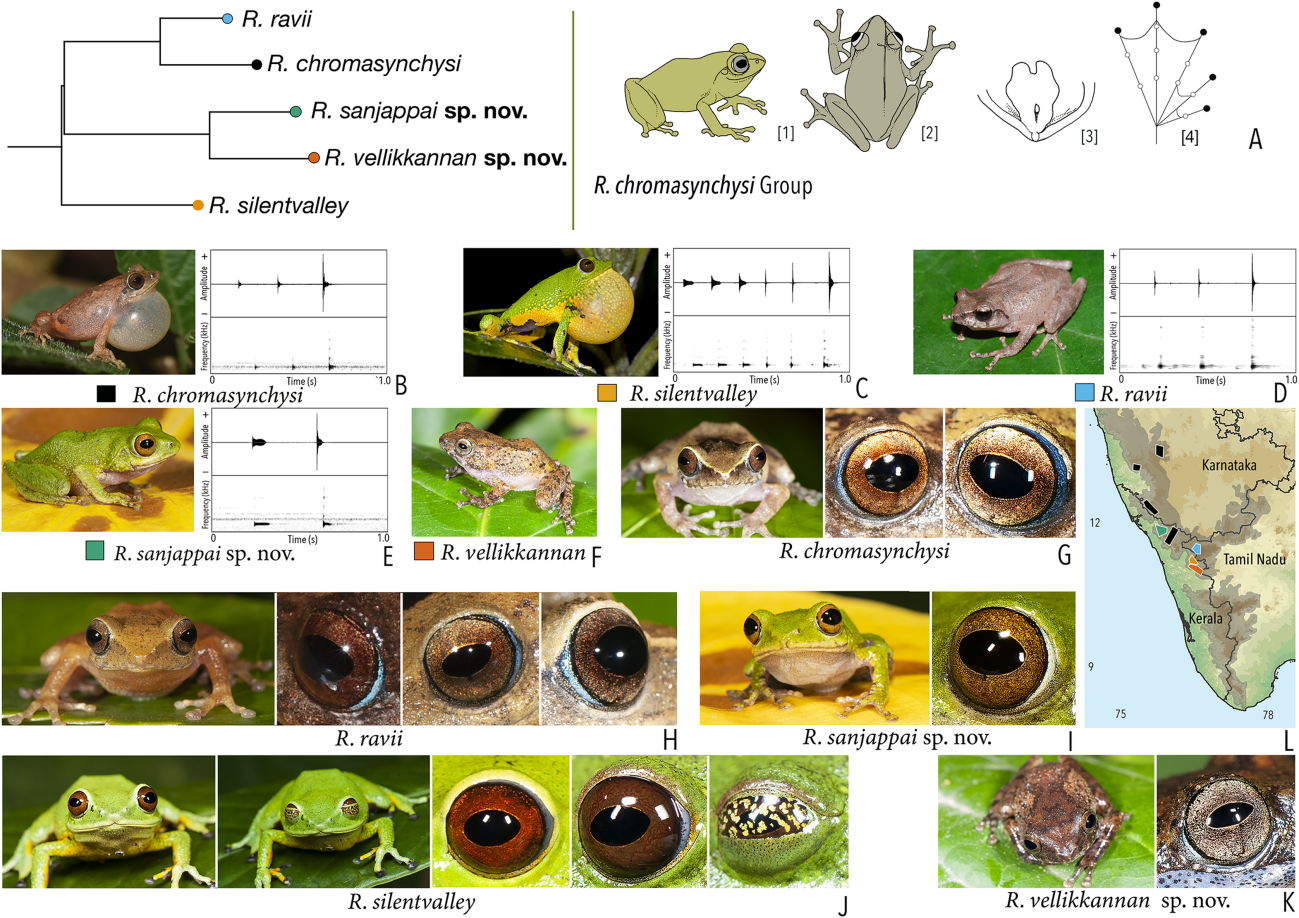
*Raorchestes chromasynchysi* species group. (A) Phylogenetic relationships and major morphological characters for members of the group: (1) adult size range (male SVL 20–35 mm, female SVL 27–37 mm); (2) dorsum with a horny ridge extending from the snout tip to the vent; (3) presence of lingual papillae; (4) foot webbing moderate to large, up to or well beyond the second subarticular tubercle on either side of toe IV. (B and C) Male calls in members of the group: Calling individual, followed by a call oscillogram (above) and spectrogram (below) for *R*. *chromasynchysi* and *R. silentvalley* (1 s section showing a single call for each species). (D and E) Adult male, followed by a call oscillogram (above) and spectrogram (below) for *R. ravii* and *R. sanjappai* sp. nov. (0.1 s section showing a single call for each species). (F) Adult male of *R. vellikkannan* sp. nov. (call not studied). (G–K) Eye colour and pattern in five members of the group. (L) Geographical distribution of members of the group; species range colours on the map correspond to the square colours indicated alongside each species label in (B)–(F).

***Raorchestes chromasynchysi*** group

(Figs. 2 and 16; Tables 1–3)

**Members included.** *Raorchestes chromasynchysi, R. ravii*, *R. sanjappai* sp. nov., *R. silentvalley*, and *R. vellikkannan* sp. nov.

**Group definition. *Phylogenetic*.** The *Raorchestes chromasynchysi* group can be characterised as the most inclusive clade and a Western Ghats radiation that contains *R. chromasynchysi, R. ravii*, *R. sanjappai* sp. nov., *R. silentvalley*, and *R. vellikkannan* sp. nov., but none of the other currently recognised *Raorchestes* species. (Fig. 2). This group is analogous to the Chromasynchysi clade of Vijayakumar et al. (2014). ***Morphological*.** Members of this group can be diagnosed based on the combination of the following characters: small to medium sized shrub frogs (male SVL 20–35 mm, female SVL 27–37 mm); snout sub-elliptical to pointed; dorsum with a mid-dorsal horny ridge extending from the snout tip to the vent; tongue with papillae; foot webbing moderate to large, up to or well beyond the second subarticular tubercle on either side of toe IV (Fig. 16A). ***Eye colouration and pattern***. Members of this group predominantly have a brown, golden brown, reddish-brown, to dark red iris, except silver grey with minute brown speckling in *R. vellikkannan* sp. nov.; iris periphery dark brown to blackish-brown; sclera blue or scarlet blue. One member, *R. silentvalley*, is also known to possess distinct yellowish-green spots and blotches on the palpebral membrane (Figs. 16G–16K; Table 1). ***Acoustic*.** Four members of the group for which calls are studied (*R. chromasynchysi, R. sanjappai* sp. nov., *R. ravii*, and *R. silentvalley*) produce a single type of call. The calls have a pulsatile temporal structure and are not delivered in call groups. Typically, the call duration ranges between 300 and 800 ms and the overall dominant frequency of Type 1 call ranges from 2.2 to 2.7 kHz (Figs. 16B–16E; Table 2). ***Calling height*.** Members of this group usually call from heights of 0.5–6 m on low bushes and shrubs, higher shrubs, and lower to higher tree canopy (Table 2). ***Geographical*.** Members of this group are currently restricted to the Western Ghats regions in Karnataka, Kerala, and Tamil Nadu States (Fig. 16L; Table 3).

**Species level acoustic comparison.** Within the *R. chromasynchysi* group, all the four studied species have a similar call structure. The calls have a long call rise time but lack any significant fall time. *Raorchestes silentvalley* produces calls of relatively longer duration of 716 ms (vs. 499.2 ms in *R. ravii*, 411.2 ms in *R. sanjappai* sp. nov., and 381.4 ms in *R. chromasynchysi*). *R. sanjappai* sp. nov. calls have a relatively slower pulse rate of 2.7 pulses/s (vs. 4.2 pulses/s in *R. ravii*, 5.8 pulses/s in *R. chromasynchysi*, and 7.2 pulses/s in *R. silentvalley*). The dominant frequencies of *R. sanjappai* sp. nov. and *R. silentvalley* calls are similar (2.4 kHz and 2.2 kHz, respectively) and slightly higher than that of *R. silentvalley* (2.5 kHz) and *R. ravii* (2.7 kHz) (Figs. 16B–16E; Table 2).

**Figure 17:**
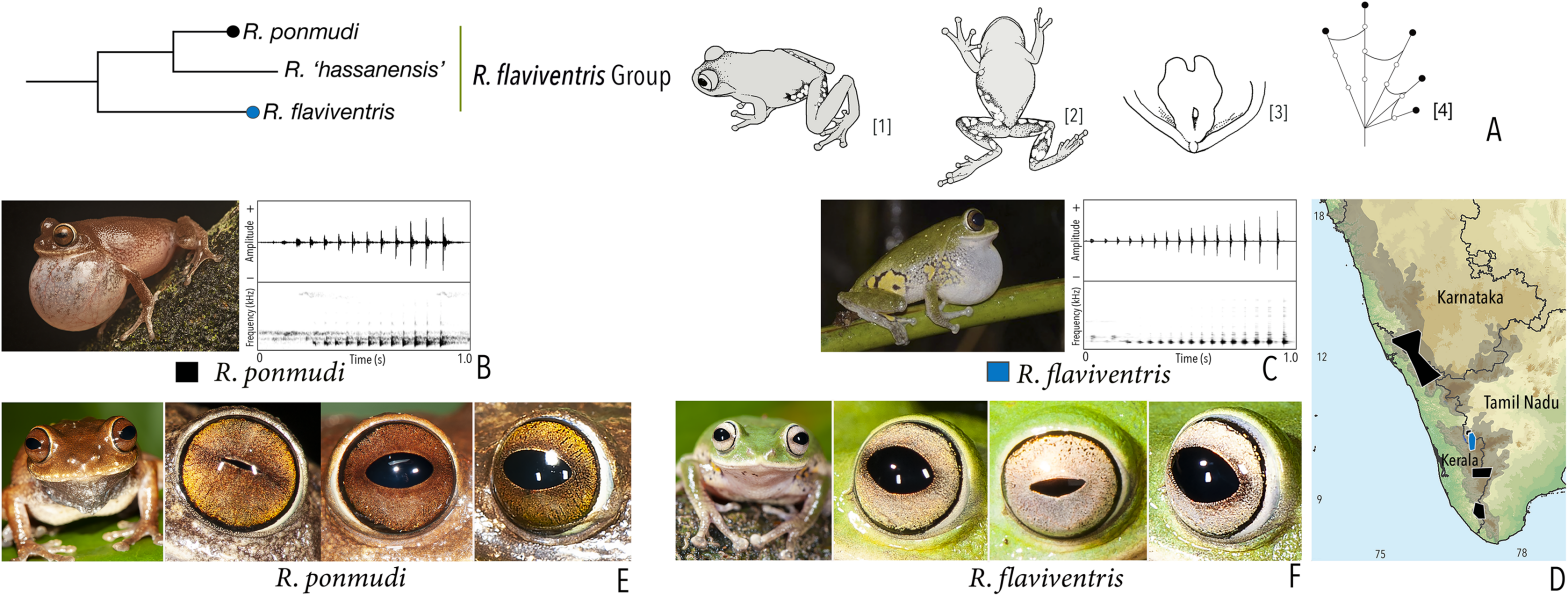
*Raorchestes flaviventris* species group. (A) Phylogenetic relationships and major morphological characters for members of the group: (1) adult size range (male SVL 30–39 mm, female SVL 48–50 mm); (2) snout semi-circular or rounded, and prominent markings or reticulations on groin and thighs; (3) presence of lingual papillae; (4) foot webbing large, not beyond the third subarticular tubercle on toe IV. (B and C) Male calls in members of the group: (B and C) Calling individuals, followed by call oscillograms (above) and spectrograms (below) for *R. ponmudi* and *R. flaviventris* (1 s sections showing a single call for each species). (D) Geographical distribution of members of the group; species range colours on the map correspond to the square colours indicated alongside each species label in (B) and (C). (E and F) Eye colour and pattern in two members of the group.

***Raorchestes flaviventris*** group

(Figs. 2 and 17; Tables 1–3)

**Members included.** *Raorchestes flaviventris* and *R. ponmudi*.

**Group definition. *Phylogenetic*.** The *Raorchestes flaviventris* group can be characterised as the most inclusive clade and a Western Ghats radiation that contains *R. flaviventris* and *R. ponmudi*, but none of the other currently recognised *Raorchestes* species. Another taxon *R*. ‘*hassanensis*’ (currently under the junior objective synonymy of *R. flaviventris*) is suggested as a phylogenetically nested member of this group (Vijayakumar et al., 2014) (Fig. 2). This group is analogous to the Hassanensis clade of Vijayakumar et al. (2014). ***Morphological*.** Members of this group can be diagnosed based on the combination of the following characters: medium to large-sized shrub frogs (male SVL 30–39 mm, female SVL 48–50 mm) and the largest members of the genus; head wider than long; snout semi-circular or rounded; eyes protruding; prominent markings or reticulations on groin and thighs; lingual papillae present; and foot webbing moderate, not beyond the third subarticular tubercle on either side of toe IV (Fig. 17A). ***Eye colouration and pattern***. Iris creamy white, light brown, to golden brown with minute brown speckling; iris periphery dark brown; sclera bluish-grey (Figs. 17E–17F; Table 1). ***Acoustic*.** Two members of the group for which calls are studied (*R. flaviventris* and *R. ponmudi*) produce a single type of call with very similar call structure. The calls have a pulsatile temporal structure and typically range between 400 and 800 ms, have a long call rise time, and lack any significant fall time. The overall dominant frequency of calls of these two species ranges from 1.7 to 1.9 kHz (Figs. 17B and 17C; Table 2). ***Calling height*.** Members of this group usually call from heights of 1–7 m on low bushes and shrubs, higher shrubs, and lower to higher tree canopy (Table 2). ***Geographical*.** Members of this group are currently restricted to the Western Ghats regions in Karnataka, Kerala, and Tamil Nadu States (Fig. 17D; Table 3).

**Species level acoustic comparison.** Within the *R. flaviventris* group, species have a very similar call structure. However, the calls of *R. flaviventris* and *R. ponmudi* vary in duration (712.7 ms and 458.7 ms, respectively) and pulse rate (21.1 pulses/s and 26.4 pulses/s, respectively). The dominant frequency of their calls is similar (1.9 kHz and 1.7 kHz, respectively) (Figs. 17B and 17C; Table 2).

**Figure 18:**
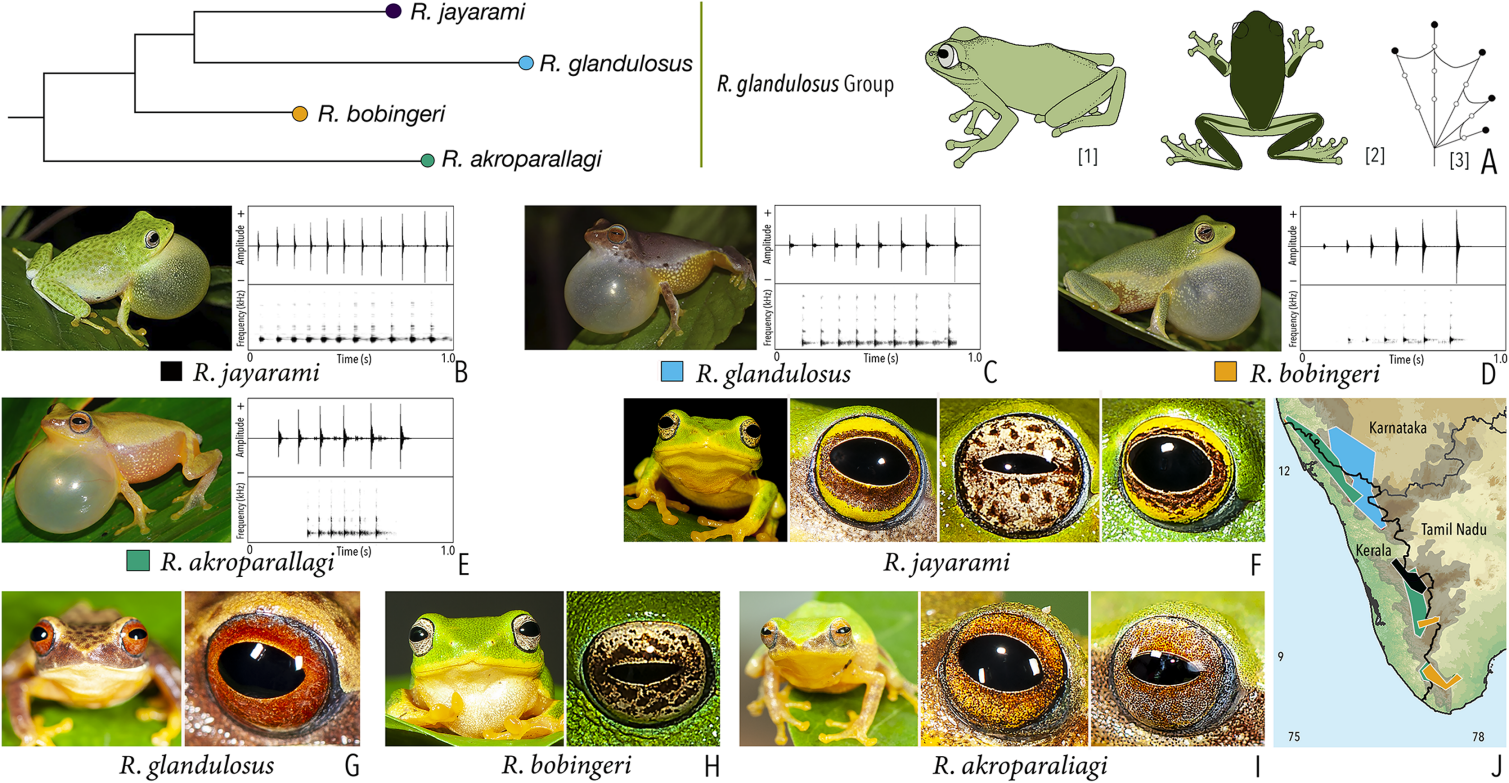
*Raorchestes glandulosus* species group. (A) Phylogenetic relationships and major morphological characters for members of the group: (1) adult size range (male SVL 19–30 mm, female SVL 25–33 mm) and predominantly green shrub frogs; (2) dorsal colouration not completely extending on to the limbs and lateral surfaces; (3) foot webbing moderate, not beyond the third subarticular tubercle on either side of toe IV. (B–E) Male calls in members of the group: (B, C, D and E) Adult males, followed by call oscillograms (above) and spectrograms (below) for *R. jayarami*, *R. glandulosus*, *R. bobingeri*, and *R. akroparallagi* (1 s sections showing a single call for each species). (F–I) Eye colour and pattern in four members of the group. (J) Geographical distribution of members of the group; species range colours on the map correspond to the square colours indicated alongside each species label in (B)–(E).

***Raorchestes glandulosus*** group

(Figs. 2 and 18; Tables 1–3)

**Members included.** *Raorchestes akroparallagi*, *R. bobingeri*, *R. glandulosus*, and *R. jayarami*.

**Group definition. *Phylogenetic*.** The *Raorchestes glandulosus* group can be characterised as the most inclusive clade and a Western Ghats radiation that contains *R. akroparallagi*, *R. bobingeri*, *R. glandulosus*, and *R. jayarami*, but none of the other currently recognised *Raorchestes* species. (Fig. 2). This group is analogous to the Glandulosus clade of Vijayakumar et al. (2014). ***Morphological*.** Members of this group can be diagnosed based on the combination of the following characters: small to large-sized predominantly green shrub frogs (male SVL 19–30 mm, female SVL 25–33 mm); dorsal colouration not completely extending on to the limbs; loreal region vertical; foot webbing moderate, not extending beyond the third subarticular tubercle on either side of toe IV (Fig. 18A). ***Eye colouration and pattern*.** Members of this group have two distinct iris colours and patterns: bright to dark red, reddish-brown, or light brown with golden speckles in *R. glandulosus* and *R. akroparallagi*; and bright yellow, greyish-yellow, or light yellow with an inner reddish-brown or brown continuous ring or irregular spots/reticulations in *R. bobingeri* and *R. jayarami*; iris periphery brownish-black (scarlet blue in subadults of *R. glandulosus*); sclera light blue (Figs. 18F–18I; Table 1). ***Acoustic*.** All members of this group produce a single type of call with very similar structure; the calls are pulsatile and typically range between 500 and 900 ms; calls typically have a long rise time and lack any significant fall time; the overall dominant frequency ranges from 2.7 to 3.6 kHz (Figs. 18B–18E; Table 2). ***Calling height*.** Members of this group usually call from heights of 1–8 m on low bushes and shrubs, higher shrubs, and lower to higher tree canopy (Table 2). ***Geographical*.** This group is currently restricted to the Western Ghats regions in the States of Karnataka, Kerala, and Tamil Nadu (Fig. 18J; Table 3).

**Species level acoustic comparison.** Within the *R. glandulosus* group, the calls of the species show very similar call structure and differ primarily in their pulse rate, which ranges between 10.9 and 13.7 pulses/second. *R. jayarami*, shows the longest call (813.3 ms) with a higher number of pulses (11 pulses) compared to *R. glandulosus* (609.6 ms, eight pulses), *R. akroparallagi* (445.6 ms, six pulses), and *R. bobingeri* (565.6 ms, six pulses). The dominant frequencies of the calls are similar for *R. jayarami* and *R. glandulosus* (2.7 kHz and 2.9 kHz, respectively), while that of *R. akroparallagi* and *R. bobingeri* are similar to each other but relatively higher (3.4 kHz and 3.6 kHz, respectively) (Figs. 18B–18E; Table 2).

**Figure 19:**
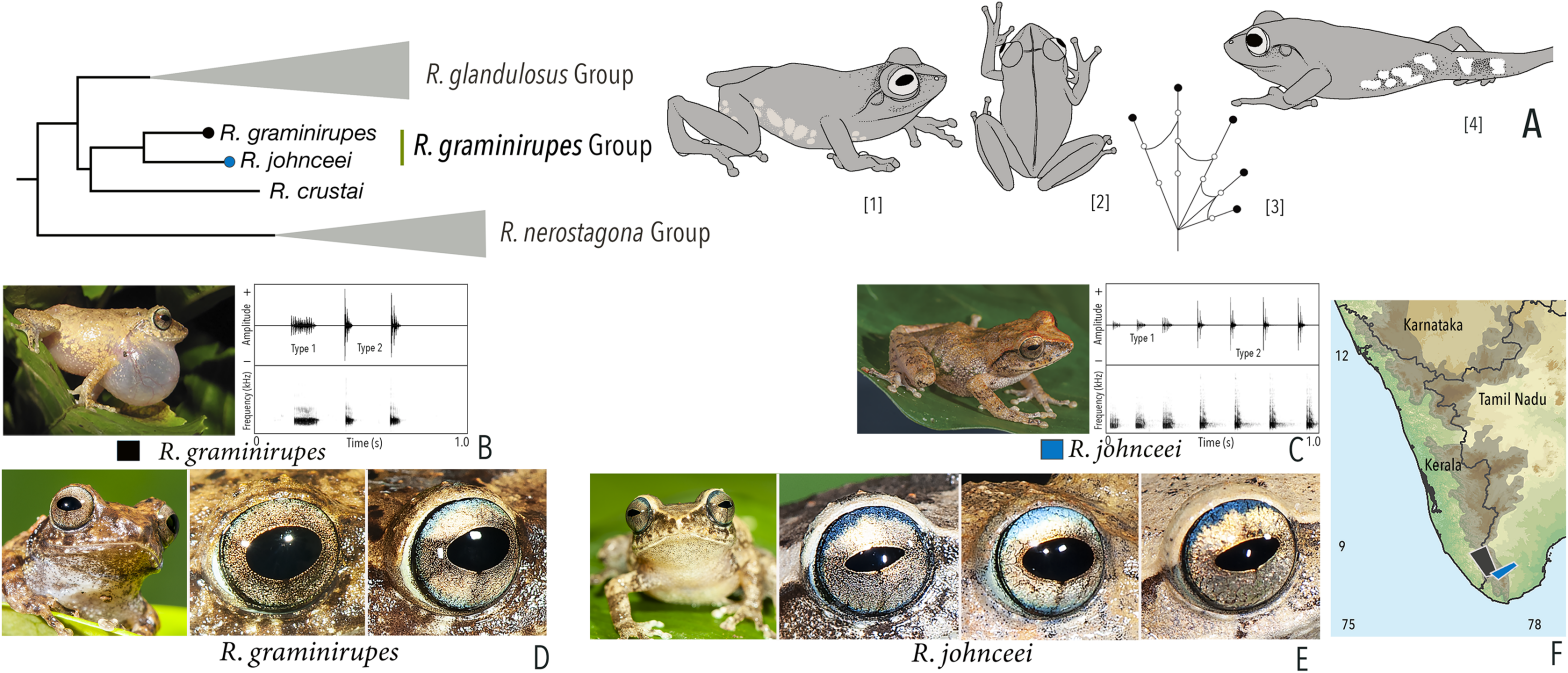
*Raorchestes graminirupes* species group. (A) Phylogenetic relationships and major morphological characters for members of the group: (1) adult size range (male SVL 21–30 mm, female SVL 28–42 mm) and lateral abdominal surfaces green or light brown with yellow or light brown blotches; (2) triangular horny ridge present between the eyes, and a longitudinal mid-dorsal ridge extending from the snout to almost the vent; (3) groin and thighs green or light brown with yellow or light brown blotches; (4) foot webbing moderate, below the third subarticular tubercle on either side of toe IV. (B and C) Male calls in members of the group: (B) Calling individual, followed by a call oscillogram (above) and spectrogram (below) for *R*. *graminirupes* (1 s section showing a single Type 1 call and two Type 2 calls, delivered in group). (C) Adult male, followed by a call oscillogram (above) and spectrogram (below) for *R. johnceei* (1 s section showing three Type 1 calls and four Type 2 calls, delivered in group). (D and E) Eye colour and pattern in two members of the group. (F) Geographical distribution of members of the group; species range colours on the map correspond to the square colours indicated alongside each species label in (B) and (C).

***Raorchestes graminirupes*** group

(Figs. 2 and 19; Tables 1–3)

**Members included.** *Raorchestes graminirupes* and *R. johnceei*.

**Group definition. *Phylogenetic*.** The *Raorchestes graminirupes* group can be characterised as the most inclusive clade and a Western Ghats radiation that contains *R. graminirupes* and *R. johnceei*, but none of the other currently recognised *Raorchestes* species. (Figs. 2 and 19A). This group is analogous to the Graminirupes clade of Vijayakumar et al. (2014). ***Morphological*.** Members of this group can be diagnosed based on the combination of the following characters: small to large-sized adults (male SVL 21–30 mm, female SVL 28–42 mm); pointed snout; tympanum rather indistinct, strong tympanic fold; triangular horny ridge present between the eyes, and a longitudinal mid-dorsal ridge extending from the snout tip to almost the vent; and lateral abdominal surfaces, groin, and anterior thighs green or light brown with yellow or light brown blotches; foot webbing moderate, below the third subarticular tubercle on either side of toe IV (Fig. 19A). ***Eye colouration and pattern*.** Members of this group have a distinct greyish-brown iris with dense metallic silver or light brown speckles; iris periphery black with a scarlet blue inner ring (blue ring more extended in *R. johnceei* compared to *R. graminirupes*); sclera light silvery blue. (Figs. 19D and 19E; Table 1). ***Acoustic*.** Both members of the group produce two types of call; both the call types have a pulsatile temporal structure, are delivered in call groups, and have a fixed call order (Figs. 19B and 19C; Table 2). ***Calling height*.** Members of this group usually call from ground level, low bushes and shrubs, higher shrubs, lower and higher tree canopy up to 8 m high (Table 2). ***Geographical*.** This group is currently restricted to the south of Palghat gap in the Western Ghats, in Kerala and Tamil Nadu States (Fig. 19F; Table 3).

**Species level acoustic comparison.** Within the *R. graminirupes* group, the calls of the two species differ in their temporal as well as spectral properties. The Type 1 calls of *R. graminirupes* are longer (91.3 ms) and have more pulses (18 pulses) that are delivered at a rate of 222.5 pulses/s compared to the calls of *R. johnceei*, which are shorter (66.3 ms) and have fewer pulses (eight pulses) produced at a much slower rate (114.8 pules/s). The dominant frequency is higher in *R. graminirupes* compared with that in *R. johnceei* (2.7 kHz and 2.1 kHz, respectively) (Figs. 19B and 19C; Table 2).

**Figure 20:**
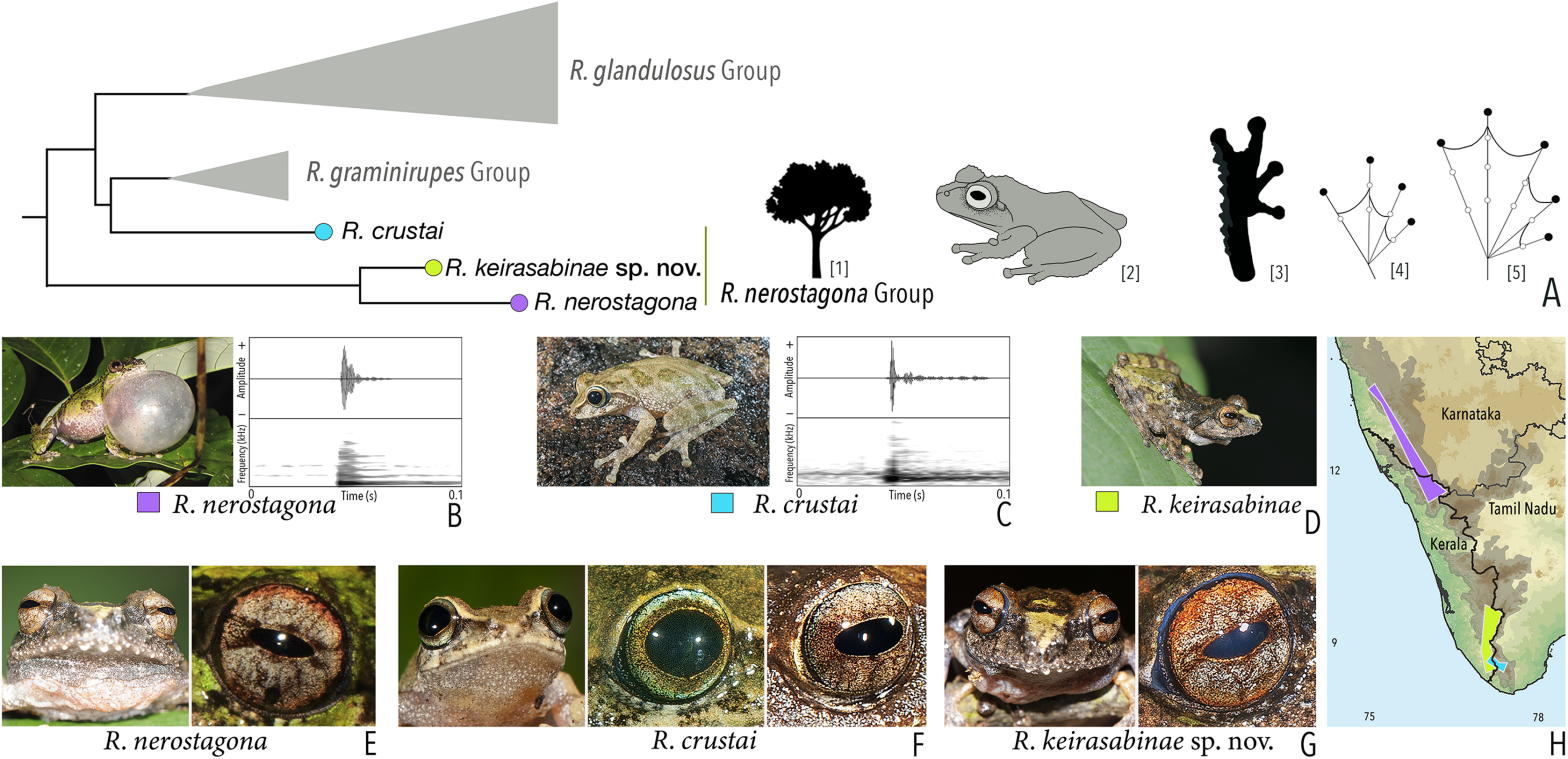
*Raorchestes nerostagona* species group. (A) Phylogenetic relationships and major diagnostic characters for members of the group: (1) predominantly canopy dwelling species, (2) adult size range (male SVL 25–35 mm; female SVL 43 mm); (3) presence of weakly to well-developed dermal fringe along the outer margin of the fore and hind limbs; (4) presence of webbing between fingers (except in *R. crustai*); (5) foot webbing moderate to large, up to or above the second subarticular tubercle on either side of toe IV. (B and C) Male calls in members of the group: (B) Calling individual, followed by a call oscillogram (above) and spectrogram (below) for *R. nerostagona* (0.1 s section showing a single call). (C) Adult male, followed by a call oscillogram (above) and spectrogram (below) for *R. crustai* (0.1 s section showing a single call). (D) Adult male of *R. keirasabinae* sp. nov. (call not studied). (E–G) Eye colour and pattern in three members of the group. (H) Geographical distribution of members of the group; species range colours on map correspond to the square colours indicated alongside each species label in (B)–(D).

***Raorchestes nerostagona*** group

(Figs. 2 and 20; Tables 1–3)

**Members included.** *Raorchestes keirasabinae* sp. nov., *R. nerostagona*, and provisionally *R. crustai*.

**Group definition. *Phylogenetic*.** The *Raorchestes nerostagona* group can be characterised as the most inclusive clade and a Western Ghats radiation that contains *Raorchestes keirasabinae* sp. nov. and *R. nerostagona* (Fig. 2), analogous to the Nerostagona clade of Vijayakumar et al. (2014). Another species, *R. crustai*, that shows an unresolved relationship within the clade comprised of the *R. graminirupes* group, the *R. glandulosus* group, and the *R. nerostagona* group is provisionally included in the *R. nerostagona* group. ***Morphological*.** Members of this group can be diagnosed based on the combination of the following characters: medium to large-sized adults (male SVL 25–35 mm, female SVL 43 mm); presence or absence of webbing between fingers (except in *R. crustai*); presence of weakly to well-developed dermal fringe along the outer margin of the fore and hind limbs; and foot webbing moderate to large, up to or above the second subarticular tubercle on either side of toe IV (Fig. 20A). ***Eye colouration and pattern*.** Two members of the group *Raorchestes keirasabinae* sp. nov. and *R. nerostagona* have a distinct reddish-grey iris with faint or prominent horizontal brown band, iris periphery dark brown, and sclera scarlet blue; however, the iris colour and pattern of the provisionally placed member *R. crustai* is more closely related to that in *Raorchestes graminirupes* group, due to its light greyish-brown colour, iris periphery black with an inner scarlet blue ring, and sclera light silvery blue (Figs. 20E–20G; Table 1). ***Acoustic*.** All members of this group produce a single type of call, with similar call structure. The call of two of the studied species (*R. nerostagona* and *R. crustai*) consists of a single pulse with a short rise time followed by a relatively longer fall time (Figs. 20B and 20C; Table 2). ***Calling height*.** Members of this group can be found calling from high shrubs to the lower and higher tree canopy at heights of up to 40 m (Table 2). ***Geographical*.** This group is currently known only from the Western Ghats regions in the States of Karnataka, Kerala, and Tamil Nadu (Fig. 20H; Table 3). ***Habitat preference*.** Members of this group are predominantly canopy dwelling species found in forested areas.

**Species level acoustic comparison.** Within the *R. nerostagona* group, the call of *R. nerostagona* is longer (23.3 ms) compared to the call of *R. crustai* (13.3 ms). The dominant frequency of the calls of these two species are similar (2.0 kHz and 2.2 kHz, respectively) (Figs. 20B and 20C; Table 2).

**Figure 21:**
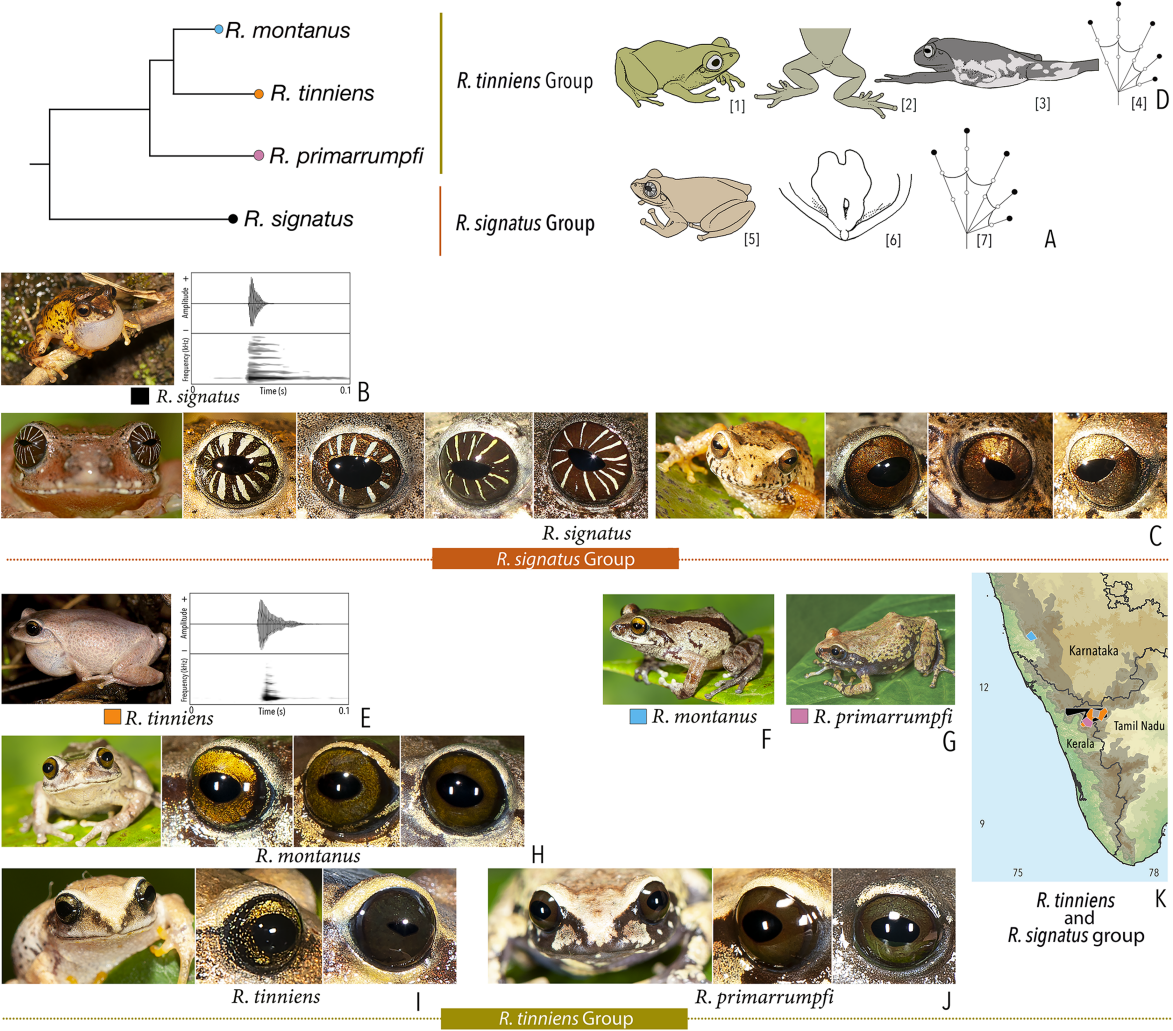
*Raorchestes signatus* species group and *R. tinniens* species group. (A) Phylogenetic relationships and major morphological characters for the sole recognised member of the *Raorchestes signatus* species group: (1) adult size range (male SVL 24–29 mm, female SVL 34–42 mm); (2) presence of lingual papillae; (3) foot webbing small, not beyond the second subarticular tubercle on either side of toe IV. (B) Calling male, followed by a call oscillogram (above) and spectrogram (below) for *R. signatus* (0.1 s section showing a single call). (C) Eye colour and pattern in *R*. *signatus*. (D) Phylogenetic relationships and major morphological characters for members of the *Raorchestes tinniens* species group: (1) adult size range (male SVL 18–29 mm, female SVL 27–39 mm), and predominantly ground-dwelling species found in mountain grasslands or shola forests; (2) relatively short legs; (3) dorsum with a distinct metallic tinge in all colour forms, and often with mosaic pattern on lateral surfaces and groin; (4) foot webbing small, not beyond the second subarticular tubercle on either side of toe IV. (E) Calling male, followed by a call oscillogram (above) and spectrogram (below) for *R. tinniens* (0.1 s section showing a single call). (F and G) Adult males of *R. montanus* and *R. primarrumpfi* (calls not studied). (H–J) Eye colour and pattern in three members of the group. (K) Geographical distribution of members of the *R*. *signatus* group and *R. tinniens* group; species range colours on the map correspond to the square colours indicated alongside each species label in (B) and (E)–(G). Photo of front view of head (C) by Rajkumar K. P.

***Raorchestes signatus*** group

(Figs. 2, 21A–21C and 21K; Tables 1–3)

**Members included.** *Raorchestes signatus* and *R. thodai*.

**Group definition. *Phylogenetic*.** The *Raorchestes signatus* group can be characterised as the most inclusive clade and a Western Ghats radiation that contains *R. signatus* and another closely related taxon *R. thodai* with doubtful taxonomic status, but none of the other currently recognised *Raorchestes* species (Fig. 2). This group is analogous to the Signatus clade of Vijayakumar et al. (2014). This group is likely to contain additional undescribed species (SG and SDB unpublished data). ***Morphological*.** This group can be diagnosed based on the combination of the following characters: small to large-sized frogs (male SVL 24.0–29.0 mm, female SVL 34.0–42.0 mm); dorsal colouration highly variable but usually with an ‘X’ mark; tongue with lingual papillae; and foot webbing small, not extending beyond the second subarticular tubercle on either side of toe IV (Fig. 21A). ***Eye colouration and pattern*.** Iris brown, dark brown or reddish-brown, with or without silver white or golden radiating lines and golden speckling; iris periphery without prominent ring; and sclera greyish-brown (Fig. 21C; Table 1). ***Acoustic*.** The sole studied member of the group produces a single type of non-pulsatile call and delivered at uniform intervals within call groups. Typically, the calls have a short rise time and a relatively longer fall time. The overall dominant frequency of the calls of the species is around 2.1 kHz (Fig. 21B; Table 2). ***Calling height*.** Members of the group calls from ground level to low bushes and shrubs, higher shrubs, lower and higher tree canopy up to 10 m high (Table 2). ***Geographical*.** This group is currently restricted to the Western Ghats regions in Kerala and Tamil Nadu States (Fig. 21K; Table 3).

***Raorchestes tinniens*** group

(Figs. 2 and 21D–21K; Tables 1–3)

**Members included.** *Raorchestes montanus, R. primarrumpfi*, and *R. tinniens*.

**Group definition. *Phylogenetic*.** The *Raorchestes tinniens* group can be characterised as the most inclusive clade and a Western Ghats radiation that contains *R. montanus, R. primarrumpfi*, and *R. tinniens*, but none of the other currently recognised *Raorchestes* species. (Fig. 2). This group is analogous to the Tinniens clade of Vijayakumar et al. (2014). ***Morphological*.** Members of this group can be diagnosed based on the combination of the following characters: small to large-sized frogs (male SVL 18–29 mm, female SVL 27–39 mm); dorsum with a distinct metallic tinge in all colour forms and often with mosaic pattern on lateral surfaces and groin; relatively short legs; foot webbing small, not extending up to the second subarticular tubercle on either side of toe IV; and tongue with lingual papillae (Fig. 21D). ***Eye colouration and pattern*.** Members of the group have a dark brown, golden brown, or greyish-brown iris, occasionally with golden speckling; iris periphery black; sclera ash grey. Vijayakumar et al. (2014) noted the iris in *R. primarrumpfi* as having a dark maroon lower part and the upper half speckled with iridescent golden and silver. However, the same was not observed in any sample of this species from its type locality and vicinities in our study (Figs. 21H–21J; Table 1). ***Acoustic*.** A single member of the group for which calls are studied (*R. tinniens*) produces a single type of non-pulsatile call that is organised into call groups and separated by uniform intervals. Typically, the calls have a short rise time and a relatively longer fall time. The overall dominant frequency of the calls is around 2.6 kHz (Fig. 21E; Table 2). ***Calling height*.** Members of this group usually call from ground level to low bushes and shrubs up to 1.5 m high (Table 2). ***Geographical*.** Members of this group are currently restricted to the Western Ghats regions in Karnataka, Kerala, and Tamil Nadu States (Fig. 21K; Table 3). ***Habitat preference*.** Members of this group are predominantly ground-dwelling species inhabiting mountain grasslands or shola forests.

**Figure 22:**
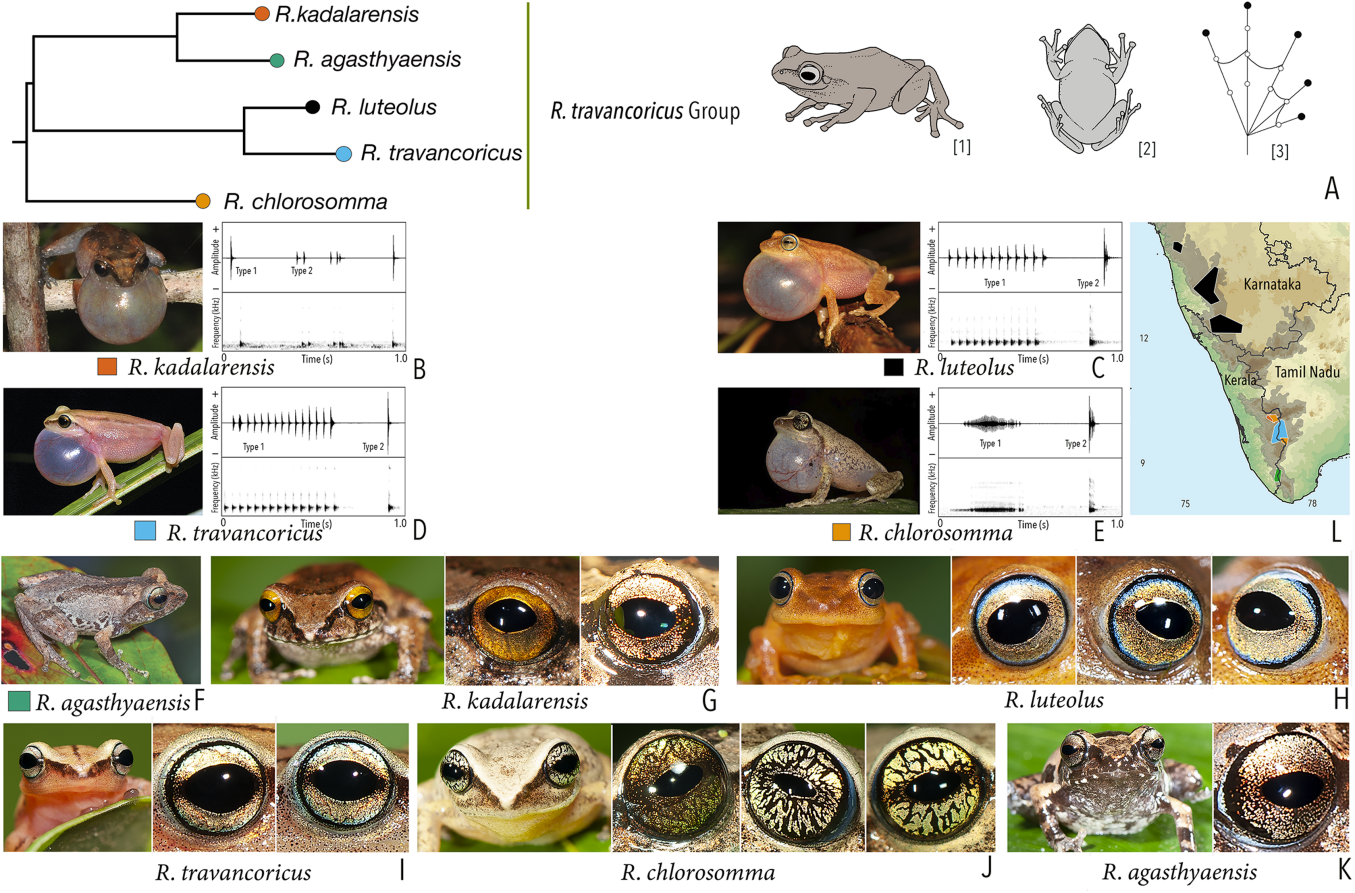
*Raorchestes travancoricus* species group. (A) Phylogenetic relationships and major morphological characters for members of the group: (1) adult size range (male SVL 16–30 mm, female SVL 21–39 mm); (2) snout sub-elliptical to pointed; (3) foot webbing rudimentary to small, not beyond the second subarticular tubercle on either side of toe IV. (B–E) Male calls in members of the group: (B, C, D and E) Calling individuals, followed by call oscillograms (above) and spectrograms (below) for *R. kadalarensis* (1 s section showing two Type 1 calls and two Type 2 calls, delivered in group), *R. luteolus*, *R. travancoricus*, and *R. chlorosomma* (1 s sections showing a single Type 1 call and a single Type 2 call for each of the latter three species, calls delivered in groups). (F) Adult male of *R*. *agasthyaensis* (call not studied). (G–K) Eye colour and pattern in five members of the group. (L) Geographical distribution of members of the group; species range colours on the map correspond to the square colours indicated alongside each species label in (B)–(F). Photos of calling males in (C) and (D) by Seshadri K. S. and Manoj P., respectively.

***Raorchestes travancoricus*** group

(Figs. 2 and 22; Tables 1–3)

**Members included.** *Raorchestes agasthyaensis, R. chlorosomma, R. kadalarensis, R. luteolus*, and *R. travancoricus*.

**Group definition. *Phylogenetic*.** The *Raorchestes travancoricus* group can be characterised as the most inclusive clade and a Western Ghats radiation that contains *R. agasthyaensis, R. chlorosomma, R. kadalarensis, R. luteolus, and R. travancoricus*, but none of the other currently recognised *Raorchestes* species (Fig. 2). This group is analogous to the Travancoricus clade of Vijayakumar et al. (2014). ***Morphological*.** Members of this group can be diagnosed based on the combination of the following characters: small to medium-sized adults (male SVL 16–30 mm, female SVL 21–39 mm); snout sub-elliptical to pointed; tympanum smaller than eyes; foot webbing rudimentary to small, not extending beyond the second subarticular on either side of toe IV (Fig. 22A). ***Eye colouration and pattern*.** Members of this group have variable iris colour and patterns: *R. luteolus* and *R. travancoricus* have golden yellow or light grey iris with brown speckling with a cobalt blue outsider ring, iris periphery black or bluish**-**black, and indistinct sclera; *R. agasthyaensis* and *R. kadalarensis* have dark brown iris with dense golden speckling, iris horizontally divided into light upper and dark lower halves, iris periphery dark brown, and sclera ash grey; or a distinctly metallic greyish-green or greenish-yellow iris with dark brown reticulations, iris periphery dark brown, and sclera scarlet blue in *R. chlorosomma* (Figs. 22G–22K; Table 1). ***Acoustic*.** Four members of the group for which calls were studied (*R. chlorosomma, R. kadalarensis*, *R. luteolus*, and *R. travancoricus*) produce two types of call. The Type 1 call in three species (*R. chlorosomma, R. luteolus*, and *R. travancoricus*) has a pulsatile temporal structure, whereas *R. kadalarensis* call comprises of a single pulse. On the other hand, the Type 2 call of two species (*R. travancoricus* and *R. luteolus*) consists of a single pulse, and that of *R. chlorosomma* and *R. kadalarensis* has a pulsatile temporal structure. The overall dominant frequency of Type 1 call of these species ranges from 2.2 to 3.4 kHz (Figs. 22B–22E; Table 2). ***Calling height*.** Members of this group usually call from heights of 0.5–4 m on low bushes and shrubs to high shrubs (Table 2). ***Geographical*.** Members of this group are widely distributed in the Western Ghats regions in Karnataka, Kerala, and Tamil Nadu States (Fig. 22L; Table 3).

**Species level acoustic comparison.** Within the *R. travancoricus* group, *R. travancoricus* and *R. luteolus* produce calls with very similar structures, while the calls of *R. chlorosomma* and *R. kadalarensis* are different from other members of the group. Type 1 calls of *R. travancoricus* and *R. luteolus* have similar temporal properties with relatively longer call duration, 474.5 ms and 390.5 ms, respectively (vs. 246.2 ms in *R. chlorosomma* and 15.6 ms in *R. kadalarensis*), and much slower pulse rate, 33.1 pulse/s and 31.9 pulse/s, respectively (vs. 195.8 pulses/s in *R. chlorosomma*). The dominant frequency of Type 1 calls of *R. travancoricus* and *R. kadalarensis* are similar (3.3 kHz and 3.4 kHz, respectively) and both are higher in frequency than in the Type 1 calls of *R. luteolus* and *R. chlorosomma*, which are more similar to each other (2.5 kHz and 2.2 kHz, respectively). The non-pulsatile Type 2 calls of *R. travancoricus* and *R. luteolus* are relatively shorter, 17.2 ms and 27.2 ms, respectively, compared to the pulsatile Type 2 calls of *R. chlorosomma* (30.2 ms) and *R. kadalarensis* (52.6 ms). The dominant frequency of both Type 1 and Type 2 calls are similar in each species (Figs. 22B–22E; Table 2).

**Figure 23:**
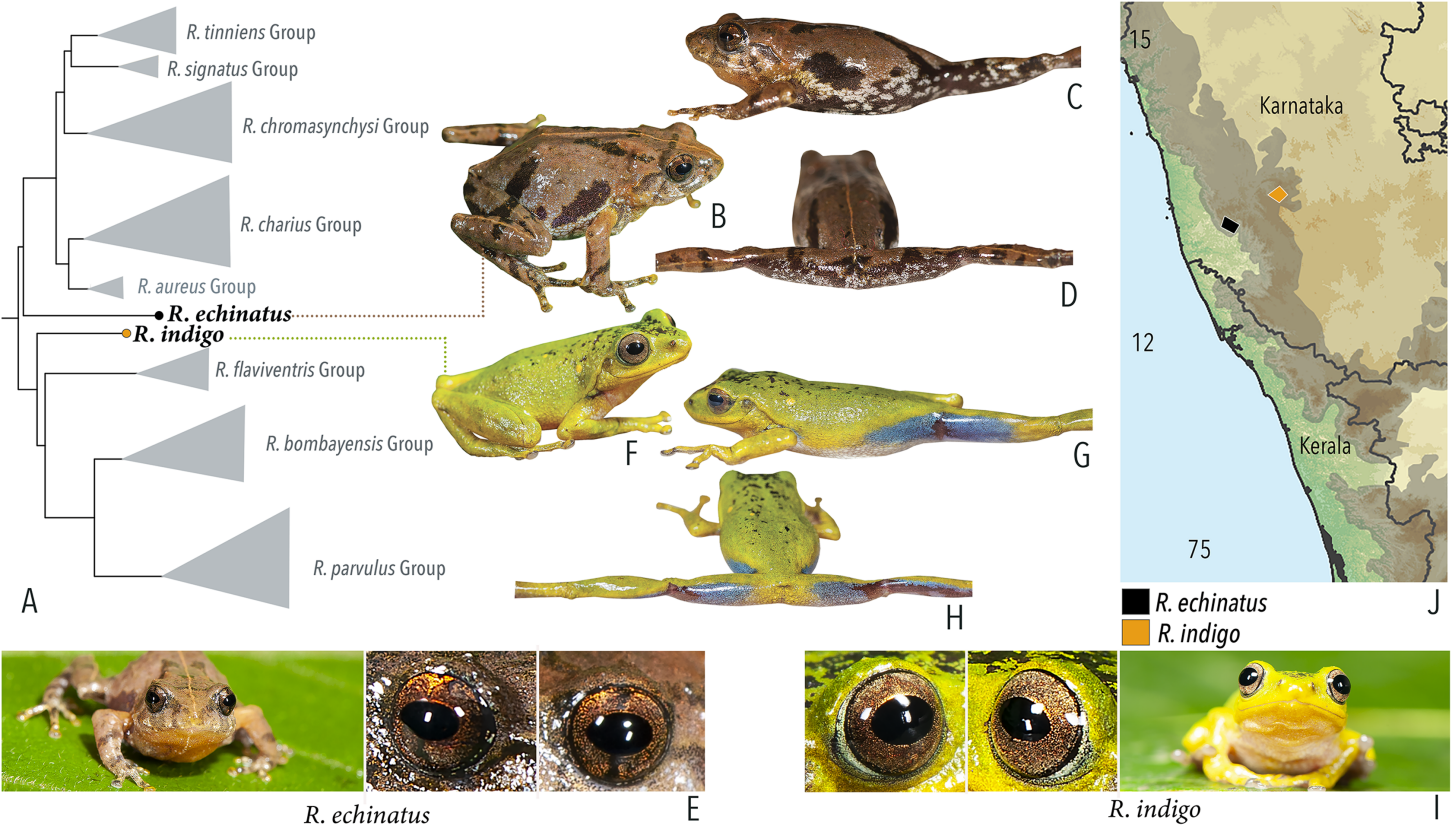
Ungrouped species. (A) Phylogenetic position of two ungrouped species, *Raorchestes echinatus* and *R. indigo*. (B–E) *R. echinatus*: (B) Dorsolateral view.(C) Lateral view. (D) Posterior view of thighs. (E) Eye colour and pattern. (F–I) *R. indigo*: (F) Dorsolateral view. (G) Lateral view. (H) Posterior view of thighs. (I) Eye colour and pattern. (J) Geographical distribution of *R*. *echinatus* and *R. indigo*.

**Ungrouped species:** *Raorchestes echinatus* and *R. indigo*

(Figs. 2 and 23; Tables 1–3)

Two species, *Raorchestes echinatus* and *R. indigo*, cannot be unambiguously assigned to any of the defined species groups due to their unresolved phylogenetic relationship (Vijayakumar et al., 2014; Fig. 2, present study) and lack of clearly shared morphological characters with any other groups. We could also not study the acoustic characters of these species. Vijayakumar et al. (2014) discussed the superficial morphological similarities between *R. echinatus* and *R. tuberohumerus* largely based on skin colouration and texture, and certain body dimensions. We further note that *R. echinatus* could be more closely related to members of the *R. bombayensis* group due to comparable adult size, presence of mid-dorsal lines, spinular dorsum, and mottled ventral skin. On the other hand, *R. indigo* shows resemblance with *R. beddomii*, a member of the *R. beddomii* group, due to comparable adult size, green dorsal colour that extends on to the dorsal surfaces of limbs, loreal and tympanic regions, and occasional presence of blue colouration on groin, armpits, and thighs (Biju & Bossuyt, 2009). However, we currently refrain from assigning *R. echinatus* and *R. indigo* to any of the defined species groups (Fig. 23), with their grouping pending to be resolved based on further evidence (such as acoustic, ecological, reproductive, or behavioral aspects) or possible future discoveries of new and closely related taxa.

## Discussion

The genus *Raorchestes* accounts for nearly one-fourth of the currently known frog diversity of the Western Ghats. Taxonomic studies on such a large radiation that lacks clear morphological synapomorphies among closely related species poses several challenges, particularly with a large number of new species accumulating within a short span of time. Despite already being one of the most actively researched groups of anurans of the region for nearly two decades (e.g., Biju & Bossuyt, 2005a, 2006, 2009; Biju et al., 2010; Zachariah et al., 2011, 2016; Vijayakumar et al., 2014, 2016), the known shrub frog diversity continues to rise. This is not surprising since groups where significant efforts have been made to resolve existing taxonomic confusions—other examples such as *Nyctibatrachus* (Biju et al., 2011; Van Bocxlaer et al., 2012; Garg et al., 2017), *Indirana* (Dahanukar et al., 2016; Garg & Biju, 2016), and *Minervarya* (Dubois, Ohler & Biju, 2001; Dinesh et al., 2015; Garg & Biju, 2017; Raj et al., 2018)—have also experienced renewed interest from researchers, consequently increasing the rate of new discoveries. On this backdrop, our discovery of another five new *Raorchestes* species indicates that much work remains to be done. Recent studies by Vijayakumar et al. (2014, 2016) have also shown the presence of undescribed diversity in this genus based on phylogenetic evidence that remains to be investigated further; however much of their available molecular data lacks the associated geographical or morphological information, with the corresponding species identity remaining largely unverified. Further studies are necessary to clarify the taxonomic status of all the currently known shrub frog populations and to better understand the patterns of diversification and distribution of this group in the Western Ghats.

Current knowledge about the distribution of *Raorchestes* frogs in the Western Ghats shows that half (28 species) of the known diversity (56 recognised species) is endemic to the southern Western Ghats, that is broadly considered as regions south of Palghat gap. In a phylogenetic perspective as well, the Western Ghats shrub frogs comprise two large radiations, (1) the Southern clade with ancestral range in the southern Western Ghats and (2) the Northern clade with ancestral range north of Palghat gap (Vijayakumar et al., 2016; Fig. 2 in present study). It is the northern clade that gives rise to radiations dispersed outside the Western Ghats, into Central India and the Eastern Ghats, that is the *Raorchestes bombayensis* species group (Figs. 2 and 12; Table 3), further into Northeast India and the remaining range of the genus in South, Southeast, and East Asia, that is the *Raorchestes parvulus* species group (Fig. 2). The geographical range of the *Raorchestes charius* group (northern clade) also extends from the Western Ghats to the Eastern Ghats. On the other hand, the southern clade represents ancient lineages restricted to the southern Western Ghats regions of Kerala and Tamil Nadu States, suggesting these hill ranges to be a reservoir of remnant and endemic anuran fauna (e.g., Biju & Bossuyt, 2003; Roelants, Jiang & Bossuyt, 2004). Our discoveries of the five new *Raorchestes* species described in this study were made from forested areas in the State of Kerala, encompassing the hill ranges of Agasthyamalai, Cardamom (south of Palghat gap), and Nilgiris, Siruvani, Wayand (North of Palghat gap). Amphibians in these regions are known to be facing increasing anthropogenic threats (Biju et al., 2008; Nair et al., 2011; Garg et al., 2017; Thorpe et al., 2018) and all the new species are found either outside protected areas or in fragmented primary forest patches and highly disturbed secondary forest areas. The new species will therefore require immediate assessment of threats to the known populations and habitats, and their conservation status.

One of the major aims of our study was to comprehensively infer the systematic relationships between *Raorchestes* species and define species groups using integrative approaches in order to facilitate a better working taxonomy for this large and morphologically challenging radiation of rhacophorid frogs. We define 16 species groups, primarily delimited based on phylogeny (Vijayakumar et al., 2014; present study), that are additionally diagnosable based on morphology, acoustics, and as well as geographical parameters. In doing so, we provide novel insights that will further enable proper laboratory as well as field-based identification and documentation of *Raorchestes* frogs in the Western Ghats, and thereby also assist in their conservation.

This is also the first time that bioacoustics studies have been carried out at a large-scale for this genus covering 45 of the now recognised 59 species from Peninsular India, thereby allowing a better understanding of the interspecific differences and possible group-level diagnostic call characters and their usefulness in taxonomy. The current work will pave the way for future acoustic studies on this group, which are required to describe the vocal repertoires of species in detail and understand intraspecific variations (Bee, Suyesh & Biju, 2013a, 2013b). An overall understanding of the basic call structures of members of the various species groups will also promote non-invasive documentation and monitoring of shrub frogs in the Western Ghats. The calling height of frogs also plays an important role in niche segregation (Hödl, 1977; Martins, Almedia & Jorge, 2006; Wells, 2007), hence future studies can investigate the evolutionary processes that are likely to have driven premating and postmating isolation mechanisms in this unique group of direct-developing tree frogs. Future research is also necessary on various understudied aspects of the group members such as reproductive behavior, ecology, and natural history (barring a few studies such as Bossuyt et al., 2001; Krishnamurthy, Gururaja & Manjunatha Reddy, 2002; Biju, 2003; Biju & Bossuyt, 2005a, 2005b, 2009; Biju et al., 2010; Seshadri, Gururaja & Bickford, 2015; Princy & Kannan, 2018), which can further enhance the diagnosability of various species and species groups. Our study also highlights a larger set of morphological characters, including various traits such as eye colour and patterns for group-level diagnosis of *Raorchestes* frogs. This will not only assist future taxonomic research within the Western Ghats but also of the extended radiations of *Raorchestes* frogs in South, Southeast and East Asia, for which no such comparable studies are currently available.

## Supplemental Information

10.7717/peerj.10791/supp-1 Supplemental Information 1 Supplemental tables.

10.7717/peerj.10791/supp-2 Supplemental Information 2 Call of *Raorchestes akroparallagi* (1 sec segment).

10.7717/peerj.10791/supp-3 Supplemental Information 3 Call of *Raorchestes anili* (1 sec segment).

10.7717/peerj.10791/supp-4 Supplemental Information 4 Call of *Raorchestes archeos* (1 sec segment).

10.7717/peerj.10791/supp-5 Supplemental Information 5 Type 1 call of *Raorchestes beddomii* (1 sec segment).

10.7717/peerj.10791/supp-6 Supplemental Information 6 Type 2 call of *Raorchestes beddomii* (1 sec segment).

10.7717/peerj.10791/supp-7 Supplemental Information 7 Call of *Raorchestes blandus* (1 sec segment).

10.7717/peerj.10791/supp-8 Supplemental Information 8 Call of *Raorchestes bobingeri* (1 sec segment).

10.7717/peerj.10791/supp-9 Supplemental Information 9 Call of *Raorchestes bombayensis* (0.1 sec segment).

10.7717/peerj.10791/supp-10 Supplemental Information 10 Call of *Raorchestes chalazodes* (0.1 sec segment).

10.7717/peerj.10791/supp-11 Supplemental Information 11 Call of *Raorchestes charius* (0.1 sec segment).

10.7717/peerj.10791/supp-12 Supplemental Information 12 Call of *Raorchestes chlorosomma* (1 sec segment).

10.7717/peerj.10791/supp-13 Supplemental Information 13 Call of *Raorchestes chotta* (1 sec segment).

10.7717/peerj.10791/supp-14 Supplemental Information 14 Call of *Raorchestes chromasynchysi* (1 sec segment).

10.7717/peerj.10791/supp-15 Supplemental Information 15 Call of *Raorchestes coonoorensis* (0.1 sec segment).

10.7717/peerj.10791/supp-16 Supplemental Information 16 Call of *Raorchestes crustai* (0.1 sec segment).

10.7717/peerj.10791/supp-17 Supplemental Information 17 Call of *Raorchestes drutaahu* sp. nov. (0.1 sec segment).

10.7717/peerj.10791/supp-18 Supplemental Information 18 Call of *Raorchestes dubois* (0.1 sec segment).

10.7717/peerj.10791/supp-19 Supplemental Information 19 Call of *Raorchestes flaviventris* (1 sec segment).

10.7717/peerj.10791/supp-20 Supplemental Information 20 Call of *Raorchestes ghatei* (0.1 sec segment).

10.7717/peerj.10791/supp-21 Supplemental Information 21 Call of *Raorchestes glandulosus* (1 sec segment).

10.7717/peerj.10791/supp-22 Supplemental Information 22 Call of *Raorchestes graminirupes* (1 sec segment).

10.7717/peerj.10791/supp-23 Supplemental Information 23 Call of *Raorchestes griet* (0.1 sec segment).

10.7717/peerj.10791/supp-24 Supplemental Information 24 Call of *Raorchestes honnametti* (0.1 sec segment).

10.7717/peerj.10791/supp-25 Supplemental Information 25 Call of *Raorchestes jayarami* (1 sec segment).

10.7717/peerj.10791/supp-26 Supplemental Information 26 Call of *Raorchestes johnceei* (1 sec segment).

10.7717/peerj.10791/supp-27 Supplemental Information 27 Call of *Raorchestes kadalarensis* (1 sec segment).

10.7717/peerj.10791/supp-28 Supplemental Information 28 Call of *Raorchestes kaikatti* (1 sec segment).

10.7717/peerj.10791/supp-29 Supplemental Information 29 Call of *Raorchestes kakkayamensis* sp. nov. (0.1 sec segment).

10.7717/peerj.10791/supp-30 Supplemental Information 30 Call of *Raorchestes leucolatus* (0.1 sec segment).

10.7717/peerj.10791/supp-31 Supplemental Information 31 Call of *Raorchestes luteolus* (1 sec segment).

10.7717/peerj.10791/supp-32 Supplemental Information 32 Call of *Raorchestes manohari* (0.1 sec segment).

10.7717/peerj.10791/supp-33 Supplemental Information 33 Call of *Raorchestes marki* (0.1 sec segment).

10.7717/peerj.10791/supp-34 Supplemental Information 34 Call of *Raorchestes munnarensis* (1 sec segment).

10.7717/peerj.10791/supp-35 Supplemental Information 35 Call of *Raorchestes nerostagona* (0.1 sec segment).

10.7717/peerj.10791/supp-36 Supplemental Information 36 Call of *Raorchestes ochlandrae* (0.1 sec segment).

10.7717/peerj.10791/supp-37 Supplemental Information 37 Call of *Raorchestes ponmudi* (1 sec segment).

10.7717/peerj.10791/supp-38 Supplemental Information 38 Call of *Raorchestes ravii* (1 sec segment).

10.7717/peerj.10791/supp-39 Supplemental Information 39 Call of *Raorchestes resplendens* (1 sec segment).

10.7717/peerj.10791/supp-40 Supplemental Information 40 Call of *Raorchestes sanjappai* sp. nov. (1 sec segment).

10.7717/peerj.10791/supp-41 Supplemental Information 41 Call of *Raorchestes signatus* (0.1 sec segment).

10.7717/peerj.10791/supp-42 Supplemental Information 42 Call of *Raorchestes silentvalley* (1 sec segment).

10.7717/peerj.10791/supp-43 Supplemental Information 43 Call of *Raorchestes sushili* (1 sec segment).

10.7717/peerj.10791/supp-44 Supplemental Information 44 Call of *Raorchestes tinniens* (0.1 sec segment).

10.7717/peerj.10791/supp-45 Supplemental Information 45 Call of *Raorchestes travancoricus* (1 sec segment).

10.7717/peerj.10791/supp-46 Supplemental Information 46 Call of *Raorchestes tuberohumerus* (0.1 sec segment).

10.7717/peerj.10791/supp-47 Supplemental Information 47 Call of *Raorchestes uthamani* (0.1 sec segment).

10.7717/peerj.10791/supp-48 Supplemental Information 48 Call of *Raorchestes sanctisilvaticus* (0.1 sec segment).

## Abbreviations

BNHS: Bombay Natural History Society, Mumbai
SDBDU: Systematics Lab, University of Delhi, Delhi, India
CESF: Centre for Ecological Sciences—Frogs, Indian Institute of Science, Bangalore, India
HT: Holotype
PT: Paratype
RS: Referred Specimen
WLS: Wildlife Sanctuary
SG: Sonali Garg
RS: Robin Suyesh
SD: Sandeep Das
MAB: Mark A Bee
SDB: S D Biju

## Additional Information and Declarations

### Field Study Permissions

The following information was supplied relating to field study approvals (i.e., approving body and any reference numbers): Fieldwork, including the collection of animals in the field, was conducted with permissions and following guidelines from the responsible authorities in the State Forest Departments (Fieldwork research permits: No.WL12-1830/2009, No.WL10-2606/12, No.WL10-25421/2014, No.67254/2001/WL5; D-22 (8)/Research/4543/2012-13, No. PCCF(WL)/E2/CR/13/2016–17, WL10-43756/2015).

### Data Availability

The following information was supplied regarding data availability: DNA sequences are available at NCBI GenBank: MW020034–MW020035, MW020166–MW020171, and MW023233–MW023244. Type specimens for the new species described in the study are deposited in Bombay Natural History Society, Mumbai, under accession numbers BNHS 6088–6101. Non-type referred specimens are available at Systematics Lab, University of Delhi (SDBDU).

### New Species Registration

The following information was supplied regarding the registration of a newly described species: Publication LSID urn:lsid:zoobank.org:pub:7021B266-C54A-4E64-8645-AACCBFBB1A72 *Raorchestes drutaahu* sp. nov. LSID: urn:lsid:zoobank.org:act:4B07924D-80B3-468B-8C35-C3A8C3001E23 *Raorchestes kakkayamensis* sp. nov. LSID: urn:lsid:zoobank.org:act:BD7A17C6-A1E2-421A-B0E8-2336931CD63F *Raorchestes keirasabinae* sp. nov. LSID: urn:lsid:zoobank.org:act:39BCB0CE-166E-4C90-AE1E-564BD626ABA9 *Raorchestes sanjappai* sp. nov. LSID: urn:lsid:zoobank.org:act:924C6AC3-6407-4CB5-B00C-0619C6645D97 *Raorchestes vellikkannan* sp. nov. LSID: urn:lsid:zoobank.org:act:3E3E9E2A-E489-4AAD-8975-275BF0008CCB.
