# Supplementary material for: An integrative approach to infer systematic relationships and define species groups in the shrub frog genus Raorchestes, with description of five new species from the Western Ghats, India
Source: PeerJ. 2021 Mar 3;9:e10791. doi: 10.7717/peerj.10791 (PMC7936570; doi:10.7717/peerj.10791)
Supplement: Supplemental Information 1 [file peerj-09-10791-s001.pdf]

SUPPLEMENTAL INFORMATION

Manuscript title: An integrative approach to infer systematic relationships and define species groups in the shrub frog genus *Raorchestes*, with description of five new species from the Western Ghats, India

Authors: Sonali Garg, Robin Suyesh, Sandeep Das, Mark A Bee & SD Biju

Table S1 List of taxa and DNA sequences included in the study.

S.N.	Taxa	Collection Locality	Voucher Number	GenBank Accession Numbers			
				Mitochondrial		Nuclear	
				12SrRNA, tRNA^VAL^, 16SrRNA	*CYTB*	*RHOD*	*TYR*
	**Outgroup**						
1	*Limnonectes magnus*	Philippine Islands	0965	DQ347022, DQ347314	–	DQ347373	DQ347157
	**Rhacophoridae**						
2	*Buergeria oxycephala*	China: Hainan	SCUM 050267YJ	EU215524	–	EU215556	EU215585
3	*Beddomixalus bijui*	India: Kadalar, Idukki, Kerala	SDBDU 2011.1006	KU170017, KU169995	–	KU169946	–
4	*Chirixalus doriae*	China: Simao, Yunnan	KIZ060821034	EF564444, EF564516	EU924595	EU924539	EU924567
5	*Chiromantis xerampelina*	Africa	MVZ234606	GQ204785, GQ204734	GQ204551	–	–
6	*Feihyla palpebralis*	Vietnam: Lam Dong	KIZ 712	GQ285681	–	GQ285792	GQ285810
7	*'Nasutixalus' jerdonii*	India: Mawphlang, Meghalaya	SDBDU 2009.1166	KU230455, KU169999	–	KU169950	–
8	*Ghatixalus variabilis*	India: Mukkurthi NP, Nilgiris, Tamil Nadu	SDBDU 2008.4409	KU170006, KU169981	–	KU169932	–
9	*Gracixalus gracilipes*	China: Pingbian, Yunnan	KIZ060821196	EF564451, EF564523	EU924593	EU924537	EU924565
10	*Kurixalus eiffingeri*	Taiwan: Nan-Tou, Lu-Gu Chi-Tou	UMFS 5969	DQ283122	–	DQ283830	DQ282931
11	*Leptomantis bimaculatus*	Philippines: Tampakan, Cotabato Prov, Mindanao	ACD 5395	KF933272, –	–	KF933203	–
12	*Liuixalus romeri*	China: Mt. Shiwan, Guangxi	KIZ 061205YP	EU215528	–	EU215559	EU215589
13	*Mercurana myristicapalustris*	India: Chathankod, Thiruvananthapuram, Kerala	SDBDU 2011.849	KU170016, KU169994	–	KU169945	–
14	*Nyctixalus pictus*	Malaysia: Sarawak, Borneo	MVZ 239460	GQ204783, GQ204732	GQ204549	GQ204666	–
15	*Philautus aurifasciatus*	Indonesia: Java	ZRC1.1.5267	AY141805, GQ204702	GQ204519	GQ204640	–
16	*Pseudophilautus amboli*	"India"	CESF1011	JX092727, JX092658	JX092787	JX092982	JX092928
17	*Pseudophilautus cavirostris*	Sri Lanka	WHT3299	FJ788137, FJ788156	GQ204493	GQ204622	–
18	*Pseudophilautus kani*	"India"	CESF497	JX092754, JX092724	JX092820	–	JX092952
19	*Pseudophilautus limbus*	Sri Lanka: Haycock [Hiniduma]	WHT2700	AY141779, GQ204668	GQ204485	–	–
20	*Pseudophilautus microtympanum*	Sri Lanka: Central Hills	WHT5065	AF249030, GQ204678	GQ204495	AF249126	–
21	*Pseudophilautus poppiae*	Sri Lanka: Rakwana Hills	WHT2779	FJ788136, FJ788155	GQ204487	GQ204616	–
22	*Pseudophilautus sarasinorum*	Sri Lanka	WHT2481	AY141761, GQ204667	GQ204484	GQ204614	–
23	*Pseudophilautus schmarda*	Sri Lanka	WHT2715	AY880617, GQ204669	GQ204486	GQ204615	–
24	*Pseudophilautus wynaadensis*	India: Sultanbathery, Wayand, Kerala	VUB0070?	AF249031, AF249059	AF249087	AF249127	AF249190
25	*Polypedates leucomystax*	Indonesia: Java	ZRC1.1.5269	–, GQ204693	GQ204509	GQ204636	–
26	*Raorchestes agasthyaensis*	"India"	CESF492	–, JX092723	JX092785	JX092980	JX092926
27	*Raorchestes akroparallagi*	"India"	CESF061	JX092726, JX092650	JX092786	JX092981	JX092927

28	*Raorchestes anili*	"India"	CESF386	JX092730, JX092708	JX092791	JX092984	–
29	*Raorchestes archeos*	India: Agasthyamalai Massif, Western Ghats	CESF1190	–, JX092675	JX092789	JX092983	–
30	*Raorchestes aureus*	"India"	CESF1166	JX092745, JX092672	JX092811	JX092996	JX092945
31	*Raorchestes beddomii*	"India"	CESF072	JX092731, JX092653	JX092793	–	–
32	*Raorchestes blandus*	India: Anaimalai Massif, Western Ghats	CESF104	JX092725, JX092660	JX092781	–	–
33	*Raorchestes bobingeri*	"India"	CESF1238	JX092733, JX092680	JX092795	–	JX092930
34	*Raorchestes bombayensis*	"India"	CESF1010	–, JX092657	JX092796	JX092986	JX092931
35	*Raorchestes cangyuanensis*	China: Longchuan, Yunnan	5Rao	GQ285675	–	GQ285795	GQ285813
36	*Raorchestes chalazodes*	"India"	CESF1198	JX092734, JX092676	–	JX092988	JX092932
37	*Raorchestes charius*	"India"	CESF132	JX092736, JX092691	JX092799	JX092989	JX092933
38	*Raorchestes chlorosomma*	"India"	CESF1247	JX092770, JX092681	JX092780	JX092978	JX092923
39	*Raorchestes chotta*	"India"	CESF1003	JX092737, JX092656	JX092800	–	JX092934
40	*Raorchestes chromasynchysi*	"India"	CESF399	JX092741, JX092709	JX092804	–	JX092938
41	*Raorchestes coonoorensis*	"India"	CESF439	JX092740, JX092716	JX092805	–	JX092939
42	*Raorchestes crustai*	"India"	CESF1199	JX092742, JX092677	JX092806	–	JX092940
43	*Raorchestes drutaahu* sp. nov.	India: Kadalar, Idukki, Kerala	BNHS 6089	MW020034, MW020166	MW023233	MW023237	MW023241
44	*Raorchestes dubois*	"India"	CESF114	–, JX092668	JX092808	JX092993	JX092942
45	*Raorchestes echinatus*	India: Baba Budan Massif, Western Ghats	CESF1414	–, JX092696	JX092839	–	–
46	*Raorchestes flaviocularis*	India: Megamalai Massif , Peninsular India	CESF1251	–, JX092682	JX092809	JX092994	JX092943
47	*Raorchestes flaviventris*	India: Valparai Plateau, Anaimalai Massif	CESF1353	JX092746, JX092694	JX092813	–	–
48	*Raorchestes ghatei*	"India"	CESF1262	–, JX092687	JX092779	JX092977	JX092922
49	*Raorchestes glandulosus*	"India"	CESF1080	JX092744, JX092665	JX092810	JX092995	JX092944
50	*Raorchestes graminirupes*	"India"	CESF044	JX092772, JX092649	JX092812	–	JX092946
51	*Raorchestes griet*	"India"	CESF073	JX092747, JX092654	JX092814	JX092997	–
52	*Raorchestes gryllus*	Vietnam: Pac Ban, Tuyen Quang	ROM 30288	GQ285674	–	GQ285796	GQ285814
53	*Raorchestes honnametti*	India: Honnametti, BR Hills, Karnataka	BNHS 5941	–, KT151650	–	–	–
54	*Raorchestes indigo*	India: Kudremukh Massif, Western Ghats	CESF123	JX092749, JX092678	–	JX092999	JX092947
55	*Raorchestes jayarami*	"India"	CESF1260	JX092750, JX092686	JX092816	JX093000	JX092948
56	*Raorchestes johnceei*	"India"	CESF1236	JX092751, JX092679	JX092817	JX093001	JX092949
57	*Raorchestes kadalarensis*	"India"	CESF1766	–, JX092701	JX092818	JX093002	JX092950
58	*Raorchestes kaikatti*	"India"	CESF444	JX092752, JX092718	JX092822	JX093003	JX092951
59	*Raorchestes kakachi*	"India"	CESF1375	JX092765, JX092695	JX092843	–	–
60	*Raorchestes kakkayamensis* sp. nov.	India: Kakkayam, Kozhikode, Kerala	BNHS 6093	–, MW020167	MW023234	MW023238	MW023242
61	*Raorchestes keirasabinae* sp. nov.	India: Chathankod, Thiruvananthapuram, Kerala	BNHS 6097	–, MW020168	MW023235	MW023239	MW023243
62	*Raorchestes lechiya*	"India"	RL1601	–, KT359622	–	–	–
63	*Raorchestes leucolatus*	India: Elivalmalai Massif, Western Ghats	CESF1147	–, JX092669	JX092838	–	JX092968
64	*Raorchestes longchuanensis*	NA	KIZ048468	–, MN475870	–	–	–
65	*Raorchestes luteolus*	"India"	CESF1012	JX092756, JX092659	JX092823	JX093004	JX092954
66	*Raorchestes manohari*	"India"	CESF1187	–, JX092674	JX092824	JX093005	JX092955
67	*Raorchestes marki*	"India"	CESF467	JX092757, JX092719	JX092825	JX093006	JX092956
68	*Raorchestes menglaensis*	China: Yunnan	KIZ060821286	EU924626, EU924621	EU924600	EU924544	EU924572
69	*Raorchestes montanus*	"India"	CESF129	JX092759, JX092690	JX092827	–	JX092958

70	*Raorchestes munnarensis*	"India"	CESF094	–, JX092655	JX092828	JX093008	JX092959
71	*Raorchestes nerostagona*	"India"	CESF1061	JX092760, JX092661	JX092830	JX093009	JX092960
72	*Raorchestes ochlandrae*	"India"	CESF1111	JX092743, JX092666	JX092831	JX093010	JX092962
73	*Raorchestes 'parvulus'*	Thailand	KUHE:38322	LC012865	–	–	–
74	*Raorchestes ponmudi*	"India"	CESF063	JX092762, JX092651	JX092832	JX093011	JX092963
75	*Raorchestes 'hassanensis'*	"India"	CESF1178	JX092748, JX092673	JX092815	JX092998	–
76	*Raorchestes primarrumpfi*	India: Nilgiri Massif, Western Ghats	CESF441	–, JX092717	JX092833	JX093012	JX092964
77	*Raorchestes ravii*	India: Naduvattam, Nilgiris, Tamil Nadu	SDBDU 2013.2410	–, MW020169	–	–	–
78	*Raorchestes resplendens*	"India"	CESF1258	–, JX092683	JX092835	JX093013	JX092965
79	*Raorchestes rezakhani*	Bangladesh: Lawachara NP, Maulovibazar	JnUZool-A0419	–, MN072375	–	–	–
80	*Raorchestes sanjappai* sp. nov.	India: Periya, Wayanad, Kerala	BNHS 6100	–, MW020170	–	–	–
81	*Raorchestes shillongensis*	India: Malki forest, Shillong, Meghalaya	R2	–, MG980283	–	–	–
82	*Raorchestes signatus*	"India"	CESF425	JX092764, –	JX092836	JX093014	JX092966
83	*Raorchestes silentvalley*	"India"	RS1601	–, KT359628	–	–	–
84	*Raorchestes sushili*	"India"	CESF1259	JX092766, JX092684	JX092844	JX093018	JX092972
85	*Raorchestes theuerkaufi*	"India"	CESF1342	JX092767, JX092693	JX092845	–	–
86	*Raorchestes tinniens*	"India"	CESF438	–, JX092715	JX092846	–	JX092973
87	*Raorchestes travancoricus*	"India"	CESF473	JX092776, JX092721	JX092847	JX093019	JX092974
88	*Raorchestes tuberohumerus*	"India"	CESF148	–, JX092697	JX092848	–	–
89	*Raorchestes uthamani*	"India"	CESF483	–, JX092722	JX092849	JX093020	–
90	*Raorchestes vellikkannan* sp. nov.	India: Siruvani, Palakkad, Kerala	BNHS 6101	MW020035, MW020171	MW023236	MW023240	MW023244
91	*Rhacophorus reinwardtii*	Indonesia: Java	ZRC 1.1.5273	GQ204771, GQ204720	GQ204537	GQ204656	–
92	*Taruga fastigo*	Sri Lanka	WHT2783	AY141802, GQ204690	GQ204506	GQ204634	–
93	*Theloderma albopunctatum*	China: Jinping, Yunnan	KIZ060821201	EF564449, EF564521	EU924618	EU924562	EU924590
94	*Zhangixalus dugritei*	China: Baoxing, Sichuan	SCUM 051001L	EU215541	–	EU215571	EU215601

Table S2 Morphometric measurements for five new *Raorchestes* species described in the study. Measurement abbreviations and museum acronyms are provided in the Material and methods section. HT holotype; PT paratype; RS referred specimen. All measurements are in millimeters (mm).

Raorchestes drutaahu **sp. nov.**

Museum No	status	sex	SVL	HL	HW	SL	EL	TYD	NS	IUE	UEW	IN	FAL	HAL	FDIII	FWIII	TL	ShL	FOL	TFOL	TDIV	TWIV
BNHS 6088	HT	M	21.9	7.8	7.7	3.4	2.6	1.2	1.5	2.9	1.7	2.5	4.9	5.7	0.8	0.4	10.5	10.4	7.9	14.0	1.1	0.5
BNHS 6089	PT	M	21.1	7.3	7.4	3.1	3.2	0.9	1.5	2.7	2.0	2.4	4.2	6.1	1.0	0.5	10.1	10.0	7.6	12.7	1.1	0.5
BNHS 6090	PT	M	20.5	7.4	7.3	3.4	2.7	0.9	1.4	2.8	1.4	2.3	4.8	5.8	1.1	0.5	10.2	10.1	8.4	13.4	1.1	0.5
BNHS 6091	PT	M	22.4	7.8	7.7	3.8	2.4	1.1	1.5	2.9	1.8	2.4	4.8	5.6	1.3	0.6	12.1	11.5	9.6	15.6	1.2	0.6
		**Mean**	**21.5**	**7.6**	**7.5**	**3.4**	**2.7**	**1.0**	**1.5**	**2.8**	**1.7**	**2.4**	**4.7**	**5.8**	**1.1**	**0.5**	**10.7**	**10.5**	**8.4**	**13.9**	**1.1**	**0.5**
		**SD**	**0.8**	**0.3**	**0.2**	**0.3**	**0.3**	**0.2**	**0.1**	**0.1**	**0.3**	**0.1**	**0.3**	**0.2**	**0.2**	**0.1**	**0.9**	**0.7**	**0.9**	**1.2**	**0.0**	**0.1**
SDBDU 2015.3025	RS	F	24.5	9.2	8.7	3.7	2.9	1.3	1.6	3.1	1.9	2.4	5.6	6.4	1.1	0.6	12.6	12.6	9.6	17.2	1.0	0.5

Raorchestes kakkayamensis **sp. nov.**

Museum No	status	sex	SVL	HL	HW	SL	EL	TYD	NS	IUE	UEW	IN	FAL	HAL	FDIII	FWIII	TL	ShL	FOL	TFOL	TDIV	TWIV
BNHS 6092	HT	M	18.8	7.0	6.5	2.8	2.5	1.0	0.8	2.6	1.3	1.8	3.9	4.9	0.9	0.7	9.2	8.9	7.3	11.8	1.0	0.5
BNHS 6093	PT	M	17.8	6.1	5.8	2.6	2.0	0.7	0.7	2.5	1.4	1.6	4.0	4.6	0.8	0.6	9.1	8.6	6.6	10.7	0.9	0.7
BNHS 6094	PT	M	18.2	6.4	5.9	3.0	2.6	0.8	0.9	2.1	1.5	1.9	3.8	4.7	0.9	0.7	9.2	8.5	6.8	11.3	1.0	0.6
BNHS 6095	PT	M	17.5	6.1	5.8	2.9	2.4	0.8	0.9	2.7	1.4	1.7	3.9	5.0	1.0	0.6	9.1	8.5	6.7	11.4	0.8	0.5
BNHS 6096	PT	M	17.3	6.5	6.0	2.8	2.5	0.7	0.8	2.8	1.5	1.6	3.6	4.7	0.9	0.5	9	8.5	6.5	10.7	0.9	0.5
SDBDU 2019.3423	RS	M	18.4	6.6	5.7	2.6	2.1	0.7	0.9	2.6	1.4	1.8	3.4	4.7	0.9	0.7	9.1	8.1	6.5	10.6	0.9	0.7
		**Mean**	**18.0**	**6.5**	**6.0**	**2.8**	**2.4**	**0.8**	**0.8**	**2.6**	**1.4**	**1.7**	**3.8**	**4.8**	**0.9**	**0.6**	**9.1**	**8.5**	**6.7**	**11.1**	**0.9**	**0.6**
		**SD**	**0.6**	**0.3**	**0.3**	**0.2**	**0.2**	**0.1**	**0.1**	**0.2**	**0.1**	**0.1**	**0.2**	**0.2**	**0.1**	**0.1**	**0.1**	**0.3**	**0.3**	**0.5**	**0.1**	**0.1**

Raorchestes keirasabinae **sp. nov.**

Museum No	status	sex	SVL	HW	HL	SL	EL	TYD	NS	IUE	UEW	IN	FAL	HAL	FDIII	FWIII	TL	ShL	FOL	TFOL	TDIV	TWIV
BNHS 6097	HT	M	29.6	12.1	11.3	4.5	4.4	1.4	1.4	2.9	2.9	2.8	5.8	8.9	1.9	0.9	15.7	14.5	12.9	20.3	1.6	0.7
BNHS 6098	PT	M	30.5	12.2	11.6	4.9	4.8	1.9	1.5	3.8	2.8	2.3	6.7	8.5	1.7	0.7	16.8	15.9	11.9	19.2	1.7	0.6
SDBDU 2019.3450	RS	M	30.3	12.7	11.2	4.5	4.5	1.6	1.4	3.2	2.3	2.6	6.3	9.7	1.6	0.8	15.8	15.6	13	21.1	1.6	0.7
		**Mean**	**30.1**	**12.3**	**11.4**	**4.6**	**4.6**	**1.6**	**1.4**	**3.3**	**2.7**	**2.6**	**6.3**	**9.0**	**1.7**	**0.8**	**16.1**	**15.3**	**12.6**	**20.2**	**1.6**	**0.7**
		**SD**	**0.5**	**0.3**	**0.2**	**0.2**	**0.2**	**0.3**	**0.1**	**0.5**	**0.3**	**0.3**	**0.5**	**0.6**	**0.2**	**0.1**	**0.6**	**0.7**	**0.6**	**1.0**	**0.1**	**0.1**

Raorchestes sanjappai **sp. nov.**

Museum No	status	sex	SVL	HW	HL	SL	EL	TYD	NS	IUE	UEW	IN	FAL	HAL	FDIII	FWIII	TL	ShL	FOL	TFOL	TDIV	TWIV
BNHS 6099	HT	M	23.9	9.2	8.4	3.7	2.6	-	1.4	3.4	2.1	2.4	5.0	6.8	1.3	0.5	12.0	12.2	9.0	15.1	1.2	0.7
BNHS 6100	PT	M	22.2	8.7	7.9	3.4	2.9	-	1.3	3.6	1.6	2.4	5.3	6.7	1.3	0.6	11.9	12.2	9.3	16.1	1.1	0.6
SDBDU 2019.3440	RS	M	22.5	9.1	8.1	3.8	2.7	-	1.3	3.5	2.2	2.3	5.1	6.7	1.2	0.5	19.9	12.1	9.2	15.6	1.2	0.6
		**Mean**	**22.9**	**9.0**	**8.1**	**3.6**	**2.7**	**-**	**1.3**	**3.5**	**2.0**	**2.4**	**5.1**	**6.7**	**1.3**	**0.5**	**14.6**	**12.2**	**9.2**	**15.6**	**1.2**	**0.6**
		**SD**	**0.9**	**0.3**	**0.3**	**0.2**	**0.2**	**-**	**0.1**	**0.1**	**0.3**	**0.1**	**0.2**	**0.1**	**0.1**	**0.1**	**4.6**	**0.1**	**0.2**	**0.5**	**0.1**	**0.1**

Raorchestes vellikkannan **sp. nov.**

Museum No	status	sex	SVL	HW	HL	SL	EL	TYD	NS	IUE	UEW	IN	FAL	HAL	FDIII	FWIII	TL	ShL	FOL	TFOL	TDIV	TWIV
BNHS 6101	HT	M	22.9	8.6	8.6	3.2	3.1	-	1.0	2.9	1.4	2.3	5.2	6.6	1.1	0.5	12.1	12.2	8.4	15.1	1.1	0.6
SDBDU 2015.3019	RS	M	22.2	8.4	8.5	3.2	3.2	-	1.0	3.0	1.5	2.2	5.3	6.7	1.0	0.6	12.2	12.2	8.3	15.3	1.1	0.7
		**Mean**	**22.6**	**8.5**	**8.6**	**3.2**	**3.2**	**-**	**1.0**	**3.0**	**1.5**	**2.3**	**5.3**	**6.7**	**1.1**	**0.6**	**12.2**	**12.2**	**8.4**	**15.2**	**1.1**	**0.7**
		**SD**	**0.5**	**0.1**	**0.1**	**0.0**	**0.1**	**-**	**0.0**	**0.1**	**0.1**	**0.1**	**0.1**	**0.1**	**0.1**	**0.1**	**0.1**	**0.0**	**0.1**	**0.1**	**0.0**	**0.1**

Table S3 Factor loadings, eigenvalues, and percent variance from Principal Component Analysis based on 20 morphometric variables for six species of the *Raorchestes bombayensis* group. Values in bold indicate variables with the highest loadings for principal components of eigenvalue >1.0.

Variable	PC 1	PC 2	PC 3	PC 4	PC 5	PC 6	PC 7	PC 8	PC 9	PC 10
SVL	**-0.978728**	0.059270	-0.008213	0.042508	0.022583	0.048127	0.024942	0.027014	0.114389	-0.048809
HL	**-0.930570**	0.009241	-0.051701	-0.169105	-0.031037	-0.087124	-0.088357	0.041486	-0.093907	-0.187530
HW	**-0.764868**	0.415622	0.064208	0.066883	0.249639	-0.226279	0.140257	-0.253232	-0.167681	-0.068232
SL	**-0.951545**	0.060572	-0.095439	-0.093702	-0.094898	0.051172	0.034391	0.117401	-0.105279	-0.074236
EL	**-0.814184**	0.037239	-0.092663	-0.419381	-0.042330	0.227819	-0.168871	-0.015117	-0.200127	0.105910
TYD	**-0.764837**	-0.182116	0.018119	-0.379583	0.411772	0.138969	0.057504	0.040756	0.151277	-0.098598
NS	**-0.821198**	0.134795	0.205980	0.429360	0.151439	0.057693	-0.064091	0.126649	-0.083374	0.079347
IUE	**-0.793287**	0.087966	0.128472	-0.232403	-0.377836	-0.324374	-0.003069	0.034899	0.073002	-0.019065
UEW	**-0.619617**	-0.015143	-0.749874	0.090270	0.111453	-0.057218	-0.012575	-0.065651	0.060189	0.101853
IN	**-0.834145**	0.019041	0.004360	0.156352	-0.195978	0.318135	0.351978	0.049235	-0.053970	-0.033587
FAL	**-0.900773**	0.109733	0.258412	0.053683	0.155308	-0.093675	-0.105701	0.149883	0.009517	0.061056
HAL	**-0.962047**	-0.018388	-0.011480	0.137536	-0.055328	0.077272	-0.100511	-0.050470	0.083629	-0.059375
FDIII	**-0.930227**	0.058464	0.153911	0.150215	-0.087210	0.072746	-0.181676	-0.088062	0.070189	0.020338
FWIII	-0.546133	**-0.731208**	0.328262	-0.041071	0.073715	0.017681	0.015399	-0.181916	-0.052148	0.061236
TL	**-0.899796**	-0.067215	-0.042124	-0.177968	0.085071	-0.199439	0.152202	0.134115	-0.047648	0.174187
SHL	**-0.927924**	0.007595	0.110494	-0.084460	-0.179311	-0.037485	0.146643	-0.100251	0.126506	0.106820
FOL	**-0.970039**	0.055911	0.005212	0.128146	0.134305	-0.053339	-0.016233	0.023645	0.068928	-0.000582
TFOL	**-0.980727**	0.131708	-0.026004	0.022790	0.027448	0.052939	-0.017716	-0.039565	0.065813	-0.014435
TD_{IV}	**-0.919709**	0.016507	-0.120893	0.065720	-0.201078	0.112661	-0.112602	-0.102816	-0.014585	0.010778
TW_{IV}	**-0.604905**	**-0.663750**	-0.211264	0.271758	-0.054185	-0.182240	-0.005403	0.072041	-0.080948	-0.086165
Eigenvalue	14.63135	1.25741	0.91844	0.81454	0.60244	0.46106	0.30175	0.21802	0.19171	0.14773
% Total variance	73.15677	6.28705	4.59219	4.07272	3.01220	2.30528	1.50873	1.09012	0.95854	0.73867
Cum Eigenvalue	14.63135	15.88876	16.80720	17.62175	18.22418	18.68524	18.98699	19.20501	19.39672	19.54445
Cumulative %	73.1568	79.4438	84.0360	88.1087	91.1209	93.4262	94.9349	96.0251	96.9836	97.7223

Variable	PC 11	PC 12	PC 13	PC 14	PC 15	PC 16	PC 17	PC 18	PC 19	PC 20
SVL	-0.015851	0.034410	0.032469	0.029547	-0.044994	0.040223	-0.082888	0.050543	-0.025081	-0.000021
HL	-0.098691	-0.139525	-0.081897	-0.015761	-0.050966	0.025460	-0.011848	-0.032132	0.000228	0.005950
HW	0.023483	0.042928	0.025450	-0.011603	-0.006792	-0.008210	0.009806	0.011380	-0.005542	-0.000601
SL	-0.020574	-0.001102	0.076913	0.047100	0.134779	-0.047379	-0.020356	-0.013947	-0.014610	0.005332
EL	0.110576	-0.001453	0.038135	-0.048202	-0.026741	0.018067	-0.005720	0.020281	0.003479	-0.006324
TYD	0.056758	0.070684	0.015617	0.013609	-0.015948	-0.001657	0.039214	-0.022279	-0.002816	0.007613
NS	0.115995	-0.026343	-0.009869	0.060905	-0.008215	0.043377	0.013321	-0.021848	-0.003559	0.021401
IUE	0.168031	0.056227	-0.056911	0.049922	-0.013737	-0.012440	0.006199	0.002056	0.004461	0.003140
UEW	0.054858	-0.029244	-0.075315	0.001077	-0.002914	-0.034897	-0.015328	-0.010668	-0.006337	0.008427
IN	0.030911	0.008237	-0.068534	-0.021703	-0.047138	-0.029513	0.005918	-0.001553	0.005527	-0.007186
FAL	-0.065068	0.066628	-0.087607	-0.129856	0.013234	-0.052544	-0.010242	0.016928	-0.002981	0.003210

HAL	0.013932	-0.100762	-0.027513	0.002634	0.044279	0.002384	0.073943	0.064333	-0.011764	-0.007442
FDIII	-0.031751	-0.021664	0.095683	0.036469	-0.083020	-0.076507	0.002867	-0.034238	-0.006475	-0.008812
FWIII	0.005296	-0.029611	-0.080052	0.047525	0.034081	-0.016315	-0.032755	0.002184	-0.001261	0.002318
TL	-0.146129	-0.029182	0.026171	0.079062	-0.034364	0.008166	0.030334	0.014283	-0.000078	-0.010664
SHL	-0.015561	-0.073510	0.074774	-0.111265	0.028280	0.037983	0.004509	-0.029004	-0.008886	0.015182
FOL	0.067007	-0.018264	-0.011591	-0.015342	0.053507	0.036538	-0.023153	-0.035483	0.013861	-0.040192
TFOL	-0.051829	-0.012146	0.036575	0.024733	0.017536	-0.006637	-0.020338	0.027700	0.055864	0.014870
TDIV	-0.140324	0.175852	-0.055818	0.024677	0.020737	0.047883	0.025248	-0.022668	-0.003086	0.000868
TWIV	0.032845	0.058498	0.098768	-0.064384	-0.030170	0.008100	0.013898	0.008965	0.006834	0.000358
Eigenvalue	0.12707	0.08929	0.07434	0.05735	0.04297	0.02317	0.01895	0.01468	0.00464	0.00308
% Total variance	0.63537	0.44646	0.37171	0.28673	0.21485	0.11583	0.09476	0.07342	0.02318	0.01542
Cum Eigenvalue	19.67153	19.76082	19.83516	19.89251	19.93548	19.95864	19.97760	19.99228	19.99692	20.00000
Cumulative %	98.3576	98.8041	99.1758	99.4625	99.6774	99.7932	99.8880	99.9614	99.9846	100.0000

Table S4 Scores for Discriminant Function Analysis (DFA) of Principal Components resulting from 20 morphometric variables for adult male specimens belonging to six species of the *Raorchestes bombayensis* group.

Variable	DFA root 1	DFA root 2	DFA root 3	DFA root 4	DFA root 5
PC 1	-4.5064	-0.15838	-0.075534	-0.07794	0.046739
PC 2	1.8700	-1.68585	0.184028	-1.14639	-0.002876
PC 3	-1.7754	0.15445	-0.211754	0.45431	-0.632186
PC 4	-0.0330	-1.57911	0.831893	0.59969	-0.439519
PC 5	-0.9002	0.46340	1.544143	0.38112	0.220019
PC 6	1.2657	-1.30132	-0.473676	0.62301	0.160105
PC 7	-0.6581	0.18944	-0.602658	-0.62166	0.102097
PC 8	0.3495	1.19479	0.153763	-0.27095	-0.596181
PC 9	-0.0936	-2.10564	0.433519	0.51094	0.139631
PC 10	-1.3746	-1.49736	0.328488	-0.41988	-0.465876
PC 11	-1.9513	-0.56935	-0.609825	0.75928	-0.202943
PC 12	1.4251	-1.41015	-0.357504	-0.33058	-0.138329
PC 13	0.7891	-0.26511	-0.624680	0.83613	0.162047
PC 14	1.8581	-0.32621	-0.172047	0.16733	0.128740
PC 15	-0.3600	1.09106	-0.278058	0.11191	-0.083639
PC 16	-0.6169	-0.60941	0.339897	-0.09510	-0.217175
PC 17	-0.3020	1.79848	0.350929	-0.27799	-0.454349
PC 18	1.1395	-0.84950	-0.512553	0.37901	0.290384
PC 19	0.0626	-0.49216	-0.392957	0.31443	-0.819148
PC 20	-1.0416	-0.44635	0.765591	-0.12680	-0.031930
Eigenvalue	309.1136	31.67621	9.219832	6.57174	2.052595
Cumulative Prop.	0.8619	0.95024	0.975952	0.99428	1.000000

Table S5 Classification matrices from Discriminant Function Analysis for six members of the *Raorchestes bombayensis* group.

	Species	Percent correct	(1) p = 0.16667	(2) p = 0.16667	(3) p = 0.20000	(4) p = 0.20000	(5) p = 0.13333	(6) p = 0.13333
1	*R. bombayensis*	100	5	0	0	0	0	0
2	*R. ghatei*	100	0	5	0	0	0	0
3	*R. kakkayamensis* sp. nov.	100	0	0	6	0	0	0
4	*R. leucolatus*	100	0	0	0	6	0	0
5	*R. sanctisilvaticus*	100	0	0	0	0	4	0
6	*R. tuberohumerus*	100	0	0	0	0	0	4
	Total	100	5	5	6	6	4	4
